# Supplementary material for: Selective toxicity of ascorbic acid and hydrogen peroxide on human tenon cells without harming scleral cells in vitro: A possible alternative to non-selective mitomycin C?
Source: PLoS One. 2025 Apr 2;20(4):e0320558. doi: 10.1371/journal.pone.0320558 (PMC11964264; doi:10.1371/journal.pone.0320558)

Supplementary Figure S1

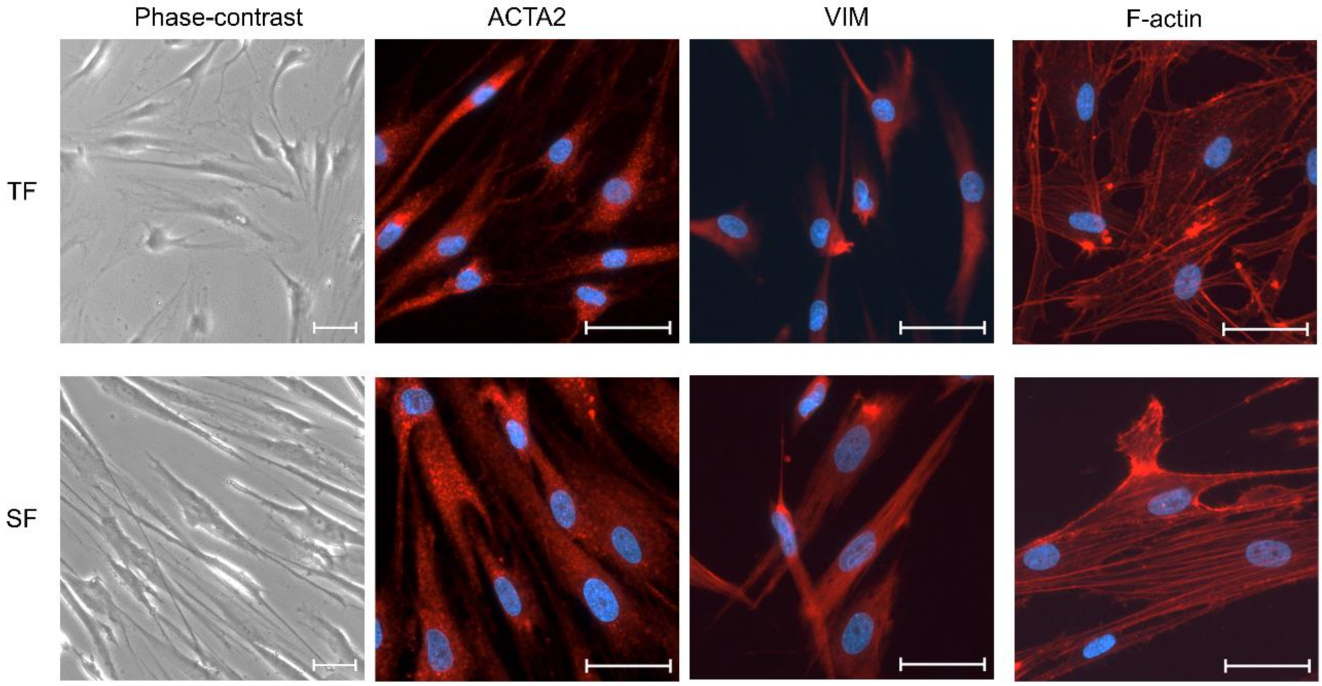

Supplementary Figure S2

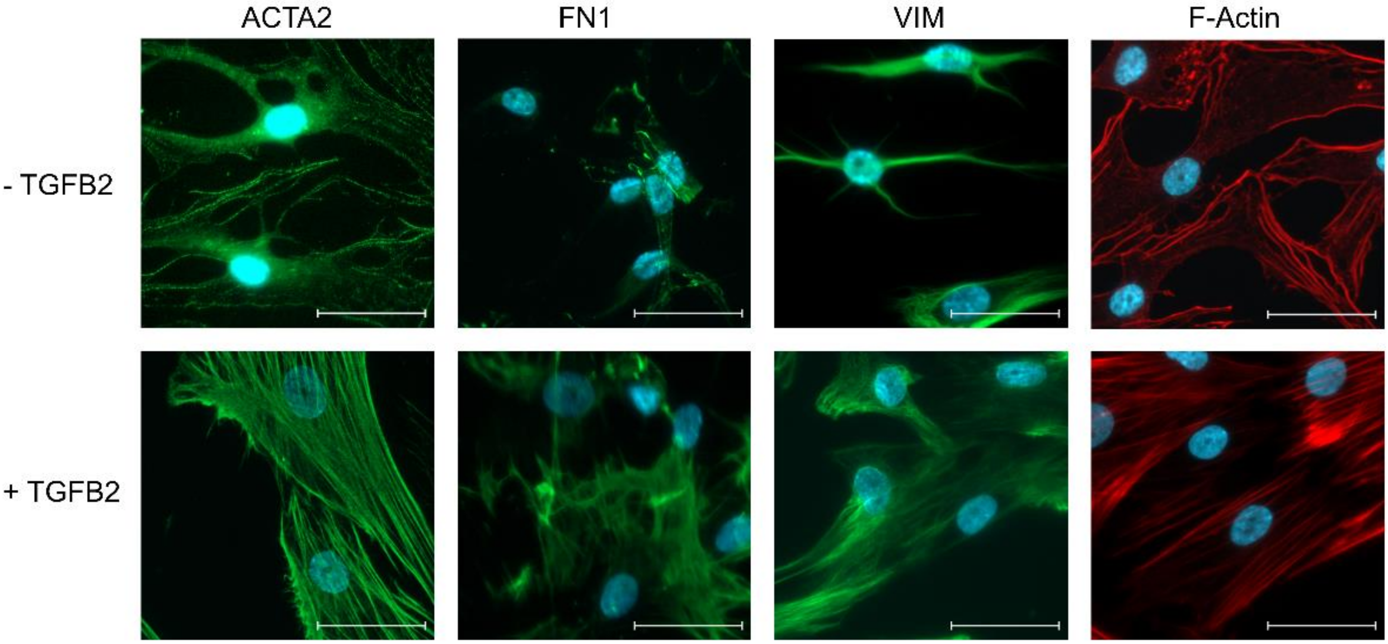

Supplementary Figure S3

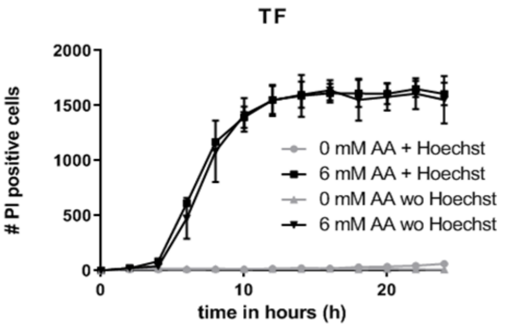

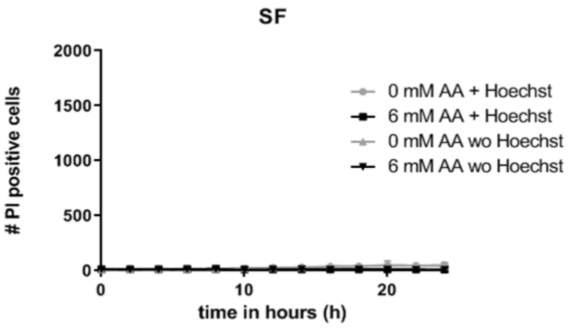

Supplementary Figure S4

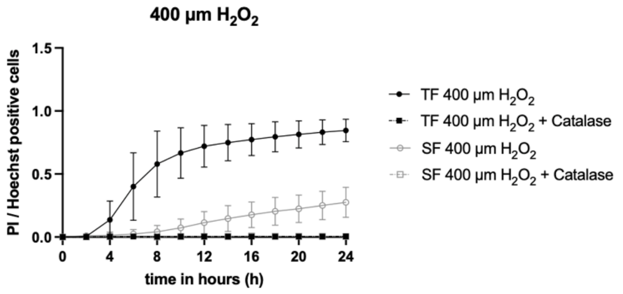

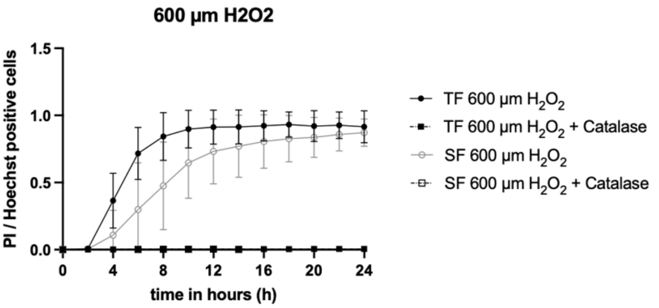

Supplementary Figure S5

A) Raw WB images used for Fig. 3 b and WB quantification

Stain-free blot

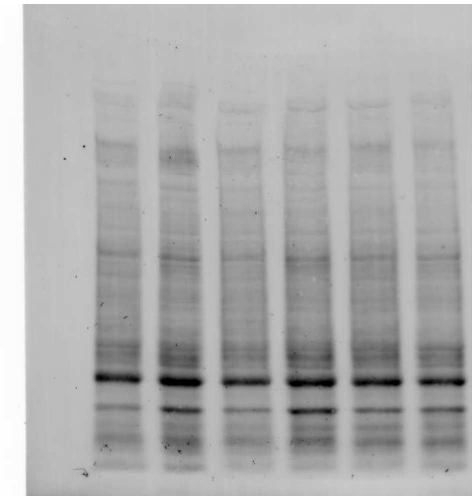

1. TF (Pat. 1)
2. SF (Pat. 1)
3. TF (Pat. 2)
4. SF (Pat. 2)
5. TF (Pat. 3)
6. SF (Pat. 3)

Lane: 1 2 3 4 5 6

Cy5 channel

250
150
100
75
50
37
25

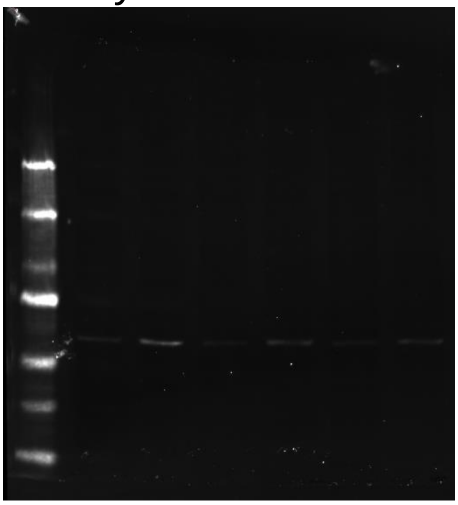

SB700 channel

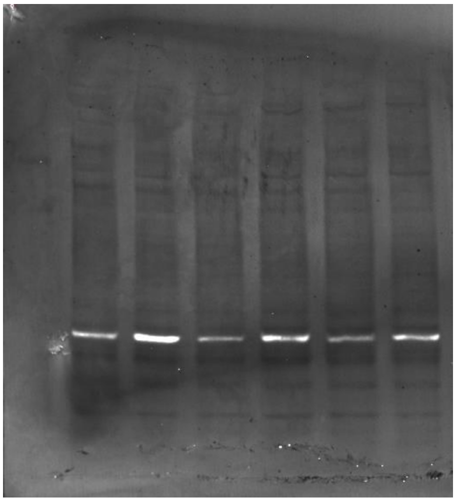

Catalase

Supplementary Figure S5

B) Raw WB images used for and WB quantification

Stain-free blot

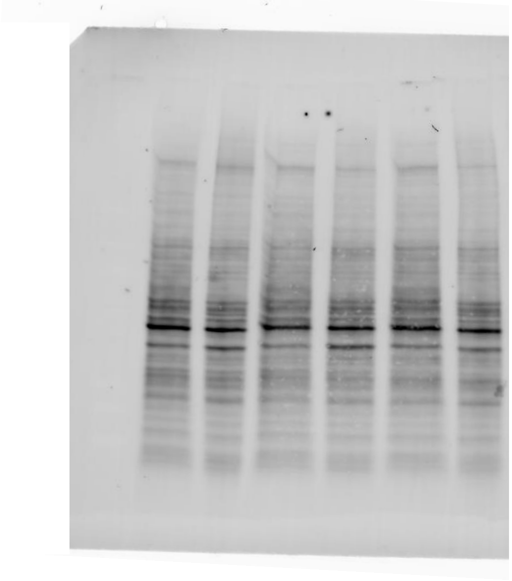

Lane: 1 2 3 4 5 6

1. TF (Pat. 1)
2. SF (Pat. 1)
3. TF (Pat. 2)
4. SF (Pat. 2)
5. TF (Pat. 3)
6. SF (Pat. 3)

Cy5 channel

250
150
100
75
50
37
25
20
15
10

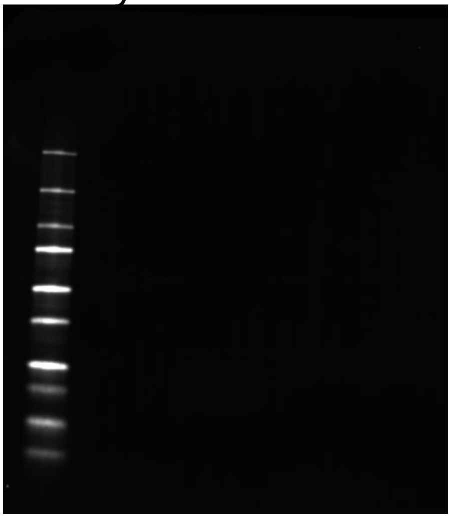

SB700 channel

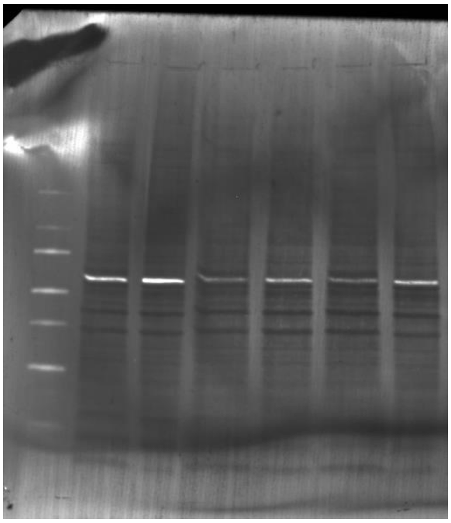

Catalase

Supplementary Figure S5
C) Raw WB images used for and WB quantification

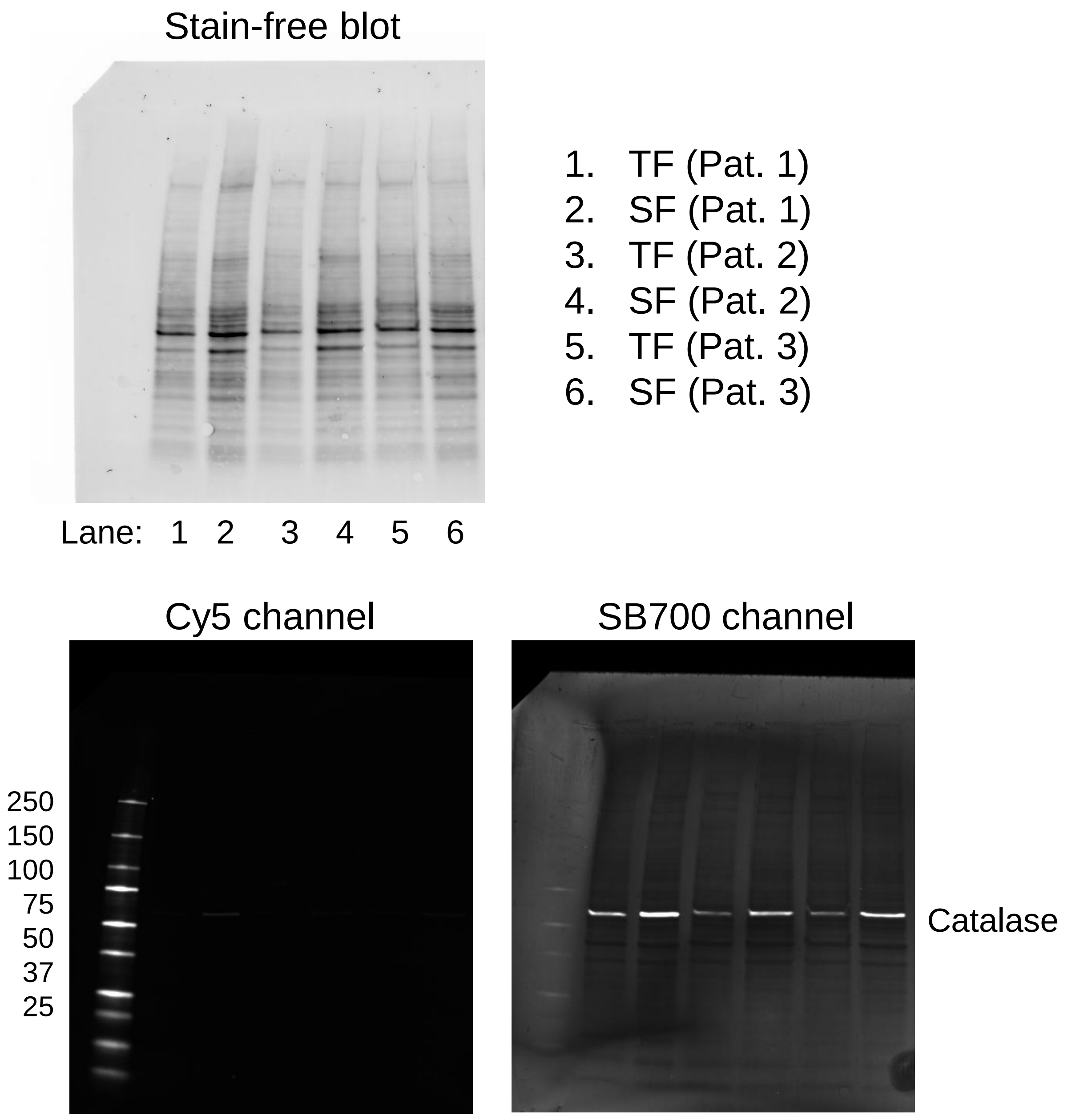

Supplement: S1 File — S1 Figure. Phase-contrast images and ICC-Staining for ACTA2, VIM, FN1, and F-Actin on human TFs and SFs. TFs (upper row) and SFs (lower row) at passage # 3 were cultured for 1 week and ICC-stained for ACTA2, VIM, or Phalloidin-Red to visualize F-Actin. Nuclei were stained with DAPI, and the scale bar represents 50 µm. S2 Figure. ICC-staining of human TFs and their ability to induce TGFB2-mediated endothelial-to-mesenchymal transition (EndoTM). Representative ICC results from human TFs treated without (upper row) or with recombinant TGFB2 (lower row) for one week. Cells were immunostained for ACTA2, FN1, VIM, or Phalloidin-Red to visualize F-Actin. The scale bar represents 50 µm. S3 Figure. Hoechst 33342 does not impact the AA-induced cell death. Cytotoxicity analysis of TFs (left) and SFs (right) exposed to 6 mM AA in combination with or without 28 nM Hoechst 33342. The y-axis shows the total number of PI-positive nuclei. S4 Figure. Catalase prevents hydrogen peroxide-induced cell death in both SFs and TFs. The relative numbers of dead TFs and SFs were calculated in two-hour intervals by dividing the number of PI-positive cells by the number of Hoechst-positive cells treated without or with 400 µ M (left) or 600 µ M (right) hydrogen peroxide without or with 500 Units Catalase for 24 h. S5 Figure: A-C) Raw WB images used for Fig. 3 b and WB quantification. (PDF) [file pone.0320558.s001.pdf]
