# Supplementary material for: Selective toxicity of ascorbic acid and hydrogen peroxide on human tenon cells without harming scleral cells in vitro: A possible alternative to non-selective mitomycin C?
Source: PLoS One. 2025 Apr 2;20(4):e0320558. doi: 10.1371/journal.pone.0320558 (PMC11964264; doi:10.1371/journal.pone.0320558)
Supplement: Attachment — Submitted filename: PONE-D-24-26851_R1_reviewer.pdf [file pone.0320558.s010.pdf]

PLOS ONE

Selective Toxicity of Ascorbic Acid and Hydrogen Peroxide on Human Tenon Cells without Harming Scleral Cells In Vitro: A Possible Alternative to Non-Selective Mitomycin C?

--Manuscript Draft--

Manuscript Number:	PONE-D-24-26851R1
Article Type:	Research Article
Full Title:	Selective Toxicity of Ascorbic Acid and Hydrogen Peroxide on Human Tenon Cells without Harming Scleral Cells In Vitro: A Possible Alternative to Non-Selective Mitomycin C?
Short Title:	Selective Cytotoxicity of Ascorbic Acid on Primary Human Tenon Fibroblasts
Corresponding Author:	Heiko Fuchs, Dr<br>Medizinische Hochschule Hannover<br>Hannover, GERMANY
Keywords:	glaucoma, fibrosis, ascorbic acid, tenon fibroblasts, sclera fibroblasts, catalase, Mitomycin C, live-cell imaging, hydrogen peroxide
Abstract:	Background: Glaucoma, a leading cause of blindness, is often driven by elevated intraocular pressure (IOP), which damages the optic nerve. Transscleral filtration surgery reduces IOP but is frequently complicated by excessive wound healing from Tenon fibroblasts (TFs), impeding aqueous humor absorption. Mitomycin C (MMC), used for over 30 years in ophthalmic surgeries, inhibits TF proliferation but carries significant side effects, including hypotony, blebitis, and endophthalmitis, due to its non-selective cytotoxicity. MMC's inability to entirely prevent fibrosis increases surgical failure risk, often necessitating further interventions like bleb needling. This study investigates whether ascorbic acid (AA) and hydrogen peroxide (H2O2) can selectively target TFs without damaging scleral fibroblasts (SFs) in vitro, using MMC as a benchmark.<br>Methods: Primary human TFs and SFs were cultured from patient trabeculectomy tissues. Cells were treated with various concentrations of MMC, AA, or H2O2. Cytotoxic effects were analyzed via live-cell imaging. Immunocytochemistry and Western Blot assessed catalase expression in both cell types and recombinant catalase was used to validate its protective effect against AA- and H2O2-induced cell death.<br>Results: Short-term exposure (5 min) to 0.02%-0.04% MMC or long-term exposure to 0.00025%-0.001% MMC caused cytotoxicity in TFs and SFs, with SFs dying significantly earlier. In contrast, AA (6–8 mM) selectively induced cell death in TFs without harming SFs. H2O2 also showed selective cytotoxicity towards TFs. Lower catalase expression in TFs compared to SFs was determined via Western blot and immunocytochemistry, highlighting a mechanism for this selective effect. Recombinant catalase neutralized the cytotoxic effects of AA and H2O2 on TFs.<br>Conclusions: Unlike MMC, Ascorbic acid and hydrogen peroxide exhibit selective cytotoxicity towards Tenon fibroblasts, which may provide a safer, more targeted approach for preventing fibrosis in glaucoma surgery. Additional in vivo studies are needed to explore the clinical applicability of these findings.
Order of Authors:	Heiko Fuchs, Dr
	Xiaonan Hu
	Roland Meister
	Yuqing Huang
	Martin C Bartram
	Amelie Pielen
	Bernd Junker
	Jan Tode

Powered by Editorial Manager® and ProduXion Manager® from Aries Systems Corporation

	Carsten Ralph Framme
**Response to Reviewers:**	Dear Reviewers,<br>We appreciate the thorough evaluation of our manuscript and the constructive feedback provided. Your comments have highlighted key areas for improvement. We have combined responses to overlapping comments to avoid redundancy and address each point comprehensively.<br>Response to both reviewers:<br>Reviewer #1: Methods and Results Sections: There is a significant overlap between the methods and results, particularly in the co-culture experiment section. Separating these sections will improve readability. I suggest clearly defining the methodology first and then explaining the results obtained.<br>Reviewer #2: Clear Separation of Methods and Results: There is a mix of methods and results, particularly in the co-culture experiment section involving Tenon fibroblasts (TFs) and scleral fibroblasts (SFs). These sections should be kept distinct to enhance the structure and flow of the manuscript.<br>Response:<br>We thank the reviewers for this comment. We acknowledge the suggestion to improve the separation of methods and results. However, in the case of the co-culture experiment, we intentionally provided detailed descriptions of the experimental setup in the results section to ensure that readers unfamiliar with IBIDI co-culture inserts can easily understand how both Tenon and scleral cells were spatially separated within one well. This co-culture system mimics the natural environment of these cells in the eye, where Tenon and scleral fibroblasts are neighboring but not mixed. Since MMC exposure occurs for both cell types simultaneously during surgery, we felt it was crucial to present this information alongside the results to avoid confusion and unnecessary back-and-forth between the methods and results sections.<br>Furthermore, we included images of the IBIDI inserts and the methodology for live-cell imaging to demonstrate how both cell types were analyzed separately. This made the data more comprehensible and underscored our commitment to transparency. We believe that this approach offers a more transparent narrative for the reader, especially those less familiar with such experimental setups, and reassures them of the validity of our findings.<br>Reviewer #1: Rationale for Compound Selection: The choice of using hydrogen peroxide and ascorbic acid requires more explanation. Why were these compounds chosen, and what specific advantage do they offer over Mitomycin C (MMC)? The discussion should delve into the potential roles of these compounds in oxidative stress or ferroptosis.<br>Reviewer #2: Explanation for the Use of AA and H2O2: The rationale for choosing ascorbic acid and hydrogen peroxide is insufficiently explained. The authors should elaborate on why these compounds were selected and their advantages over MMC, particularly their role in inducing oxidative stress or ferroptosis.<br>Response:<br>We appreciate the opportunity to explain further the reasoning behind our selection of Ascorbic Acid (AA) and Hydrogen Peroxide (H2O2). During a compound screening as part of the Glaukom project, we aimed to identify substances that selectively induce cell death in Tenon fibroblasts (TFs) without affecting neighboring Scleral fibroblasts (SFs). The goal was to find an alternative to Mitomycin C (MMC), which does not selectively target TFs and can lead to postoperative complications.<br>During this screening, we identified inhibitors that triggered autophagy selectively in TFs without harming SFs. Autophagy, a process of controlled cell degradation, appeared to be a promising target. We conducted an in-silico analysis, which revealed that Ascorbic Acid had been reported to inhibit autophagy in specific cell types. Consequently, we hypothesized that AA could counteract the autophagic death induced by these inhibitors. Surprisingly, our experiments showed the opposite: AA exhibited a synergistic effect, accelerating the death of TFs instead of rescuing them. This unexpected outcome led us to investigate AA further as a stand-alone agent. Since the findings from the autophagy screening are still unpublished and will soon be submitted for publication, we cannot disclose the specific inhibitors identified. We kindly ask for your understanding on this point.<br>As for Hydrogen Peroxide's selection, it resulted from our observation that TFs express lower catalase levels than SFs, making them more vulnerable to oxidative stress. Our Western blot analysis confirmed this differential expression, prompting us to test whether H2O2 could induce selective cytotoxicity by exploiting the reduced catalase expression in TFs. Indeed, we found that H2O2 selectively damaged TFs while sparing

Powered by Editorial Manager® and ProduXion Manager® from Aries Systems Corporation

SFs.
As a result, the reduced catalase expression in TFs represents an Achilles' heel, opening the door for novel therapeutic approaches to glaucoma surgery. By contrast, MMC lacks this selectivity and is associated with severe complications, such as hypotony, blebitis, and scleral thinning. We are confident that AA and H2O2 offer significant advantages in terms of safety and selectivity and could be promising alternatives for preventing intraoperative or postoperative fibrosis.

Reviewer #1: Objective of MMC Discussion: The manuscript includes MMC in the introduction, but its connection to the study is unclear. Is the goal to find an alternative to MMC, or is it a comparative analysis? The objective should be clearly defined.

Reviewer #2: MMC Discussion Clarification: It is unclear if the goal of the study is to replace MMC or simply to compare it with AA and H2O2. The objective must be stated clearly in the introduction to avoid confusion.

Response:
We appreciate the reviewers' comments and the need for clarification regarding the role of MMC in this study. To address the concern, we would like to clearly state that the primary objective of our study is to explore whether Ascorbic Acid (AA) and Hydrogen Peroxide (H2O2) can offer a more selective alternative to MMC in targeting Tenon fibroblasts, without causing damage to surrounding cell types such as scleral fibroblasts. While MMC is included for comparison, the focus of our investigation is on identifying safer and more targeted treatments for glaucoma surgery.
In Germany, MMC is widely regarded as the gold standard for preventing fibrosis after glaucoma filtration surgery, despite its well-documented side effects, such as hypotony, blebitis, and endophthalmitis. MMC's non-selective mechanism damages Tenon fibroblasts and surrounding cells, including scleral fibroblasts. This lack of specificity contributes to complications that can significantly impact patient outcomes. Given these risks, there is an urgent need for safer alternatives.
One notable practical advantage of AA is its affordability, but this could also present a challenge in securing funding for clinical off-label studies. Due to the low financial return on investment, sponsors may have little incentive to fund trials, which could delay AA’s adoption in clinical practice. Despite these economic challenges, we believe AA’s potential benefits, including its safety profile and effectiveness, warrant further investigation.
To simulate the intraoperative application of MMC as commonly used in glaucoma surgeries, we employed a 5-minute exposure in our experiments. This exposure duration aligns with clinical practice, where MMC is typically applied at concentrations of 0.02%-0.04% for 2-5 minutes. By using this approach in our co-culture experiments with both Tenon and Scleral fibroblasts, we closely mimicked surgical conditions to examine the differential effects of MMC on these two cell types.
Our findings, including the Lethal Time 50 (LT50) analysis, demonstrated a significant difference in cell death kinetics between scleral fibroblasts (SFs) and Tenon fibroblasts (TFs). Specifically, scleral cells died faster than Tenon cells when exposed to MMC, reinforcing that MMC, while effective, lacks the cell selectivity required for targeted therapy in glaucoma surgery.
By employing a co-culture setup, we were able to precisely monitor the effects of MMC on both cell types under identical conditions. This experiment provided valuable insights into MMC's non-selective cytotoxicity and underscored the need to explore alternative agents, such as AA and H2O2, that could selectively target Tenon fibroblasts without harming surrounding tissues. These findings will be clearly articulated in the revised manuscript.
To avoid any misunderstanding and to clarify the study's objective, we have changed the title of our manuscript from "Selective Cytotoxicity of Ascorbic Acid and Hydrogen Peroxide on Primary Human Tenon Fibroblasts without Harming Scleral Fibroblasts In Vitro" to "Selective Toxicity of Ascorbic Acid and Hydrogen Peroxide on Human Tenon Cells without Harming Scleral Cells In Vitro: A Possible Alternative to Non-Selective Mitomycin C?" This title better reflects our aim to identify safer alternatives to MMC for potential clinical use.
Unfortunately, due to ethical considerations, we have not been able to conduct in vivo studies to date. However, we hope that after the publication of this study, we will have provided sufficient evidence to justify future animal studies or off-label clinical trials.

Powered by Editorial Manager® and ProduXion Manager® from Aries Systems Corporation

Reviewer #1: High Passage Number for TFs: Using passage 10 Tenon fibroblasts raises concerns since higher passages might behave differently than early passage cells. A justification for choosing this passage number is required to address potential concerns about altered cell behavior.
Reviewer #2: Passage Number for TFs and SFs: The use of passage 10 TFs is concerning, as high-passage fibroblasts may differ from early-passage cells in terms of behavior. Additionally, the passage number for scleral fibroblasts (SFs) is missing and should be included in the methods for consistency and reproducibility.
Response:
The mention of passage 10 in the results section refers only to the fact that Tenon cells could be successfully expanded until passage 10 and beyond, regardless of prior intraoperative MMC treatment. This finding was included to highlight that MMC did not have a lasting inhibitory effect on cell proliferation ex vivo, which raises valid concerns about its efficacy in clinical use. However, the experiments presented in the manuscript were not conducted with passage 10 cells.
Both reviewers may have overlooked the details in the Methods section regarding the passage numbers of the Tenon fibroblasts (TFs) and Scleral fibroblasts (SFs). We want to clarify that the cells used in our experiments were between passages 2 and 8, as clearly stated in the manuscript. This passage range was chosen to ensure optimal cell behavior and reproducibility.
Moreover, we used patient-matched Tenon and Scleral fibroblasts (SFs) within similar passage ranges to ensure consistency and reproducibility. We maintained a strict protocol to use only passage-matched cells with a variation of ±1 passage number. The results presented in the manuscript are based on three technical replicates for each concentration, and the experiments were repeated three 2 to 6 times, necessitating higher passage cells due to the large number of cells required for these experiments. Only early passage cells (e.g., passage 3 or 4) would not have provided sufficient material for replications and statistical analysis.
In addition, it is difficult to define a universal "limit" for the passage number beyond which primary cells become unsuitable for in vitro studies, as this can vary greatly depending on the cell type and cell culture conditions, such as seeding density. For example, fibroblasts are more robust and can be used at higher passages than more delicate cells, such as neurons. For consistency, the passage number for SFs will also be included in the revised manuscript.
Finally, the fact that we observed consistent results across multiple independent experiments, despite using higher passage numbers, strongly suggests that the effects we report, whether with MMC, AA, or H_2O_2, are not passage-number dependent.
However, to avoid confusion, we revised that section stating now:" Remarkably, two out of six tenon tissue samples were treated intraoperatively with 0.04 % MMC for 3 to 5 minutes before the tissue was removed, whereas the other four tenon tissues were removed before MMC was applied. However, an outgrowth of proliferating TFs was observed in all tenon tissue samples after 5-7 days, regardless of whether they were treated intraoperatively with MMC (Fig. 1a+b, Supplementary Video S1). We could expand successfully and culture TFs up to and beyond passage 10, regardless of intraoperative MMC treatment. This observation suggests that MMC did not exert a lasting antiproliferative effect in ex vivo conditions."
Reviewer #1: Statistical Methods: The use of a t-test in comparing more than two groups is not appropriate. A better statistical approach, such as ANOVA, should be applied when comparing three groups.
Reviewer #2: Statistical Analysis Issues: In some comparisons where more than two groups are analyzed, a t-test is used, which is inappropriate. A more suitable statistical method like ANOVA should be used.
Response:
We thank the reviewer for pointing out the issue regarding the statistical methods. We acknowledge that a t-test is not the optimal choice when comparing more than two groups. For comparisons involving multiple groups, we should indeed have applied a one-way ANOVA to ensure the correct statistical treatment of the data.
In the revised manuscript, we have replaced the t-tests with ANOVA where applicable and report the results accordingly. Additionally, for post hoc analysis, we will include appropriate tests (e.g., Tukey's HSD) to identify significant differences between specific groups, if needed.
During our re-evaluation of the statistical analysis, we noted an error in the calculation of significances for the Western blot quantification in Figure 4. Upon applying the appropriate ANOVA approach, we found discrepancies in the initial significance

Powered by Editorial Manager® and ProduXion Manager® from Aries Systems Corporation

results. We have corrected these calculations and revised Figure 4 accordingly to accurately represent the statistical outcomes.

Response to Reviewer #1

Reviewer #1: TGF-β Inclusion: The rationale for using Transforming Growth Factor-beta (TGF-β) is unclear. The authors should explain the reason for incorporating TGF-β, especially given its role in fibrosis.

Response:

We appreciate the reviewer's request for clarification. As described in the literature, TGF-β was incorporated into our experiments to demonstrate that the Tenon fibroblasts (TFs) used in the study respond to TGF-β by undergoing epithelial-mesenchymal transition (EMT). The response to TGF-β is a crucial marker of the ability of TFs to react to fibrotic stimuli. To confirm this, we conducted immunocytochemical (ICC) staining, revealing that after one week of exposure to TGF-β, the TFs show the typical expression and distribution of EMT markers such as ACTA2, fibronectin, and vimentin. These markers clearly showed that the TFs responded as expected under pro-fibrotic conditions.

We will clarify this rationale in the revised manuscript and provide more detail.

Reviewer #1: Omission in Methods: Line 241 references treatments with MMC, but this is missing from the methods section. All treatments and procedures should be clearly outlined for better transparency.

Response:

We appreciate the reviewer pointing out this omission in the methods section.

We apologize for this oversight and correct it in the revision, Stating:

“Mitomycin C (MMC) Treatment

For the intraoperative application of Mitomycin C (MMC), a concentration of 0.04% MMC was prepared and applied using a sponge. The MMC was left in contact with the tissue for three minutes to allow for adequate absorption. Following the application, the area was washed twice with 10 ml of Hanks' Balanced Salt Solution (HBSS) to remove any residual MMC.

For the co-culture experiments,…”

Reviewer #1: pH Measurement: Only the pH of the 8 mM AA solution is reported. It is important to confirm whether other concentrations maintained a stable pH, as this could affect the results.

Response:

We appreciate the reviewer's request for clarification regarding the pH of the AA solutions. The pH of the highest concentration of AA (8 mM) was measured to be 6.8, which prompted us to include an HCl control to verify that the reduction in pH did not cause cytotoxicity. This control experiment demonstrated that the observed cell death was not due to the pH drop but to the effects of AA.

Given that the pH of the 8 mM AA solution had no cytotoxic impact when mimicked by the HCl control, it stands to reason that AA solutions with pH values between 6.8 and 7.4 (which would correspond to the lower AA concentrations) would also not cause significant cytotoxicity based solely on pH changes.

We will update the manuscript to include this clarification and ensure the reasoning for focusing on the 8 mM concentration is clear.

Reviewer #1: Summary Revision: The summary contains information that does not directly reflect the study's results. The authors should revise the summary to focus only on their experimental findings.

We have revised the summary to ensure it directly reflects the experimental findings of the study, stating:

"Background: Glaucoma, a leading cause of blindness, is often driven by elevated intraocular pressure (IOP), which damages the optic nerve. Transscleral filtration surgery reduces IOP but is frequently complicated by excessive wound healing from Tenon fibroblasts (TFs), impeding aqueous humor absorption. Mitomycin C (MMC), used for over 30 years in ophthalmic surgeries, inhibits TF proliferation but carries significant side effects, including hypotony, blebitis, and endophthalmitis, due to its non-selective cytotoxicity. MMC's inability to entirely prevent fibrosis increases surgical failure risk, often necessitating further interventions like bleb needling. This study investigates whether ascorbic acid (AA) and hydrogen peroxide (H_2O_2) can selectively target TFs without damaging scleral fibroblasts (SFs) in vitro, using MMC as a benchmark.

Methods: Primary human TFs and SFs were cultured from patient trabeculectomy

	tissues. Cells were treated with various concentrations of MMC, AA, or H2O2. Cytotoxic effects were analyzed via live-cell imaging. Catalase expression in both cell types was assessed by immunocytochemistry and Western blot, and recombinant catalase was used to validate its protective effect against AA- and H2O2-induced cell death.<br>Results: Short-term exposure (5 min) to 0.02%-0.04% MMC or long-term exposure to 0.00025%-0.001% MMC caused cytotoxicity in TFs and SFs, with SFs dying significantly earlier. In contrast, AA (6–8 mM) selectively induced cell death in TFs without harming SFs. H2O2 also showed selective cytotoxicity towards TFs. Lower catalase expression in TFs compared to SFs was determined via Western blot and immunocytochemistry, highlighting a mechanism for this selective effect. Recombinant catalase neutralized the cytotoxic effects of AA and H2O2 on TFs.<br>Conclusions: Unlike MMC, Ascorbic acid and hydrogen peroxide exhibit selective cytotoxicity towards Tenon fibroblasts, which may provide a safer, more targeted approach for preventing fibrosis in glaucoma surgery. Further in vivo studies are needed to explore the clinical applicability of these findings."<br><br>Response to Reviewer #2<br><br>Reviewer # 2: Discrepancy in AA Results: The authors should address the discrepancy between their results and Jampel et al.'s study regarding the cytotoxicity of AA. A discussion on why the current study required higher concentrations of AA would help clarify the differences between findings.<br>Response:<br>We want to address the discrepancy between our study and the findings by Jampel et al. from 1990. Jampel's study tested ascorbic acid (AA) at concentrations approximating those found in the aqueous humor of patients, around 1.1 mM. His approach was based on the assumption that the physiological concentration of AA within the eye might inhibit Tenon fibroblast proliferation. However, the in vitro environment used in Jampel's study, focusing on low concentrations of AA, may not fully represent AA's potential for selective cytotoxicity on TFs.<br>In addition to this concentration difference, it is crucial to highlight that while Jampel observed impaired viability of Tenon fibroblasts, his study did not explicitly investigate the concentration thresholds at which AA becomes cytotoxic. His research primarily centered on the inhibitory effect of AA on cell proliferation rather than pinpointing specific cytotoxic concentrations that selectively target Tenon fibroblasts.<br>In contrast, our study explored a broader range of AA concentrations, identifying 6-8 mM as the most effective in achieving selective cytotoxicity of Tenon fibroblasts while sparing neighboring scleral fibroblasts. This concentration range offers a more targeted strategy for reducing fibrosis. Additionally, our use of live-cell imaging and co-culture experiments, alongside extended observation periods, allowed for a more comprehensive understanding of AA's selective impact.<br>By expanding beyond the concentrations tested by Jampel, we observed more pronounced selective cytotoxicity, which may not have been apparent at lower concentrations. This broader range of AA concentrations highlights its potential utility in glaucoma filtration surgeries, where higher doses could more effectively prevent excessive wound healing without harming adjacent tissues.<br>To address these points, we have revised the discussion section to clarify the differences between our findings and those of Jampel et al., specifically regarding the concentration-dependent cytotoxic effects of AA.<br>Lastly, we sincerely appreciate the reviewers' valuable insights and the opportunity to improve our manuscript. We believe that these revisions have strengthened our work, and we are hopeful that it meets the standards for publication in PLOS ONE and remain with kind regards.<br><br>Heiko Fuchs<br>Corresponding Author
**Additional Information:**	
**Question**	**Response**
**Financial Disclosure**<br><br>Enter a financial disclosure statement that	Yes

describes the sources of funding for the work included in this submission. Review the submission guidelines for detailed requirements. View published research articles from *PLOS ONE* for specific examples.<br><br>This statement is required for submission and **will appear in the published article** if the submission is accepted. Please make sure it is accurate.<br><br>**Funded studies**<br>Enter a statement with the following details:<br>• Initials of the authors who received each award<br>• Grant numbers awarded to each author<br>• The full name of each funder<br>• URL of each funder website<br>• Did the sponsors or funders play any role in the study design, data collection and analysis, decision to publish, or preparation of the manuscript?<br><br>Did you receive funding for this work?	
Please add funding details.<br>as follow-up to "**Financial Disclosure**<br><br>Enter a financial disclosure statement that describes the sources of funding for the work included in this submission. Review the submission guidelines for detailed requirements. View published research articles from *PLOS ONE* for specific examples.<br><br>This statement is required for submission and **will appear in the published article** if the submission is accepted. Please make sure it is accurate.	This work was partially funded by the "Zentrales Innovationsprogramm Mittelstand" (ZIM) Grant number ZF4603201AW8 of the Federal Ministry for Economic Affairs and Energy, Germany. The funders had no role in study design, data collection and analysis, decision to publish, or preparation of the manuscript

**Funded studies**<br>Enter a statement with the following details:<br>• Initials of the authors who received each award<br>• Grant numbers awarded to each author<br>• The full name of each funder<br>• URL of each funder website<br>• Did the sponsors or funders play any role in the study design, data collection and analysis, decision to publish, or preparation of the manuscript?<br><br>Did you receive funding for this work?"	
Please select the country of your main research funder (please select carefully as in some cases this is used in fee calculation).<br>as follow-up to "**Financial Disclosure**<br><br>Enter a financial disclosure statement that describes the sources of funding for the work included in this submission. Review the submission guidelines for detailed requirements. View published research articles from *PLOS ONE* for specific examples.<br><br>This statement is required for submission and **will appear in the published article** if the submission is accepted. Please make sure it is accurate.<br><br>**Funded studies**<br>Enter a statement with the following details:<br>• Initials of the authors who received each award<br>• Grant numbers awarded to each author<br>• The full name of each funder<br>• URL of each funder website<br>• Did the sponsors or funders play any role in the study design, data collection and analysis, decision to publish, or preparation of the manuscript?<br><br>Did you receive funding for this work?"	GERMANY - DE
**Competing Interests**	The authors have declared that no competing interests exist.

Use the instructions below to enter a competing interest statement for this submission. On behalf of all authors, disclose any competing interests that could be perceived to bias this work—acknowledging all financial support and any other relevant financial or non-financial competing interests.<br><br>This statement is required for submission and **will appear in the published article** if the submission is accepted. Please make sure it is accurate and that any funding sources listed in your Funding Information later in the submission form are also declared in your Financial Disclosure statement.<br><br>View published research articles from *PLOS ONE* for specific examples.<br><br>**NO authors have competing interests**<br><br>Enter: *The authors have declared that no competing interests exist.*<br><br>**Authors with competing interests**<br><br>Enter competing interest details beginning with this statement:<br><br>*I have read the journal's policy and the authors of this manuscript have the following competing interests: [insert competing interests here]*<br><br>* typeset	
**Ethics Statement**<br><br>Enter an ethics statement for this submission. This statement is required if the study involved:<br><br>• Human participants<br>• Human specimens or tissue<br>• Vertebrate animals or cephalopods<br>• Vertebrate embryos or tissues	Tenon and scleral tissues were obtained from six patients who underwent trabeculectomy. The procedures were carried out following the tenets of the Declaration of Helsinki, following the guidelines and regulations of and with the approval of the ethics committee of Hannover Medical School (File number Nr. 8220_BO_K_2018). Written informed consent was obtained from all subjects.

• Field research<br><br>Write "N/A" if the submission does not require an ethics statement.<br><br>General guidance is provided below. Consult the submission guidelines for detailed instructions. **Make sure that all information entered here is included in the Methods section of the manuscript.**	

Powered by Editorial Manager® and ProduXion Manager® from Aries Systems Corporation

**Format for specific study types**<br><br>**Human Subject Research (involving human participants and/or tissue)**<br>• Give the name of the institutional review board or ethics committee that approved the study<br>• Include the approval number and/or a statement indicating approval of this research<br>• Indicate the form of consent obtained (written/oral) or the reason that consent was not obtained (e.g. the data were analyzed anonymously)<br><br>**Animal Research (involving vertebrate animals, embryos or tissues)**<br>• Provide the name of the Institutional Animal Care and Use Committee (IACUC) or other relevant ethics board that reviewed the study protocol, and indicate whether they approved this research or granted a formal waiver of ethical approval<br>• Include an approval number if one was obtained<br>• If the study involved *non-human primates*, add *additional details* about animal welfare and steps taken to ameliorate suffering<br>• If anesthesia, euthanasia, or any kind of animal sacrifice is part of the study, include briefly which substances and/or methods were applied<br><br>**Field Research**<br><br>Include the following details if this study involves the collection of plant, animal, or other materials from a natural setting:<br>• Field permit number<br>• Name of the institution or relevant body that granted permission	
**Data Availability**<br><br>Authors are required to make all data underlying the findings described fully available, without restriction, and from the time of publication. PLOS allows rare exceptions to address legal and ethical concerns. See the PLOS Data Policy and FAQ for detailed information.	Yes - all data are fully available without restriction

A Data Availability Statement describing where the data can be found is required at submission. Your answers to this question constitute the Data Availability Statement and **will be published in the article**, if accepted.<br><br>**Important:** Stating 'data available on request from the author' is not sufficient. If your data are only available upon request, select 'No' for the first question and explain your exceptional situation in the text box.<br><br>Do the authors confirm that all data underlying the findings described in their manuscript are fully available without restriction?	
**Describe where the data may be found in full sentences. If you are copying our sample text, replace any instances of XXX with the appropriate details.**<br><br>• If the data are **held or will be held in a public repository**, include URLs, accession numbers or DOIs. If this information will only be available after acceptance, indicate this by ticking the box below. For example: *All XXX files are available from the XXX database (accession number(s) XXX, XXX.*).<br>• If the data are all contained **within the manuscript and/or Supporting Information files**, enter the following: *All relevant data are within the manuscript and its Supporting Information files.*<br>• If neither of these applies but you are able to provide **details of access elsewhere**, with or without limitations, please do so. For example:<br><br>*Data cannot be shared publicly because of [XXX]. Data are available from the XXX Institutional Data Access / Ethics Committee (contact via XXX) for researchers who meet the criteria for access to confidential data.*<br><br>*The data underlying the results presented in the study are available from (include the name of the third party*	All relevant data are within the manuscript and its Supporting Information files (PlosoneDataofFigures.xlsx and S1_raw_images.pdf).

and contact information or URL).<br>• This text is appropriate if the data are owned by a third party and authors do not have permission to share the data.<br><br>* typeset	
Additional data availability information:	

Manuscript

Click here to access/download;Manuscript;Manuscript_Plosone291024.docx

Selective Toxicity of Ascorbic Acid and Hydrogen Peroxide on Human Tenon Cells without Harming Scleral Cells *In Vitro*: A Possible Alternative to Non-Selective Mitomycin C?

Short Title: **Selective Cytotoxicity of Ascorbic Acid on Primary Human Tenon Fibroblasts**

Heiko Fuchs[1, #, *], Xiaonan Hu[1, *], Roland Meister[1], Yuqing Huang[1], Martin C. Bartram[1], Amelie Pielen[1,2], Bernd Junker[1,2] Jan Tode[1], Carsten Framme[1]

[1] Department of Ophthalmology, University Eye Hospital, Hannover Medical School, Carl-Neuberg-Straße 1, 30625 Hannover, Germany

[2] Maximilians-Augenklinik, Erlenstegenstraße 30, 90491 Nürnberg, Germany

[*] These authors contributed equally to this work.

[#] Correspondence: fuchs.heiko@mh-hannover.de

Background: Glaucoma, a leading cause of blindness, is often driven by elevated intraocular pressure (IOP), which damages the optic nerve. Transscleral filtration surgery reduces IOP but is frequently complicated by excessive wound healing from Tenon fibroblasts (TFs), impeding aqueous humor absorption. Mitomycin C (MMC), used for over 30 years in ophthalmic surgeries, inhibits TF proliferation but carries significant side effects, including hypotony, blebitis, and endophthalmitis, due to its non-selective cytotoxicity. MMC's inability to entirely prevent fibrosis increases surgical failure risk, often necessitating further interventions like bleb needling. This study investigates whether ascorbic acid (AA) and hydrogen peroxide (H_2O_2) can selectively target TFs without damaging scleral fibroblasts (SFs) *in vitro*, using MMC as a benchmark.

Methods: Primary human TFs and SFs were cultured from patient trabeculectomy tissues. Cells were treated with various concentrations of MMC, AA, or H_2O_2. Cytotoxic effects were analyzed via live-cell imaging. Immunocytochemistry and Western Blot assessed catalase expression in both cell types and recombinant catalase was used to validate its protective effect against AA- and H_2O_2-induced cell death.

Results: Short-term exposure (5 min) to 0.02%-0.04% MMC or long-term exposure to 0.00025%-0.001% MMC caused cytotoxicity in TFs and SFs, with SFs dying significantly earlier. In contrast, AA (6–8 mM) selectively induced cell death in TFs without harming SFs. H_2O_2 also showed selective cytotoxicity towards TFs. Lower catalase expression in TFs compared to SFs was determined via Western blot and immunocytochemistry, highlighting a mechanism for this selective effect. Recombinant catalase neutralized the cytotoxic effects of AA and H_2O_2 on TFs.

Conclusions: Unlike MMC, Ascorbic acid and hydrogen peroxide exhibit selective cytotoxicity towards Tenon fibroblasts, which may provide a safer, more targeted approach for preventing fibrosis in glaucoma surgery. Additional *in vivo* studies are needed to explore the clinical applicability of these findings.

Keywords

glaucoma, fibrosis, ascorbic acid, tenon fibroblasts, sclera fibroblasts, catalase, Mitomycin C, live-cell imaging, hydrogen peroxide

Introduction

In glaucoma implant surgery and classic trabeculectomy, the aqueous humor is directed through a surgically prepared channel into a sub-conjunctival located reservoir called a filtering bleb. The conjunctiva absorbs the aqueous humor, which lowers the elevated intraocular pressure (IOP) to prevent irreparable damage to the optic nerve. However, undesirable excessive wound healing processes, such as fibrosis, occur in numerous cases. Either a fibrotic tenon cyst forms, creating an absorption barrier at the site of the reservoir, or the surgically created fistula is blocked by proliferating cells that produce an extracellular matrix. Both wound-healing processes lead to insufficient absorption of aqueous humor and increased IOP. Wound healing originates from cells of the tenon layer, which spatially separates the sclera from the conjunctiva. To prevent fibrosis of tenon fibroblasts (TFs), parts of the tenon layer are often surgically removed. In addition, mitomycin C (MMC) is applied to inhibit the proliferation of TFs.

Chen and co-workers were the first to propose the intraoperative application of MMC in trabeculectomy with a supposedly poor outcome in an 8-year follow-up study in 1990[1]. They reported that MMC can prevent excessive wound healing and thus reduce intraocular pressure more efficiently for a more extended period. Jampel confirmed in 1992 that a 5-minute exposure of 0.01 % and 0.04 % MMC is sufficient to inhibit the proliferation of TFs within 24 h *in vitro* *[2]*. Intraoperatively, a sponge soaked with 0.02 - 0.04% MMC is inserted into the prepared conjunctival pocket above the sclera for 2 to 5 min and then rinsed with a saline solution to wash out excess MMC. MMC is a cytostatic drug that penetrates the cells and inhibits DNA replication, thus preventing cell proliferation and inducing apoptosis[3]. Severe DNA damage may lead to DNA mutation and cell death. Therefore, MMC is classified as a carcinogenic substance, and its medical application is controversial. Another disadvantage of MMC is that it does not selectively kill TFs and may induce corneal, scleral, and conjunctival cell apoptosis [4-7].

Severe side effects have been reported, such as postoperative wound healing disorders or the formation of an avascular filtering bleb, hypotony, endophthalmitis, and blebitis [8-11]. Another

retrospective study raises doubts that a 2 to 5-minute application of MMC significantly prevents a postoperative tenon cyst formation [12]. It is often reported that an excessive wound-healing process occurs despite the intraoperative application of these cytostatic drugs. A postoperative follow-up treatment, such as a bleb revision, might be necessary. In a bleb needling procedure, MMC or 5-Fluorouracil is often administered subconjunctivally as an adjuvant to restore the function of a fibrosing bleb[9]. The benefit of this postoperative administration of these cytostatic drugs is not undisputed [13]. Despite the above-described controversial effects, MMC has been used in ophthalmic surgery for over 30 years, probably due to a lack of alternatives [1, 14-16].

Also, about 30 years ago, in 1990, Jampel reported that 1.1 mM ascorbic acid (AA), a concentration found in aqueous humor, reduced the plating efficiency of cell suspensions of human Tenon's capsule fibroblasts by an average of 40% within 24 h *in vitro* [17]. Furthermore, he showed that 1.1 mM AA addition reduced cell number by approximately 14% in confluent tenon cultures. He speculated that if AA has a similar effect *in vivo*, it could lead to incomplete wound healing and thus contribute to successful glaucoma filtration surgery.

In contrast to MMC, we aimed to identify compounds that prevent excessive wound healing of TFs without harming scleral fibroblasts (SFs). Therefore, tenon and scleral tissues from six human patients who underwent trabeculectomy were isolated to establish primary cell cultures. These primary cell cultures were further used for drug screening experiments. Here, we investigated the impact of AA and hydrogen peroxide on TFs and SFs *in vitro*.

Material and Methods

Cell Culture

Tenon and scleral tissues were obtained from six patients who underwent trabeculectomy. The procedures were carried out following the tenets of the Declaration of Helsinki, following the guidelines and regulations of and with the approval of the ethics committee of Hannover

Medical School (File number Nr. 8220_BO_K_2018). Patient samples were collected from August 2019 to September 2020. Written informed consent was obtained from all subjects. Some Tenon tissues were excised before the intraoperative application of MMC, while others were removed afterward. Primary TFs were obtained from tenon tissue according to a modified protocol of Przekora et al.[18]. Briefly, tenon tissue was cultured as a tissue attachment explant in Minimum Essential Medium Eagle (Sigma #M8042, Saint Louis, MI, USA) containing 5 % FBS (PAN #P40-39500, Aidenbach, Germany), GlutaMAX™ (Thermo Fisher #35050061, Waltham, MA, USA), Penicillin/Streptomycin (Gibco #3505-038), supplemented with 5 ng ml^{-1} fibroblast growth factor 2 (FGF-2) (Peprotech #AF-100-18B) and 5 µg ml^{-1} insulin (Thermo Fisher #12585014) in a 12-well. An outgrowth of TFs from the tissue could be observed approximately three to four days later. After the initial cell culture, cells were cultivated in the same medium without FGF-2 or insulin. TFs were split at around 90% confluence and were cultured until passage number ten.

Sclera tissue was obtained during the trabeculectomy procedure when a small incision was made in the sclera to alleviate intraocular pressure. SFs obtained from scleral tissue were expanded following the protocol of Seko et al., except that scleral tissue was not cut into small pieces [19]. For SFs, Minimum Essential Medium Eagle (Sigma #M8042) supplemented with 5% FBS, GlutaMAX™ (Thermo Fisher #35050061), and Penicillin/Streptomycin (Gibco #3505-038) was used for cultivation. Cell outgrowth was observed after approximately one week. Cell passages were performed at about 90 % confluence. TFs and SFs between passages two and eight were used for the following experiments.

Transforming growth factor-beta 2 treatment

For TGFB2 treatment, $2x10^4$ TFs were seeded in a 24-well plate with 0.5 mL complete medium containing 5% FBS. After 24 h, the medium was exchanged with the medium that contained 2% FBS and 10 ng ml^{-1} human recombinant TGFB2 (Peprotech #100-35B). TFs were cultured for one week, with the medium replaced after four days.

Immunocytochemistry staining

For ICC staining, $2x10^4$ TFs and SFs were seeded on a 13 mm diameter slide coverslip (Glaswarenfabrik Karl Hecht #41001113, Bavaria, Germany) into each well of a 24-well plate with 0.5 mL complete medium. Before fixation, cells were washed twice with PBS (Carl Roth #1058.1, Baden-Württemberg, Germany). Cells were immediately fixed with 4% paraformaldehyde (Carl Roth #P087.6) for 20 min at room temperature (RT) and washed twice with PBS for 5 min. Blocking was performed for 1 h at RT with a blocking solution containing 5% goat serum (Millipore #S26-100ml, Burlington, MA, USA), 0.02% Tween®20 (Sigma #P9416) and 0.01% Triton™X-100 (Sigma #X100) in PBS. ACTA2 rabbit mAb (Cell Signaling #19245, Danvers, MA, USA), VIM rabbit mAb (Cell Signaling #5741), FN1 rabbit mAb (Cell Signaling #26836), and Catalase rabbit mAb (Cell Signaling #12980S) were diluted 1:1000 in the blocking solution and cells were incubated overnight at 4°C. Cells were then washed twice with PBS for 10 min at RT and incubated with either 1:500 diluted AlexaFluor™546 goat anti-rabbit (Invitrogen #A11035) or AlexaFluor™488 goat anti-rabbit (Invitrogen #A11034) and 4 µl ml^{-1} rhodamine-phalloidin (Invitrogen #R415) in PBST (0.1% Tween®20 in PBS) for 2 h at RT. After three washes in PBS for 10 min at RT, the coverslips were mounted upside down on a slide with Roti®-Mount FluorCare DAPI (Carl Roth #HP20.1). Phase-contrast and fluorescence images were recorded with an Observer Z.1 microscope (Carl Zeiss, Baden-Württemberg, Germany) using the ZEN-Blue analysis software (Carl Zeiss).

Western blot

$10x10^4$ TFs and SFs were cultured in 6-well plates with 2 mL complete medium. Sample preparation, Electrophoresis, blotting, and antibody incubation were performed as previously described by our group[20]. Cells were washed twice with PBS and lysed with 1x Laemmli Sample Buffer (Bio-Rad #1610747, Hercules, CA, USA) containing 1x protease inhibitor cocktail (Cell signaling #5871S). The lysates were mixed with 2-Mercaptoethanol (Sigma-

Aldrich #60-24-2), denatured at 70˚C for 10 min, centrifuged at 4000 rpm for 2 min, and stored at -20˚C. The samples were loaded on Mini-Protean TGX Stain-Free Gels (Bio-Rad #4568094). Briefly, after 80–100 V electrophoresis, the resolved proteins were transferred to an ethanol-activated Mini-size LF PVDF membrane (Bio-Rad #10026934) with Trans-Blot®Turbo™ Transfer System at 1.3 A, 25 V for 7 min. The PVDF membrane was blocked with 5% milk powder (Carl Roth #T145.2) in 1X Tris-buffered saline (TBS) at RT for 1 h, followed by incubation with 1:1000 diluted primary Catalase rabbit mAb (Cell signaling #12980S) overnight at 4˚C. After incubation with 1:4000 diluted goat anti-rabbit secondary antibody StarBright™ Blue 700 (Bio-Rad #12004162) at RT for 1 h, the fluorescent signal was detected with ChemiDoc MP Imaging System (Bio-Rad) at a measurement wavelength of 660–720 nm. ImageLab 6 software (Bio-Rad) was used for normalization and quantification.

Mitomycin C (MMC) Treatment

For the intraoperative application of Mitomycin C (MMC), a concentration of 0.04% MMC was prepared and applied using a sponge. The MMC was left in contact with the tissue for three minutes to allow for adequate absorption. Following the application, the area was washed twice with 10 ml of Hanks' Balanced Salt Solution (HBSS) to remove any residual MMC.

For the co-culture experiments, Tenon fibroblasts (TFs) and scleral fibroblasts (SFs) were seeded in 48-well plates using a 2-well IBIDI-cell culture insert (IBIDI #80209, Bavaria, Germany), with 1 x 10^4 cells per well for each cell type. After 24 hours, the inserts were removed, and fresh medium containing either 0.02% or 0.04% MMC (Sigma #M4287) in Hank's Balanced Salt Solution (HBSS, Carl Roth #9119.1) was applied to the wells for 5 minutes to mimic intraoperative MMC exposure. Subsequently, the medium was replaced with fresh medium containing 28 nM Hoechst 33342 (Thermo Fisher #H1399) and 2 µg/ml PI (Sigma #P4170) for live-cell imaging to monitor cytotoxic effects. In addition, for long-term exposure, MMC was directly added to the medium at concentrations of 0.000125%, 0.00025%,

or 0.001%, and the cells were exposed and monitored for 90 hours to simulate postoperative conditions.

Ascorbic acid treatment

In monoculture experiments, 3x10⁴ Tenon fibroblasts (TFs) or scleral fibroblasts (SFs) were seeded in 48-well plates with 250 µL of medium containing 2% FBS, 28 nM Hoechst 33342 (Thermo Fisher #H1399), and 2 µg/ml Propidium Iodide (PI) (Sigma-Aldrich #P4170). They were treated with different concentrations of Ascorbic Acid (AA) (Sigma #A0278) ranging from 0 to 8 mM. When 8 mM AA was added, the pH of the medium dropped from 7.7 to 6.8. To rule out the possibility that this pH drop caused cytotoxicity, an HCl control with a pH of 6.8 was included by adding 6.7 µL of 1N HCl (Carl Roth #4625.1) per mL of medium. The medium with HCl was sterilized using a 0.22 µm PES syringe filter (Carl Roth #P668.1). Live-cell imaging was then performed for 24 hours.

Hydrogen peroxide treatment

$1x10^4$ TFs and SFs were seeded and co-cultured as previously. 24 h later, the inserts were taken out, and the medium was changed to fresh medium containing 28 nM Hoechst 33342, 2 µg ml^{-1} PI, different hydrogen peroxide (Sigma #216763-100ML) concentrations (400-600 µM) together with or without 500U ml^{-1} catalase solution (Sigma #SLCJ5891). Live-cell imaging was performed for 24 h.

Rekombinant Catalase Treatment

In the co-culture experiments, $1x10^4$ TFs and SFs were seeded as described above. 24 h later, the inserts were taken out, and a fresh medium containing Hoechst, PI, and AA or Hydrogen peroxide was used with or without 400, 500, or 600 U ml^{-1} catalase solution. Live-cell imaging was then performed for 24 h.

Live-cell imaging

Live cell imaging experiments were performed using the BioTek® Lionheart™ FX Automated Microscope (Agilent Technologies, Santa Clara, CA, USA). The experiment setup and subsequent analysis were performed as previously described by our group using the Gen5 Image Prime 3.05 software[21]. The 48-well culture plate was placed in the humidity chamber with CO_2 levels set to 5% and temperature to 37°C. Imaging was carried out in the phase-contrast channel using the 4X PL FL phase objective and laser autofocus. In addition to the phase-contrast images, the DAPI fluorescence filter set was used to detect Hoechst-stained nuclei, and the RFP fluorescence filter was used to detect PI-positive nuclei. Images were recorded for 48 h at 20-min intervals. The acquisition parameters for the phase contrast channel were set to 10 "LED",100 "Integration time" and 4.8 "gain". For DAPI, the values were set to 6 "LED",71 "Integration time" and 24 "gain". For RFP, the values were set to 10 "LED",120 "integration time" and 21 "gain". The relatively high gain settings were used to keep the exposure time as short as possible to minimize photobleaching during live-cell imaging.

Live-cell imaging analysis

The number of Hoechst-positive objects per image was determined using the cell count option of Gen5 analysis software, as previously described[21]. The analysis tool was briefly used, and the channel "DAPI 377, 4747" was selected under "Primary mask and Count". The "Threshold" was set to 2000, the background to "Dark", and the options "Split touching objects" and "Fill holes in masks" were selected. In addition, under "Advanced Detection Options", a "Background flattening" with a "Rolling Ball diameter" of 30 µm, an "Image smooth strength" of 2 "Cycles of 3x3 average filter", and "Evaluate background on 5 % of lowest pixels" were selected. Under "Object selection", a "Min. object size" of 10 µm and a "Max. object size" of 70 µm were specified, and the option "Include primary edge objects" was selected. For the non-co-culture experiments, the option "Analyse entire image" was selected. For the co-culture experiments, the "Plug" tool was used to place a rectangular window on the left side of the image to count the TFs and a rectangular window on the right side to calculate the SFs. Both

windows had a size of 750 x 1500 µm. The number of PI-positive objects was calculated using the analysis tool to count dead cells. Therefore, under "Primary mask and Count", the channel "RFP 531, 593" was selected, a threshold of the positive PI signal was set, and the parameters previously described in the DAPI channel were used for further analysis. The data was further analyzed using Microsoft Excel 2010. The number of PI-positive cells was divided by the number of Hoechst-positive nuclei to determine the percentage of dead cells per area for each time point. The mean and standard deviation of biological replicates were calculated from these values with Graph Pad Prism 9.

Statistical analysis

All experiments were performed in at least three independent replicates, and data were analyzed using Microsoft Excel 2010 and GraphPad Prism 9. A one-way ANOVA was used to determine statistical significance and compare catalase expression between Tenon fibroblasts (TFs) and Scleral fibroblasts (SFs). Post hoc analysis used Tukey's HSD test to compare the groups. Data are presented as mean ± standard deviation (SD) with significance thresholds set at **$p < 0.01$**, and ***$p < 0.001$**.**

Results

Effects of MMC on TFs *ex vivo /in vitro*

Tenon and scleral explants were collected from six trabeculectomy patients. The explants were transferred into a cell culture medium to allow TF and SF to outgrow, according to a modified protocol of Przekora and Seko[18, 19]. Phase contrast images and immunocytochemistry (ICC) staining for vimentin (VIM), actin alpha 2 (ACTA2), and F-actin were obtained from the isolated TFs and SFs (Supplementary Fig. S1). Furthermore, it was tested whether TFs transform into myofibroblasts after one week of transforming growth factor beta 2 (TGFB2) exposure, as reported previously by other groups [22-24]. Indeed, TGFB2-treated TFs show a

more widespread distribution of VIM within the cytoplasm, an increase of ACTA2, an increase of the extracellular matrix protein fibronectin 1 (FN1), and a resemble of the F-Actin, compared to the untreated TFs (Supplementary Fig. S2). Remarkably, two out of six tenon tissue samples were treated intraoperatively with 0.04 % MMC for 3 to 5 minutes before the tissue was removed. In contrast, the other four tenon tissues were removed before MMC was applied. However, an outgrowth of proliferating TFs was observed in all tenon tissue samples after 5-7 days, regardless of whether they were treated intraoperatively with MMC (Fig. 1a+b, Supplementary Video S1). We successfully expanded and culture TFs up to and beyond passage 10, regardless of intraoperative MMC treatment. This observation suggests that MMC did not exert a lasting antiproliferative effect in ex vivo conditions.

Figure 1. Cytotoxic effects of MMC on human TFs and SFs *in vitro*.
Representative phase-contrast images showing TF cells at days 6 and 7 after trabeculectomy and initial tenon tissue culture, respectively, treated intraoperatively without (**a**) or with MMC (**b**). The scale is 1000 µm. (**c**) Co-culture inserts were used to culture TFs and SFs separately within one well of a 48-well plate. (**d**) Representative fluorescent pictures of co-cultured TF and SF cells that were treated for 5 min with 0.04 % MMC at time point 74 h. During the experiment, cells were stained with Hoechst 33342 (upper row) and PI (middle row) to visualize dead cells. The right and left sections of the image (highlighted by the two white dashed rectangles) were analyzed to quantify the dead TF and SF cells separately. The total number of cells was determined by gating the Hoechst-stained nuclei for the analysis. This mask subsequently identifies PI-positive nuclei by setting a threshold in the red channel. In the lower row, PI-positive nuclei are highlighted in red circles, whereas PI-negative nuclei are highlighted in yellow circles. (**e**) Cytotoxicity analysis for 90 h of PI- and Hoechst-stained co-cultured TFs (left diagram) and SFs (right diagram) after treatment with 0.02 % and 0.04 % MMC in HBSS for 5 minutes. HBBS alone for 5 min was used as a control. After MMC treatment and before live-cell imaging, cells were cultured in regular growth media with Hoechst and PI. For the five-minute 0.04 % MMC treatment, the LT50, i.e., the time 50 % of the cells have died, is also given. (**f**) Cytotoxicity analysis of PI- and Hoechst-stained co-cultured TFs (left diagram) and SFs (right diagram) that were permanently exposed without or with 0.000125 %, 0.00025 %, 0.005 % or 0.001% MMC for 90 hours. Where possible, the LT50 for different MMC concentrations was specified here.

Short-term and long-term exposure to MMC on TFs and SF *in vitro*

Since TFs are in close contact with SFs in the eye, TFs and SFs were cultivated together in a 48-well using a co-culture insert (Fig. 1c). To test for a possible cytotoxic effect of MMC in the co-culture cell model, Hoechst 33342 was added to label the cell nuclei, and propidium iodide

(PI) to label dead cells. The Hoechst concentration of 28 nM and the acquisition parameters used for live-cell imaging were neither cytotoxic nor phototoxic, as described in previous studies[21, 25]. To mimic the intraoperative MMC application *in vitro*, TFs and SFs were treated for 5 minutes without or with 0.02 % or 0.04 % MMC. After a subsequent medium change with the addition of Hoechst and PI, the number of dead cells was determined over 90 hours using a live-cell imager and subsequent image analysis. To determine the percentage of dead cells, the number of PI-positive nuclei was divided by the number of Hoechst-stained nuclei for TFs and SFs separately (Fig. 1d), and the calculated lethal time 50 (LT_{50}) revealed that with a five-minute 0.04% MMC application, half of the TFs had died after 74 h. In contrast, the LT_{50} for SFs was only ~ 35.5 h (Fig. 1e).

In a further experiment, both cell types were permanently exposed to MMC for 90 hours but at lower concentrations between 0.001 % and 0.000125 % to simulate potential effects in cases where MMC is insufficiently washed out. The LT_{50} values for each MMC concentration confirmed the tendency for SFs to die faster from MMC than TFs (Fig. 1f). Representative time-lapse videos of the co-cultures exposed to either 0.04 % MMC for 5 min or 0.001 % MMC permanently are shown in the Supplementary Video S2.

Impact of AA on TFs and SFs *in vitro*

Since Jampel demonstrated in 1990 that 1 mM AA inhibits the viability of TFs in vitro, we wanted to test the effect of AA on both cell types. For this approach, confluent TFs or SFs were exposed to different AA concentrations between 0 and 8 mM for 24 h. Notably, the pH value of the 8 mM AA treatment condition decreased from 7.7 to 6.8. To exclude effects caused by pH decrease, an additional control was included with a medium adjusted to pH 6.8 using 1N hydrochloric acid (HCl). The percentage of dead TFs (left panel) and SFs (right panel) within 24 hours was determined by dividing the number of PI-positive cells by the total number of Hoechst-positive cells (Fig. 2A). Data reveals that AA concentrations of 6 and 8 mM resulted in complete cell death of TFs within 24 h. No significant cell death was observed in the

untreated cells or HCl control. Furthermore, no significant cytotoxic effect of AA on SFs was observed (Supplementary Video 3). No AA-induced cytotoxicity was observed in sclera cells after the 24 h window. Therefore, subsequent LCI analyses were performed in the 24-hour time window.

Figure 2. Low concentrations of AA selectively kill human TFs without harming SFs *in vitro*.

(**a**) The two bar diagrams show the percentage of dead TFs (left side) and SFs (right side), which were exposed to 0, 2, 4, 6, and 8 mM AA or HCl control at time point zero and after 24 h. (**b**) Cell toxicity analysis of monocultured TFs (right diagram) and SFs (left diagram) that were treated without or with 6 mM or 8 mM AA for 24 h. For data collection, live cell analysis was performed three times on TFs and SFs obtained from six trabeculectomy patients. (**c**) Representative phase-contrast and Hoechst and PI fluorescence images of monocultured TFs (left panel) and SFs (right panel) with 6 mM AA for 0 h (upper row) and 24 h (lower row). The scale bar represents 1000 µm. (**d**) Representative phase-contrast and Hoechst and PI fluorescence images of co-cultured TFs and SFs treated with 6 mM AA for 0 h (upper row) and 24 h (lower row). The scale bar represents 1000 µm. (**e**) Cell toxicity analysis of co-cultured TFs and SFs treated without or with 6 mM AA for 24 h.

To evaluate the possibility that AA-induced cell death was caused by nuclear staining with Hoechst, we treated TFs with 0 and 6 mM AA in combination with and without Hoechst in the medium. Live-cell imaging analysis showed that Hoechst alone did not cause cytotoxicity in the TFs within 24 hours, while the TFs treated with 6 mM AA died after about 12 hours without any synergistic effect caused by Hoechst staining (Supplementary Figure S3). To further exclude whether AA-induced cell death of TFs was possibly patient-specific or coincidental, we tested AA on TFs and SFs of six trabeculectomy patient samples. Therefore, we treated TFs and SFs from six trabeculectomy patients in monoculture experiments without or with 6 mM and 8 mM AA. In all 6 patient samples, AA concentrations between 6 and 8 mM were cytotoxic for TFs but harmless for SFs (Fig. 2b+c).

Since the tenon cell layer is located directly above the sclera *in vivo*, we wanted to investigate whether signaling molecules or free radicals might be released by AA-induced death of TFs, which could impair the viability of the SFs. For this purpose, we co-cultured TFs and SFs in one well of a two-chamber cell culture set and incubated them for 24 h. The insert was

removed, spatially separating both cell types, and treated with or without 6 mM AA with PI and Hoechst. In co-cultures, we observed that TFs ultimately died within 24 hours in the presence of 6 mM AA, whereas no significant cytotoxicity was observed for the SFs (Fig. 2d+e, Supplementary Video 4).

Catalase expression in TFs and SFs

Next, we wanted to investigate how AA selectively attacks the TFs without damaging the SFs. Numerous studies have shown that AA sometimes initiates the $Fe^{3+} \rightarrow Fe^{2+}$ redox cycle in the Fenton reaction. Fe^{2+} further reacts with hydrogen peroxide, increasing toxic hydroxyl radicals that can irreparably damage the cells. Consequently, the concentration of hydroxyl radicals formed depends on the amount of free Fe^{2+} and the concentration of hydrogen peroxide. The hydrogen peroxide concentration is regulated by endogenous catalase expression. Immunofluorescence staining revealed that both cell types express catalase within the cytoplasm (Fig. 3a). However, a Western blot analysis of TFs and SFs from three patients showed that the catalase protein levels are significantly reduced in TFs compared to SFs (Fig. 3b+c).

Figure 3. Unveiling a potential weak spot: TFs have lower catalase expression than SFs. (**a**) Representative ICC-staining showing catalase expression (green), F-Actin (red), and DAPI (blue) of TFs (upper row) and SFs (lower row). (**b**) Representative fluorescence Western blot showing different catalase protein expressions in TFs and SFs obtained from three technical and three biological replicates. (**c**) Normalized, quantitative catalase protein expression. The densitometric ratio values were previously normalized to total protein from three technical replicates/ passage numbers (original data shown in Supplementary Figure S5) and further normalized to the SF samples. An unpaired two-tailed t-test evaluated differences.

Recombinant catalase abolishes AA- and hydrogen peroxide-induced cell death

A further co-culture experiment was performed to verify whether the AA-induced cell death of the TFs was due to their low catalase activity. Therefore, TFs and SFs were treated with 4 mM AA with and without 500 Units of recombinant catalase. Indeed, the addition of catalase

reversed AA-induced cell death of TFs (Fig. 4a+b, Supplementary Video S5). Finally, it was tested whether the reduced catalase activity of TFs leads to TFs reacting more sensitively to hydrogen peroxide than their neighboring SFs. Therefore, a co-culture experiment was performed on TFs and SFs with and without recombinant catalase in combination with hydrogen peroxide concentrations between 400 and 600 µM (Fig. 4c+d, Supplementary Fig. S4, Supplementary Video S6). Here, the SFs were more robust to hydrogen peroxide exposure than TFs. Again, this effect could be reversed by the addition of recombinant catalase.

Figure 4. Catalase supplementation prevents AA-induced and hydrogen peroxide-induced cell death of TFs *in vitro*.

(**a**) Co-culture of TFs (left) and SFs (right) that were exposed to 4 mM AA without (upper row) or with catalase (lower row) for 24 h. The nuclei were stained with Hoechst, and PI was added to the medium to visualize dead cells. The scale represents 1000 µm. (**b**) The number of dead cells was calculated from the number of PI-positive cells against the total number of Hoechst-positive cells treated without or with 4 mM AA without or with catalase for 24 h in two-hour intervals. The data shows the mean number of dead cells from TFs and SFs obtained from three patients. (**c**) Co-culture of TFs (left) and SFs (right) that were exposed to 500 µM hydrogen peroxide without (upper row) or with catalase (lower row) for 24 h. The nuclei are stained with Hoechst, and dead cells are stained with PI. The scale represents 1000 µm. (**d**) The relative number of dead TFs and SFs was calculated in two-hour intervals from the number of PI-positive cells against the total number of Hoechst-positive cells treated without or with 500 µM hydrogen peroxide without or with catalase for 24 h.

Discussion

In this in vitro study, we partially confirmed the antiproliferative effect of MMC as described by Jampel. However, our live-cell analyses revealed that this effect is driven by MMC's cytotoxicity rather than a specific inhibition of Tenon fibroblast (TF) proliferation. Jampel's original study explored a 24-hour time window, but we found that cytotoxicity from the 5-minute 0.04% MMC exposure only became evident after 36 hours, with the LT_{50} reached at 72 hours (Fig. 1e, Supplementary Video 2). This delayed cytotoxicity raises concerns, as we also observed that MMC preferentially kills scleral fibroblasts (SFs) faster than TFs (Fig. 1e+f). This non-selective action of MMC is consistent with previous reports, which have documented its effects on

scleral, corneal, and conjunctival cells[4, 7], as well as complications such as cystic bleb formation, hypotony, or endophthalmitis findings[4, 8, 9], we aimed to identify compounds that selectively induce cytotoxicity in TFs without harming SFs, thus potentially reducing fibrosis-related complications post-glaucoma surgery.

The rationale for investigating ascorbic acid (AA) and hydrogen peroxide (H_2O_2) was driven by the need for more selective alternatives to MMC to spare adjacent tissues while targeting TFs to mitigate the excessive wound healing that complicates glaucoma surgery.

AA was initially identified during a compound screening in which it unexpectedly accelerated TF death, prompting us to explore its potential further. We hypothesized that AA could induce selective cytotoxicity via oxidative stress, primarily through the Fenton reaction, wherein AA, acting as a pro-oxidant, generates reactive oxygen species (ROS). Our results support this hypothesis, showing that 6–8 mM AA concentrations selectively killed TFs without affecting SFs (Fig. 2a-c, Supplementary Video S3). Interestingly, Jampel's 1990 study also identified AA affecting TFs survival at 1.1 mM[17]. His method involved treating TF cultures derived from human tenon cysts with AA, detaching the cells using trypsin, and re-plating them to quantify the number of reattaching cells. However, we believe this approach, which assesses cell death based on non-attached cells, may not fully capture the subtler cytotoxic effects, as detaching cells with trypsin could interfere with viability measurement. Therefore, our study extends these findings by demonstrating that higher concentrations induce apoptosis rather than merely inhibit proliferation.

The co-culture experiments further demonstrated the selective cytotoxicity of AA on TFs. TFs in monoculture began dying after 5 hours and were entirely dead by 12 hours at 6 mM AA. In co-culture with SFs, TF death was delayed, suggesting that SFs may mitigate but cannot entirely prevent TF death under these conditions (Fig. 2d+e, Supplementary Video S4).

Typically, AA acts as an antioxidant and can help neutralize reactive oxygen. However, in certain situations, AA can support the production of hydroxyl radicals by reducing Fe^{3+} to Fe^{2+}. Fe^{2+}, in combination with hydrogen peroxide, can initiate the Fenton reaction in which hydroxyl

radicals are formed, resulting in irreparable damage to the cells. The cell death caused by the Fenton reaction is also known as ferroptosis and is being investigated as a possible therapeutic approach for killing tumor cells[26, 27]. According to the manufacturer, no iron ions exist in the MEM Medium we used for the cell culture experiments. However, fetal bovine serum (FBS) has been reported to contain ferritin, a protein complex that binds iron ions[28].

The selective effect of AA on TFs may be related to the fact that TFs have a higher hydrogen peroxide level than SFs. The hydrogen peroxide level of the cells is regulated by the enzyme catalase, which neutralizes hydrogen peroxide by cleaving 2x H_2O_2 into 2x H_2O and O_2. Consequently, low catalase expression could increase the cell's hydrogen peroxide concentration, resulting in more hydrogen peroxide forming lethal hydroxyl radicals during the AA-induced Fenton reaction. Therefore, we compared the catalase expression of TFs and SFs from three patients using ICC staining and Western blot quantification. Western blot analysis revealed that catalase expression was reduced by approximately 50 % in TFs compared to SFs (Fig. 3b+c). The selective cytotoxicity of AA on TFs could be entirely abolished by adding recombinant catalase (Fig. 4a+b, Supplementary Video S5). In addition, we show that TFs were more sensitive to hydrogen peroxide in the medium than SFs, presumably due to their reduced catalase expression. Here, too, recombinant catalase could prevent hydrogen peroxide-induced cell death (Fig. 4c+d, Supplementary Video S6). Thus, the reduced catalase expression of TFs represents a weak point that potentially opens up new therapeutic options for IOP-lowering glaucoma surgery.

One limitation of the study is that, for ethical reasons, we could not obtain corneal and conjunctival tissue from the trabeculectomy patients, as these were not removed during surgery. Although we were only able to test the influence of AA on TFs and SFs, the question arises as to what extent AA influences the viability of corneal and conjunctival cells. Treatment with AA or its derivatives has been extensively studied in rabbit eyes, but to our knowledge, there is still too little data for humans. For corneal injuries of the rabbit eye, a ten percent AA solution applied as eye drops showed significantly improved wound healing after one week [29]. Additionally, Chen et al. reported that AA promotes corneal epithelial wound healing in

mice by stimulating the stemness of corneal progenitor cells [30]. Other groups showed that AA eye drop solution could inhibit induced corneal neovascularization in rats and rabbits [31] [32]. The reports mentioned above used a much higher concentration of AA than we used in our study, indicating that the AA concentration we used (8 mM corresponds to a 0.15% solution) is unlikely to affect the viability of corneal or conjunctival cells. Another limitation of this study is that we could not collect clinical data for ethical reasons, as mitomycin C is considered the gold standard for IOP-reducing surgeries in Germany.

In Summary, our data indicate that AA and hydrogen peroxide can prevent excessive wound healing of TFs and, unlike MMC, do not damage SFs significantly *in vitro*. The extent to which both can be applied intraoperatively or postoperatively *in vivo*, for example, during bleb revision, and whether additional iron ions are required *in vivo* to initiate the Fenton reaction by AA will require further investigations.

Acknowledgments

This work was partially funded by the "Zentrales Innovationsprogramm Mittelstand" (ZIM) Grant number ZF4603201AW8 of the Federal Ministry for Economic Affairs and Energy, Germany. The funders had no role in study design, data collection and analysis, decision to publish, or preparation of the manuscript.

References

1. Chen CW, Huang HT, Bair JS, Lee CC. Trabeculectomy with simultaneous topical application of mitomycin-C in refractory glaucoma. Journal of ocular pharmacology. 1990;6(3):175-82. Epub 1990/01/01. doi: 10.1089/jop.1990.6.175. PubMed PMID: 2127056.
2. Jampel HD. Effect of brief exposure to mitomycin C on viability and proliferation of cultured human Tenon's capsule fibroblasts. Ophthalmology. 1992;99(9):1471-6. Epub 1992/09/01. doi: 10.1016/s0161-6420(92)31781-6. PubMed PMID: 1407982.
3. Kim JW, Kim SK, Song IH, Kim IT. Mitomycin C-induced apoptosis in cultured human Tenon's capsule fibroblasts. Korean J Ophthalmol. 1999;13(1):7-15. Epub 2000/04/13. doi: 10.3341/kjo.1999.13.1.7. PubMed PMID: 10761391.

4. Fourman S. Scleritis after glaucoma filtering surgery with mitomycin C. Ophthalmology. 1995;102(10):1569-71. Epub 1995/10/01. doi: 10.1016/s0161-6420(95)30829-9. PubMed PMID: 9097808.
5. Wallin O, Al-ahramy AM, Lundstrom M, Montan P. Endophthalmitis and severe blebitis following trabeculectomy. Epidemiology and risk factors; a single-centre retrospective study. Acta Ophthalmol. 2014;92(5):426-31. Epub 2013/09/12. doi: 10.1111/aos.12257. PubMed PMID: 24020653.
6. Sihota R, Sharma T, Agarwal HC. Intraoperative mitomycin C and the corneal endothelium. Acta Ophthalmol Scand. 1998;76(1):80-2. Epub 1998/04/16. doi: 10.1034/j.1600-0420.1998.760115.x. PubMed PMID: 9541440.
7. Williams TA, Sii F, Chaing M, Murray PI, Shah P. Trabeculectomy with mitomycin C in refractory glaucoma associated with nonnecrotizing anterior scleritis. Ocul Immunol Inflamm. 2009;17(6):420-2. Epub 2009/12/17. doi: 10.3109/09273940903200291. PubMed PMID: 20001263.
8. Kangas TA, Greenfield DS, Flynn HW, Jr., Parrish RK, 2nd, Palmberg P. Delayed-onset endophthalmitis associated with conjunctival filtering blebs. Ophthalmology. 1997;104(5):746-52. Epub 1997/05/01. doi: 10.1016/s0161-6420(97)30238-3. PubMed PMID: 9160018.
9. Gupta S, Wadhwani M, Sehgal V, Sharma S, Dada T. Blebitis with scleral abscess in a case of operated trabeculectomy with mitomycin C and a subcunjunctival ologen implant. Eye (Lond). 2014;28(3):354. Epub 2014/01/11. doi: 10.1038/eye.2013.299. PubMed PMID: 24406406; PubMed Central PMCID: PMCPMC3965825.
10. Khamar M, Bhojwani D, Patel P, Vasavada A. Leftover mitomycin-c sponge causing blebitis. Indian J Ophthalmol. 2019;67(10):1753-5. Epub 2019/09/25. doi: 10.4103/ijo.IJO_1946_18. PubMed PMID: 31546554; PubMed Central PMCID: PMCPMC6786170.
11. Muckley ED, Lehrer RA. Late-onset blebitis/endophthalmitis: incidence and outcomes with mitomycin C. Optom Vis Sci. 2004;81(7):499-504. Epub 2004/07/15. doi: 10.1097/00006324-200407000-00010. PubMed PMID: 15252348.
12. Ayala M. No effects of mitomycin-C in primary trabeculectomies in Sweden. SAGE Open Med. 2018;6:2050312118782262. Epub 2018/07/07. doi: 10.1177/2050312118782262. PubMed PMID: 29977549; PubMed Central PMCID: PMCPMC6024500.
13. Halili A, Kessel L, Subhi Y, Bach-Holm D. Needling after trabeculectomy - does augmentation by anti-metabolites provide better outcomes and is Mitomycin C better than 5-Fluoruracil? A systematic review with network meta-analyses. Acta Ophthalmol. 2020;98(7):643-53. Epub 2020/05/01. doi: 10.1111/aos.14452. PubMed PMID: 32352646.
14. Kitazawa Y, Kawase K, Matsushita H, Minobe M. Trabeculectomy with mitomycin. A comparative study with fluorouracil. Arch Ophthalmol. 1991;109(12):1693-8. Epub 1991/12/01. doi: 10.1001/archopht.1991.01080120077030. PubMed PMID: 1841578.
15. Palmer SS. Mitomycin as adjunct chemotherapy with trabeculectomy. Ophthalmology. 1991;98(3):317-21. Epub 1991/03/01. doi: 10.1016/s0161-6420(91)32293-0. PubMed PMID: 2023752.
16. Liebmann JM, Ritch R, Marmor M, Nunez J, Wolner B. Initial 5-fluorouracil trabeculectomy in uncomplicated glaucoma. Ophthalmology. 1991;98(7):1036-41. Epub 1991/07/01. doi: 10.1016/s0161-6420(91)32180-8. PubMed PMID: 1891210.
17. Jampel HD. Ascorbic acid is cytotoxic to dividing human Tenon's capsule fibroblasts. A possible contributing factor in glaucoma filtration surgery success. Arch Ophthalmol. 1990;108(9):1323-5. Epub 1990/09/01. doi: 10.1001/archopht.1990.01070110139038. PubMed PMID: 2095758.
18. Przekora A, Zarnowski T, Ginalska G. A simple and effective protocol for fast isolation of human Tenon's fibroblasts from a single trabeculectomy biopsy - a comparison of cell behaviour in different culture media. Cellular & molecular biology letters. 2017;22:5. Epub 2017/05/26. doi: 10.1186/s11658-017-0034-4. PubMed PMID: 28536636; PubMed Central PMCID: PMC5415760.
19. Seko Y, Azuma N, Takahashi Y, Makino H, Morito T, Muneta T, et al. Human sclera maintains common characteristics with cartilage throughout evolution. PLoS One. 2008;3(11):e3709. Epub 2008/11/13. doi: 10.1371/journal.pone.0003709. PubMed PMID: 19002264; PubMed Central PMCID: PMCPMC2579486.
20. Hu X, Binter M, Hufendiek K, Tode J, Framme C, Fuchs H. MiR-302d inhibits TGFB-induced EMT and promotes MET in primary human RPE cells. PLoS One. 2022;17(11):e0278158. Epub 2022/11/29.

doi: 10.1371/journal.pone.0278158. PubMed PMID: 36441751; PubMed Central PMCID: PMCPMC9704570.
21. Fuchs H, Jahn K, Hu X, Meister R, Binter M, Framme C. Breaking a Dogma: High-Throughput Live-Cell Imaging in Real-Time with Hoechst 33342. Adv Healthc Mater. 2023;12(20):e2300230. Epub 2023/03/20. doi: 10.1002/adhm.202300230. PubMed PMID: 36934382.
22. Stahnke T, Kowtharapu BS, Stachs O, Schmitz KP, Wurm J, Wree A, et al. Suppression of TGF-beta pathway by pirfenidone decreases extracellular matrix deposition in ocular fibroblasts in vitro. PLoS One. 2017;12(2):e0172592. Epub 2017/02/24. doi: 10.1371/journal.pone.0172592. PubMed PMID: 28231275; PubMed Central PMCID: PMCPMC5322922.
23. Gajda-Derylo B, Stahnke T, Struckmann S, Warsow G, Birke K, Birke MT, et al. Comparison of cytokine/chemokine levels in aqueous humor of primary open-angle glaucoma patients with positive or negative outcome following trabeculectomy. Bioscience reports. 2019;39(5). Epub 2019/04/11. doi: 10.1042/BSR20181894. PubMed PMID: 30967499; PubMed Central PMCID: PMC6499456.
24. Meyer-ter-Vehn T, Sieprath S, Katzenberger B, Gebhardt S, Grehn F, Schlunck G. Contractility as a prerequisite for TGF-beta-induced myofibroblast transdifferentiation in human tenon fibroblasts. Invest Ophthalmol Vis Sci. 2006;47(11):4895-904. Epub 2006/10/27. doi: 10.1167/iovs.06-0118. PubMed PMID: 17065504.
25. Hu X, Meister R, Tode J, Framme C, Fuchs H. Long-term in vitro monitoring of AAV-transduction efficiencies in real-time with Hoechst 33342. PLoS One. 2024;19(3):e0298173. Epub 2024/03/01. doi: 10.1371/journal.pone.0298173. PubMed PMID: 38427668; PubMed Central PMCID: PMCPMC10906819.
26. Jasim KA, Gesquiere AJ. Ultrastable and Biofunctionalizable Conjugated Polymer Nanoparticles with Encapsulated Iron for Ferroptosis Assisted Chemodynamic Therapy. Mol Pharm. 2019;16(12):4852-66. Epub 2019/10/16. doi: 10.1021/acs.molpharmaceut.9b00737. PubMed PMID: 31613630.
27. Shen Z, Song J, Yung BC, Zhou Z, Wu A, Chen X. Emerging Strategies of Cancer Therapy Based on Ferroptosis. Adv Mater. 2018;30(12):e1704007. Epub 2018/01/23. doi: 10.1002/adma.201704007. PubMed PMID: 29356212; PubMed Central PMCID: PMCPMC6377162.
28. Rhyner K, Taetle R, Bering H, To D. Transferrin receptor regulation is coupled to intracellular ferritin in proliferating and differentiating HL60 leukemia cells. J Cell Physiol. 1985;125(3):608-12. Epub 1985/12/01. doi: 10.1002/jcp.1041250333. PubMed PMID: 2999167.
29. Gujral GS, Askari SN, Ahmad S, Zakir SM, Saluja K. Topical vitamin C, vitamin E, and acetylcysteine as corneal wound healing agents: A comparative study. Indian J Ophthalmol. 2020;68(12):2935-9. Epub 2020/11/25. doi: 10.4103/ijo.IJO_1463_20. PubMed PMID: 33229673; PubMed Central PMCID: PMCPMC7856962.
30. Chen J, Lan J, Liu D, Backman LJ, Zhang W, Zhou Q, et al. Ascorbic Acid Promotes the Stemness of Corneal Epithelial Stem/Progenitor Cells and Accelerates Epithelial Wound Healing in the Cornea. Stem Cells Transl Med. 2017;6(5):1356-65. Epub 2017/03/10. doi: 10.1002/sctm.16-0441. PubMed PMID: 28276172; PubMed Central PMCID: PMCPMC5442716.
31. Peyman GA, Kivilcim M, Morales AM, DellaCroce JT, Conway MD. Inhibition of corneal angiogenesis by ascorbic acid in the rat model. Graefes Arch Clin Exp Ophthalmol. 2007;245(10):1461-7. Epub 2007/02/24. doi: 10.1007/s00417-007-0542-4. PubMed PMID: 17318569.
32. Lee MY, Chung SK. Treatment of corneal neovascularization by topical application of ascorbic acid in the rabbit model. Cornea. 2012;31(10):1165-9. Epub 2012/07/27. doi: 10.1097/ICO.0b013e318241433b. PubMed PMID: 22832865.

Supplementary Figure Captions

Supplementary Figure S1. Phase-contrast images and ICC-Staining for ACTA2, VIM, FN1, and F-Actin on human TFs and SFs.

TFs (upper row) and SFs (lower row) at passage # 3 were cultured for 1 week and ICC-stained for ACTA2, VIM, or Phalloidin-Red to visualize F-Actin. Nuclei were stained with DAPI, and the scale bar represents 50 µm.

Supplementary Figure S2. ICC-staining of human TFs and their ability to induce TGFB2-mediated endothelial-to-mesenchymal transition (EndoTM).

Representative ICC results from human TFs treated without (upper row) or with recombinant TGFB2 (lower row) for one week. Cells were immunostained for ACTA2, FN1, VIM, or Phalloidin-Red to visualize F-Actin. The scale bar represents 50 µm.

Supplementary Figure S3. Hoechst 33342 does not impact the AA-induced cell death.

Cytotoxicity analysis of TFs (left) and SFs (right) exposed to 6 mM AA in combination with or without 28 nM Hoechst 33342. The y-axis shows the total number of PI-positive nuclei.

Supplementary Figure S4. Catalase prevents hydrogen peroxide-induced cell death in both SFs and TFs.

The relative numbers of dead TFs and SFs were calculated in two-hour intervals by dividing the number of PI-positive cells by the number of Hoechst-positive cells treated without or with 400 µM (left) or 600 µM (right) hydrogen peroxide without or with 500 Units Catalase for 24 h.

Supplementary Figure S5

A-C) Raw WB images used for Fig. 3 b and WB quantification

Supplementary Video Captions

Supplementary Video S1. Phase contrast time-lapse image videos of TFs between days 6 and 7 after initial culture were treated intraoperatively without or with 0.02 % MMC for 3 minutes. The scale bar presents 1000 µm.

Supplementary Video S2. Time-lapse image videos showing co-cultured TFs and SFs treated *in vitro* either with 0.04 % MMC for 5 minutes (left video) or permanently exposed to 0.001 % MMC for 90 hours (right video). Hoechst 33342 and PI were added to visualize the nuclei and numbers of dead cells. PI-negative nuclei are masked with yellow circles, whereas PI-positive nuclei are highlighted in red circles. The scale bar presents 1000 µm.

Supplementary Video S3.

Time-lapse video showing mono-cultured TFs and SFs treated without or with 6 mM AA for 36 h. In addition to the phase-contrast recording, the Hoechst-stained nuclei are shown. The scale bar presents 1000 µm.

Supplementary Video S4.

Time-lapse videos showing co-cultured TFs and SFs exposed to 6 mM AA for 24 h. In addition to the phase-contrast recording, fluorescence recordings of the Hoechst-stained nuclei, the PI-stained nuclei, and the merged Hoechst and PI recordings are shown. The scale bar presents 1000 µm.

Supplementary Video S5. Time-lapse videos showing co-cultured TFs and SFs exposed to 4 mM AA (left video) or combined with 500 U recombinant catalase (right video) for 24 h. Hoechst 33342 and PI were added to visualize the nuclei and numbers of dead cells. PI-negative nuclei are masked with yellow circles, whereas PI-positive nuclei are highlighted in red circles. The scale bar presents 1000 µm.

Supplementary Video S6. Time-lapse videos showing co-cultured TFs and SFs exposed to 500 µM hydrogen peroxide (left video) or combined with 500 U recombinant catalase (right video) for 24 h. Hoechst 33342 and PI were added to visualize the nuclei and numbers of dead cells. PI-negative nuclei are masked with yellow circles, whereas PI-positive nuclei are highlighted in red circles. The scale bar presents 1000 µm.

Figure 1

Click here to access/download;Figure;Figure 1.tif

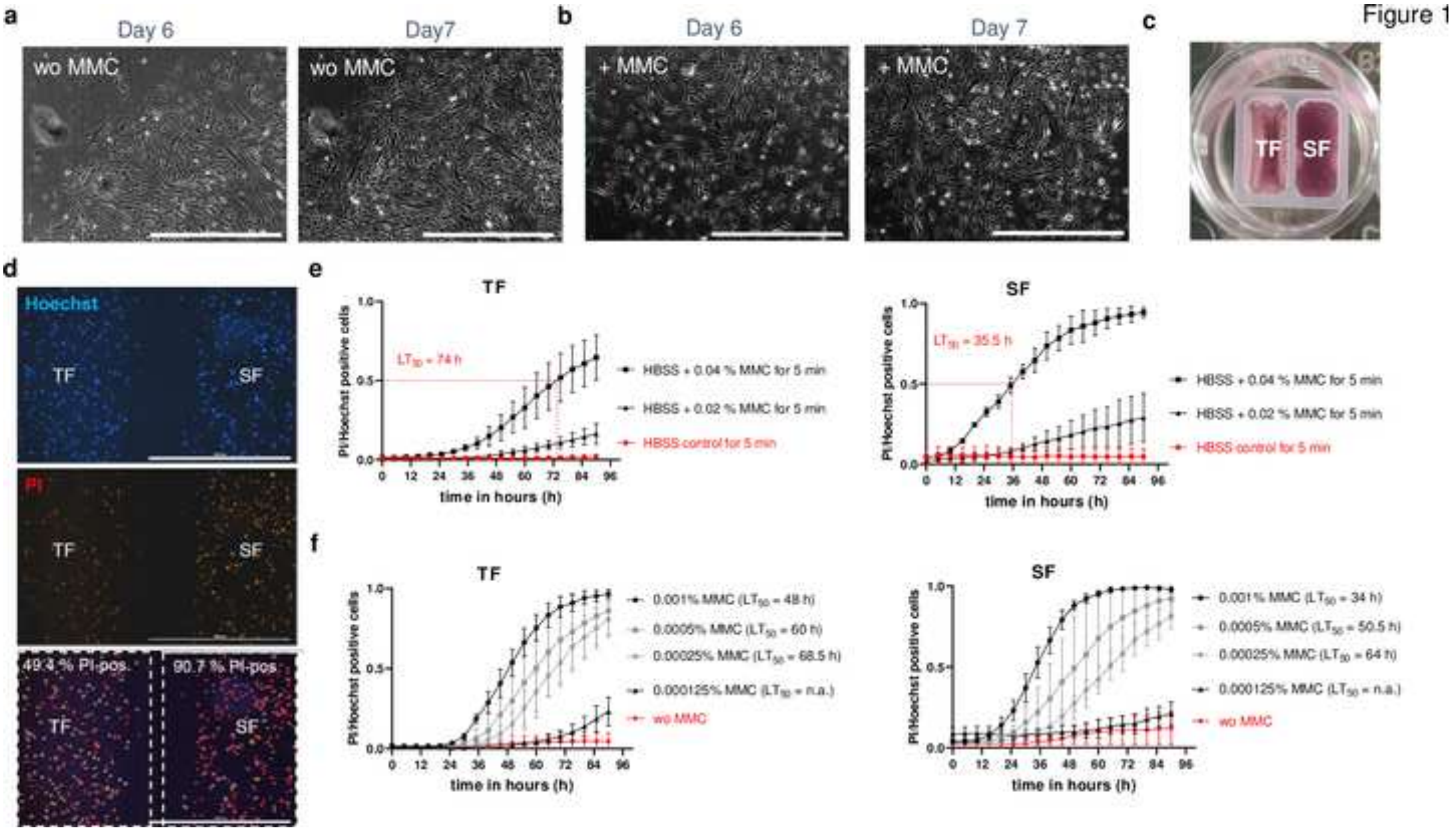

Figure 2

Click here to access/download;Figure;Figure 2.tif

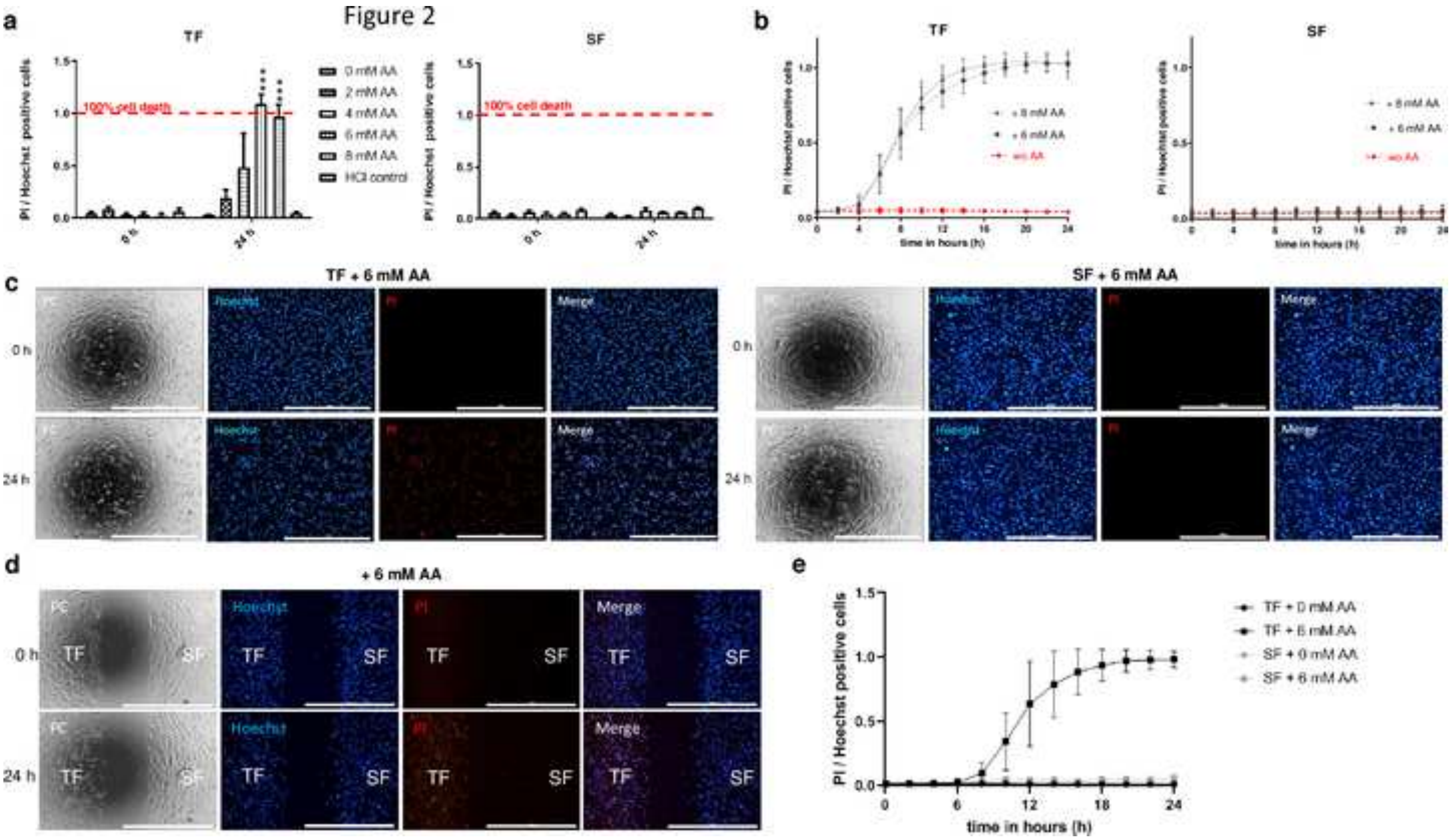

Figure 4

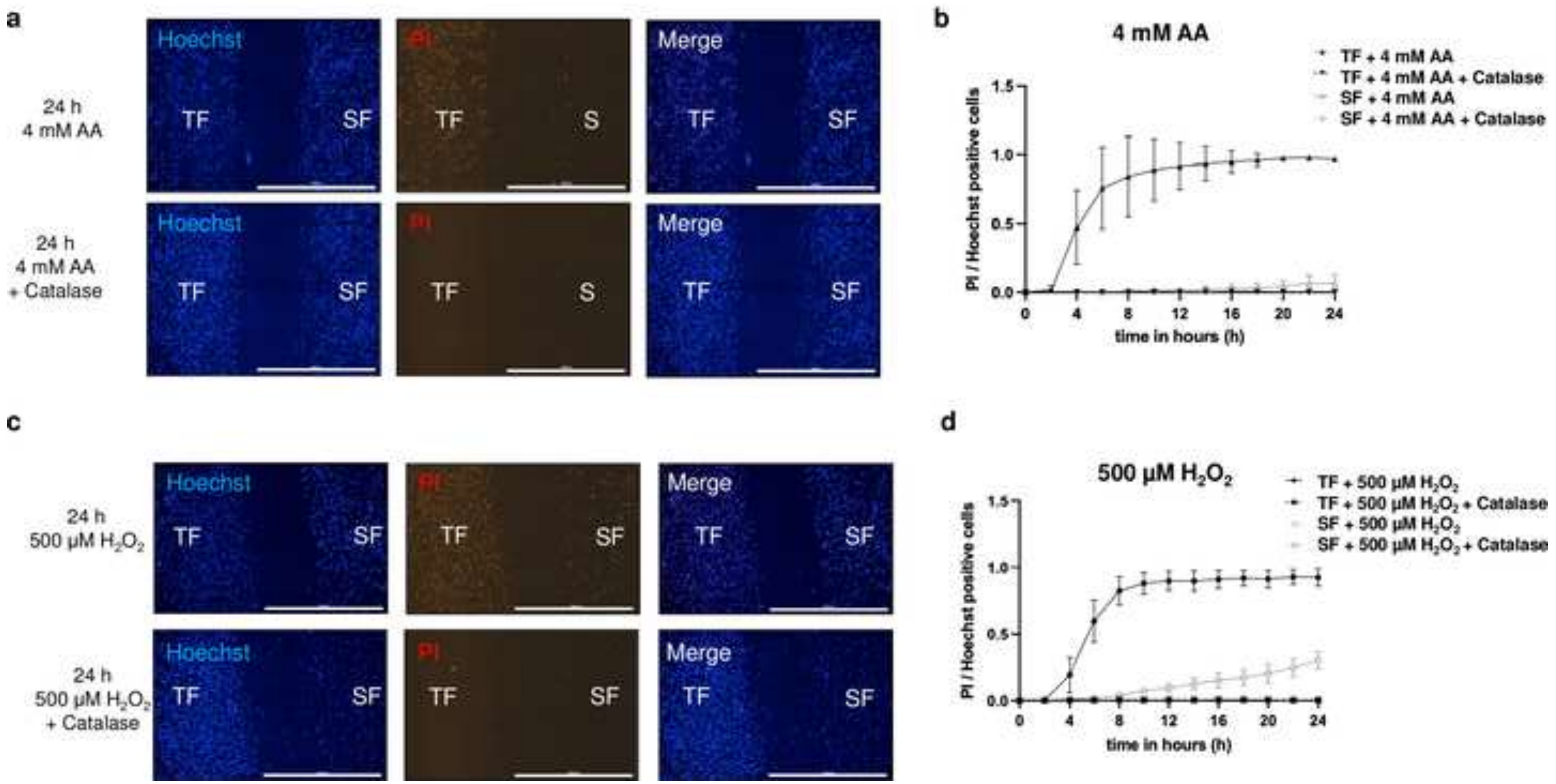
a
24 h
4 mM AA
24 h
4 mM AA
+ Catalase
Hoechst
PI
Merge
TF
SF
S
b
4 mM AA
TF + 4 mM AA
TF + 4 mM AA + Catalase
SF + 4 mM AA
SF + 4 mM AA + Catalase
PI / Hoechst positive cells
time in hours (h)
c
24 h
500 µM H_2O_2
24 h
500 µM H_2O_2
+ Catalase
d
500 µM H_2O_2
TF + 500 µM H_2O_2
TF + 500 µM H_2O_2 + Catalase
SF + 500 µM H_2O_2
SF + 500 µM H_2O_2 + Catalase

Figure 3

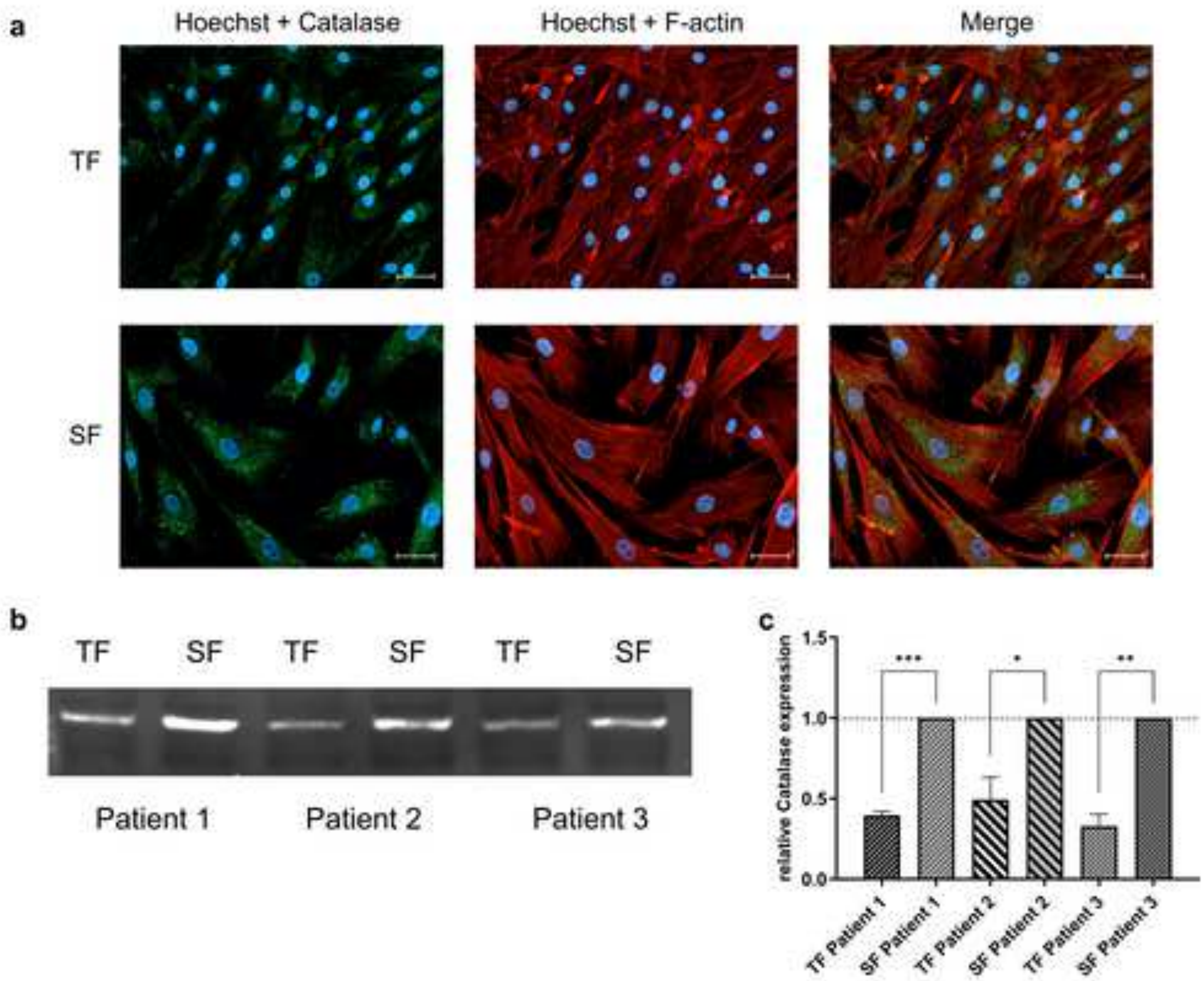
a
Hoechst + Catalase
Hoechst + F-actin
Merge
TF
SF
b
TF
SF
TF
SF
TF
SF
Patient 1
Patient 2
Patient 3
c
relative Catalase expression
1.5
1.0
0.5
0.0
***
*
**
TF Patient 1
SF Patient 1
TF Patient 2
SF Patient 2
TF Patient 3
SF Patient 3

Supplementary Figures S1-S5

Click here to access/download
Supporting Information
Supplementary Figures SF1-5.pdf

Supplementary Video S1

Click here to access/download
Supporting Information
Video S1.mp4

Supplementary Video S2

Click here to access/download

Supporting Information

Video S2.mp4

Supplementary Video S3

Click here to access/download
Supporting Information
Video S3.mp4

Supplementary Video S4

Click here to access/download
Supporting Information
Video S4.mp4

Supplementary Video S5

Click here to access/download
Supporting Information
Video S5.mp4

Supplementary Video S6

Click here to access/download
Supporting Information
Video S6.mp4

S1_raw_images

Click here to access/download
Supporting Information
S1_raw_images.pdf

Raw Data of Fig. 1-4

Click here to access/download
Supporting Information
PlosoneDataofFigures.xlsx

Selective ~~Cytotoxicity~~Toxicity of Ascorbic Acid and Hydrogen Peroxide on ~~Primary~~ Human Tenon ~~Fibroblasts~~Cells without ~~harming~~Harming Scleral ~~fibroblasts *in vitro*~~Cells *In Vitro*: A Possible Alternative to Non-Selective Mitomycin C?

Short Title: **Selective Cytotoxicity of Ascorbic Acid on Primary Human Tenon Fibroblasts**

Heiko Fuchs[1, #, *], Xiaonan Hu[1, *], Roland Meister[1], Yuqing Huang[1], Martin C. Bartram[1], Amelie Pielen[1,2], Bernd Junker[1,2] Jan Tode[1], Carsten Framme[1]

[1] Department of Ophthalmology, University Eye Hospital, Hannover Medical School, Carl-Neuberg-Straße 1, 30625 Hannover, Germany

[2] Maximilians-Augenklinik, Erlenstegenstraße 30, 90491 Nürnberg, Germany

[*] These authors contributed equally to this work.

[#] Correspondence: fuchs.heiko@mh-hannover.de

Formatted: English (United Kingdom)

Formatted: Justified, Line spacing: Double

Formatted: English (United Kingdom)

Formatted: English (United Kingdom)

Formatted: English (United Kingdom)

Formatted: English (United Kingdom)

Formatted: English (United Kingdom)

~~Abstract~~

Background: ~~Blindness worldwide~~Glaucoma, a leading cause of blindness, is ~~commonly caused~~ often driven by ~~glaucoma, wherein the optic nerve can be irreparably damaged by excessive~~ elevated intraocular pressure (IOP~~).~~), which damages the optic nerve. Transscleral filtration surgery ~~is crucial in severe glaucoma for lowering elevated~~reduces IOP~~. Postoperatively,~~ but is frequently complicated by excessive wound healing ~~by tenon~~from Tenon fibroblasts (TFs~~) can impede~~), impeding aqueous humor absorption. Mitomycin C (MMC~~) is intraoperatively~~), used for over 30 years in ophthalmic surgeries, inhibits TF proliferation but carries significant side effects, including hypotony, blebitis, and endophthalmitis, due to its non-selective cytotoxicity. MMC's inability to entirely prevent ~~scar formation, but it does not~~ fibrosis increases surgical failure risk, often necessitating further interventions like bleb needling. This study investigates whether ascorbic acid (AA) and hydrogen peroxide (H_2O_2) can selectively target TFs~~, resulting in cell damage to neighboring cell types, such as~~ without damaging scleral fibroblasts (SFs~~).~~) *in vitro*, using MMC ~~is insufficient to prevent a scarring reaction, leading to~~ as a ~~high failure rate of IOP-reducing drainage procedures. This study aimed to identify substances selectively harmful to human primary TFs without damaging SFs~~benchmark.

Methods: Primary human TFs and SFs ~~cultures~~ were ~~obtained from tenon and sclera tissue of patients who underwent~~cultured from patient trabeculectomy~~. Different~~ tissues. Cells were treated with various concentrations of MMC, ~~ascorbic acid (AA) or hydrogen peroxide were added to the cells and live-cell analysis was performed using a live-~~AA, or H_2O_2. Cytotoxic effects were analyzed via live-cell imaging. Immunocytochemistry and Western Blot assessed catalase expression in both cell types and recombinant catalase was used to validate its protective effect against AA- and H_2O_2-induced cell death.

Results: Short-term exposure (5 min) to 0.02%-0.04% MMC or long-term exposure to 0.00025%-0.001% MMC caused cytotoxicity in TFs and SFs, with SFs dying significantly earlier. In contrast, AA (6–8 mM) selectively induced cell ~~microscope. Additionally, immunocytochemical staining and Western Blot analysis were performed~~death in TFs without harming SFs. H_2O_2 also showed selective cytotoxicity towards TFs. Lower catalase expression in TFs compared to SFs was determined via Western blot and immunocytochemistry, highlighting a mechanism for this selective effect. Recombinant catalase neutralized the cytotoxic effects of AA and H_2O_2 on TFs.

~~**Results:** 5-minute exposure to 0.04% MMC resulted in the death of both cell types, with SFs affected earlier. In contrast to MMC, 6-8 mM Ascorbic acid (AA) selectively induced TFs death~~

Formatted: Font: Not Bold

Formatted: Normal, Space Before: Auto, After: Auto, Line spacing: 1.5 lines

Formatted: Font: Not Bold

Formatted: Normal, Space Before: Auto, After: Auto, Line spacing: 1.5 lines

Formatted: Font: Not Bold

~~within 24 hours without harming SFs. Hydrogen peroxide showed a similar effect. Lower catalase expression in TFs in comparison to SFs was exploited. The addition of recombinant catalase neutralized AA or hydrogen peroxide-induced TFs death.~~

~~**Conclusions:** Both AA and hydrogen peroxide might potentially enhance the success rates of transscleral filtration surgery by selectively targeting TFs.~~

Conclusions: Unlike MMC, Ascorbic acid and hydrogen peroxide exhibit selective cytotoxicity towards Tenon fibroblasts, which may provide a safer, more targeted approach for preventing fibrosis in glaucoma surgery. Additional *in vivo* studies are needed to explore the clinical applicability of these findings.

Keywords

glaucoma, fibrosis, ascorbic acid, tenon fibroblasts, sclera fibroblasts, catalase, Mitomycin C, live-cell imaging, hydrogen peroxide

Formatted: Default Paragraph Font, Font: Arial, 12 pt, Bold, English (United Kingdom)

Formatted: Justified

Introduction

In glaucoma implant surgery and classic trabeculectomy, the aqueous humor is directed through a surgically prepared channel into a sub-conjunctival located reservoir called a filtering bleb. The conjunctiva absorbs the aqueous humor, which lowers the elevated intraocular pressure (IOP) to prevent irreparable damage to the optic nerve. However, undesirable excessive wound healing processes, such as fibrosis, occur in numerous cases. Either a fibrotic tenon cyst forms, creating an absorption barrier at the site of the reservoir, or the surgically created fistula is blocked by proliferating cells that produce an extracellular matrix. Both wound-healing processes lead to insufficient absorption of aqueous humor and increased

IOP. Wound healing originates from cells of the tenon layer, which spatially separates the sclera from the conjunctiva. To prevent fibrosis of tenon fibroblasts (TFs), parts of the tenon layer are often surgically removed. In addition, mitomycin C (MMC) is applied to inhibit the proliferation of TFs.

Chen and co-workers were the first to propose the intraoperative application of MMC in trabeculectomy with a supposedly poor outcome in an 8-year follow-up study in 1990[1]. They reported that MMC can prevent excessive wound healing and thus reduce intraocular pressure more efficiently for a more extended period. Jampel confirmed in 1992 that a 5-minute exposure of 0.01 % and 0.04 % MMC is sufficient to inhibit the proliferation of TFs within 24 h *in vitro [2]*. Intraoperatively, a sponge soaked with 0.02 - 0.04% MMC is inserted into the prepared conjunctival pocket above the sclera for 2 to 5 min and then rinsed with a saline solution to wash out excess MMC. MMC is a cytostatic drug that penetrates the cells and inhibits DNA replication, thus preventing cell proliferation and inducing apoptosis[3]. Severe DNA damage may lead to DNA mutation and cell death. Therefore, MMC is classified as a carcinogenic substance, and its medical application is controversial. Another disadvantage of MMC is that it does not selectively kill TFs and may induce corneal, scleral, and conjunctival cell apoptosis [4-7].

Severe side effects have been reported, such as postoperative wound healing disorders or the formation of an avascular filtering bleb, hypotony, endophthalmitis, and blebitis [8-11]. Another retrospective study raises doubts that a 2 to 5-minute application of MMC significantly prevents a postoperative tenon cyst formation [12]. It is often reported that an excessive wound-healing process occurs despite the intraoperative application of these cytostatic drugs. A postoperative follow-up treatment, such as a bleb revision, might be necessary. In a bleb needling procedure, MMC or 5-Fluorouracil is often administered subconjunctivally as an adjuvant to restore the function of a fibrosing bleb[9]. The benefit of this postoperative administration of these cytostatic drugs is not undisputed [13]. Despite the above-described controversial effects, MMC has been used in ophthalmic surgery for over 30 years, probably due to a lack of alternatives [1, 14-16].

Also, about 30 years ago, in 1990, Jampel reported that 1.1 mM ascorbic acid (AA), a concentration found in aqueous humor, reduced the plating efficiency of cell suspensions of human Tenon's capsule fibroblasts by an average of 40% within 24 h *in vitro* [17]. Furthermore, he showed that 1.1 mM AA addition reduced cell number by approximately 14% in confluent tenon cultures. He speculated that if AA has a similar effect *in vivo*, it could lead to incomplete wound healing and thus contribute to successful glaucoma filtration surgery.

~~We~~In contrast to MMC, we aimed to identify compounds that prevent excessive wound healing of TFs without harming scleral fibroblasts (SFs~~) in contrast to MMC.~~). Therefore, tenon and scleral tissues from six human patients who underwent trabeculectomy were isolated to establish primary cell cultures. These primary cell cultures were further used for drug screening experiments. Here, we investigated the impact of AA and hydrogen peroxide on TFs and SFs *in vitro*.

Material and Methods

Cell Culture

Tenon and scleral tissues were obtained from six patients who underwent trabeculectomy. The procedures were carried out following the tenets of the Declaration of Helsinki, following the guidelines and regulations of and with the approval of the ethics committee of Hannover Medical School (File number Nr. 8220_BO_K_2018). Patient samples were collected from August 2019 to September 2020. Written informed consent was obtained from all subjects. ~~Primary cells~~Some Tenon tissues were excised before the intraoperative application of MMC, while others were removed afterward. Primary TFs were obtained from tenon tissue according to a modified protocol of Przekora et al.[18]. Briefly, tenon tissue was cultured as a tissue attachment explant in Minimum Essential Medium Eagle (Sigma #M8042, Saint Louis, MI, USA) containing 5 % FBS (PAN #P40-39500, Aidenbach, Germany), GlutaMAX™ (Thermo Fisher #35050061, Waltham, MA, USA), Penicillin/Streptomycin (Gibco #3505-038),

supplemented with 5 ng ml^{-1} fibroblast growth factor 2 (FGF-2) (Peprotech #AF-100-18B) and 5 µg ml^{-1} insulin (Thermo Fisher #12585014) in a 12-well. An outgrowth of TFs from the tissue could be observed approximately three to four days later. After the initial cell culture, cells were cultivated in the same medium without FGF-2 or insulin. TFs were split at around 90% confluence and were cultured until passage number ten. ~~Cells~~

Sclera tissue was obtained during the trabeculectomy procedure when a small incision was made in the sclera to alleviate intraocular pressure. SFs obtained from scleral tissue ~~followed~~were expanded following the protocol of Seko et al., except that scleral tissue was not cut into small pieces [19]. For SFs, Minimum Essential Medium Eagle (Sigma #M8042) supplemented with 5% FBS, GlutaMAX™ (Thermo Fisher #35050061), and Penicillin/Streptomycin (Gibco #3505-038) was used for cultivation. Cell outgrowth was observed after approximately one week. Cell passages were performed at about 90 % confluence. TFs and SFs between passages two and eight were used for the following experiments.

Transforming growth factor-beta 2 treatment

For TGFB2 treatment, $2x10^4$ TFs were seeded in a 24-well plate with 0.5 mL complete medium containing 5% FBS. After 24 h, the medium was exchanged with the medium that contained 2% FBS and 10 ng ml^{-1} human recombinant TGFB2 (Peprotech #100-35B). TFs were cultured for one week, with the medium replaced after four days.

Immunocytochemistry staining

For ICC staining, $2x10^4$ TFs~~,~~ and SFs were seeded on a 13 mm diameter slide coverslip (Glaswarenfabrik Karl Hecht #41001113, Bavaria, Germany) into each well of a 24-well plate with 0.5 mL complete medium. Before fixation, cells were washed twice with PBS (Carl Roth #1058.1, Baden-Württemberg, Germany). Cells were immediately fixed with 4%

paraformaldehyde (Carl Roth #P087.6) for 20 min at room temperature (RT) and washed twice with PBS for 5 min. Blocking was performed for 1 h at RT with a blocking solution containing 5% goat serum (Millipore #S26-100ml, Burlington, MA, USA), 0.02% Tween®20 (Sigma #P9416) and 0.01% Triton™X-100 (Sigma #X100) in PBS. ACTA2 rabbit mAb (Cell Signaling #19245, Danvers, MA, USA), VIM rabbit mAb (Cell Signaling #5741), FN1 rabbit mAb (Cell Signaling #26836), and Catalase rabbit mAb (Cell Signaling #12980S) were diluted 1:1000 in the blocking solution and cells were incubated overnight at 4°C. Cells were then washed twice with PBS for 10 min at RT and incubated with either 1:500 diluted AlexaFluor™546 goat anti-rabbit (Invitrogen #A11035) or AlexaFluor™488 goat anti-rabbit (Invitrogen #A11034) and 4 µl ml^{-1} rhodamine-phalloidin (Invitrogen #R415) in PBST (0.1% Tween®20 in PBS) for 2 h at RT. After three washes in PBS for 10 min at RT, the coverslips were mounted upside down on a slide with Roti®-Mount FluorCare DAPI (Carl Roth #HP20.1). Phase-contrast and fluorescence images were recorded with an Observer Z.1 microscope (Carl Zeiss, Baden-Württemberg, Germany) using the ZEN-Blue analysis software (Carl Zeiss).

Western blot

$10x10^4$ TFs and SFs were cultured in 6-well plates with 2 mL complete medium. Sample preparation, Electrophoresis, blotting, and antibody incubation were performed as previously described by our group[20]. Cells were washed twice with PBS and lysed with 1x Laemmli Sample Buffer (Bio-Rad #1610747, Hercules, CA, USA) containing 1x protease inhibitor cocktail (Cell signaling #5871S). The lysates were mixed with 2-Mercaptoethanol (Sigma-Aldrich #60-24-2), denatured at 70˚C for 10 min, centrifuged at 4000 rpm for 2 min, and stored at -20˚C. The samples were loaded on Mini-Protean TGX Stain-Free Gels (Bio-Rad #4568094). Briefly, after 80–100 V electrophoresis, the resolved proteins were transferred to an ethanol-activated Mini-size LF PVDF membrane (Bio-Rad #10026934) with Trans-Blot®Turbo™ Transfer System at 1.3 A, 25 V for 7 min. The PVDF membrane was blocked with 5% milk powder (Carl Roth #T145.2) in 1X Tris-buffered saline (TBS) at RT for 1 h,

followed by incubation with 1:1000 diluted primary Catalase rabbit mAb (Cell signaling #12980S) overnight at 4˚C. After incubation with 1:4000 diluted goat anti-rabbit secondary antibody StarBright™ Blue 700 (Bio-Rad #12004162) at RT for 1 h, the fluorescent signal was detected with ChemiDoc MP Imaging System (Bio-Rad) at a measurement wavelength of 660–720 nm. ImageLab 6 software (Bio-Rad) was used for normalization and quantification.

Mitomycin C ~~treatment~~ (MMC) Treatment

For the intraoperative application of Mitomycin C (MMC), a concentration of 0.04% MMC was prepared and applied using a sponge. The MMC was left in contact with the tissue for three minutes to allow for adequate absorption. Following the application, the area was washed twice with 10 ml of Hanks' Balanced Salt Solution (HBSS) to remove any residual MMC.

For the co-culture experiments, ~~1x10^4~~ Tenon fibroblasts (TFs) and scleral fibroblasts (SFs) were seeded ~~into each part of~~in 48-well plates using a 2-well IBIDI-cell culture insert (IBIDI #80209, Bavaria, Germany~~) in 48-well plates.~~), with 1 x 10^4 cells per well for each cell type. After 24 ~~h later~~hours, the inserts were ~~taken out with medium change. Both cells were treated with or without~~removed, and fresh medium containing either 0.02 % or 0.04 % MMC (Sigma #M4287) in Hank's Balanced Salt Solution (HBSS, Carl Roth #9119.1) was applied to the wells for 5 ~~min~~minutes to mimic ~~the~~ intraoperative MMC ~~application. Fresh~~exposure. Subsequently, the medium was replaced with fresh medium containing 28 nM Hoechst 33342 (Thermo Fisher #H1399) and 2 µg/ml~~$^{-1}$~~ PI (Sigma #P4170) ~~was changed subsequently. For the permanent exposure of MMC, a concentration of 0 – 0.001 % MMC was added with Hoechst and PI of the exact dosage as above. Live~~for live-cell imaging ~~was performed afterwards~~to monitor cytotoxic effects. In addition, for long-term exposure, MMC was directly added to the medium at concentrations of 0.000125%, 0.00025%, or 0.001%, and the cells were exposed and monitored for 90 ~~h~~hours to simulate postoperative conditions.

Formatted: Left, Space Before: Auto, After: Auto, Line spacing: single

Formatted: Font: Not Bold

Formatted: Space Before: Auto, After: Auto

Formatted: English (United Kingdom)

Ascorbic acid treatment

In ~~the~~ monoculture experiments, ~~$3x10^4$~~ $3x10^4$ Tenon fibroblasts (TFs) or scleral fibroblasts (SFs) were seeded in 48-well plates with 250 µL ~~media~~of medium containing 2% FBS, 28 nM Hoechst 33342~~,~~ (Thermo Fisher #H1399), and 2 µg~~ ~~/ml~~$^{-1}$~~ Propidium Iodide (PI~~ and~~) (Sigma-Aldrich #P4170). They were treated with different concentrations of Ascorbic Acid (AA) (Sigma #A0278) ~~concentrations (~~ranging from 0 ~~-~~to 8 mM~~). With the addition of~~. When 8 mM AA was added, the pH of the medium dropped from 7.7 to 6.8. ~~For the~~To rule out the possibility that this pH drop caused cytotoxicity, an HCl control~~,~~ with a pH of 6.8 was included by adding 6.7 µL of 1N HCl (Carl Roth #4625.1) per mL ~~was added to achieve a similar pH of 6.8.~~of medium. The medium with HCl was ~~later~~ sterilized ~~by~~ using a 0.22 µm PES syringe filter (Carl Roth #P668.1). Live-cell imaging was then performed for 24 ~~h~~hours.

~~In the co-culture experiments, $1x10^4$ TFs and SFs were seeded as previously. 24 h later, the inserts were taken out, and a fresh medium containing Hoechst, PI, and AA was used with or without 500U ml^{-1} catalase solution (Sigma #SLCJ5891). Live-cell imaging was then performed for 24 h.~~

Hydrogen peroxide treatment

$1x10^4$ TFs and SFs were seeded and co-cultured as previously. 24 h later, the inserts were taken out, and the medium was changed to fresh medium containing 28 nM Hoechst 33342, 2 µg ml^{-1} PI, different hydrogen peroxide (Sigma #216763-100ML) concentrations (400-600 µM) together with or without 500U ml^{-1} catalase solution~~.~~ (Sigma #SLCJ5891). Live-cell imaging was performed for 24 h.

Rekombinant Catalase Treatment

In the co-culture experiments, $1x10^4$ TFs and SFs were seeded as described above. 24 h later, the inserts were taken out, and a fresh medium containing Hoechst, PI, and AA or Hydrogen

Formatted: English (United Kingdom)

peroxide was used with or without 400, 500, or 600 U ml^{-1} catalase solution. Live-cell imaging was then performed for 24 h.

Live-cell imaging

Live cell imaging experiments were performed using the BioTek® Lionheart™ FX Automated Microscope (Agilent Technologies, Santa Clara, CA, USA). The experiment setup and subsequent analysis were performed as previously described by our group using the Gen5 Image Prime 3.05 software[21][21]. The 48-well culture plate was placed in the humidity chamber with CO_2 levels set to 5% and temperature to 37°C. Imaging was carried out in the phase-contrast channel using the 4X PL FL phase objective and laser autofocus. In addition to the phase-contrast images, the DAPI fluorescence filter set was used to detect Hoechst-stained nuclei, and the RFP fluorescence filter was used to detect PI-positive nuclei. Images were recorded for 48 h at 20-min intervals. The acquisition parameters for the phase contrast channel were set to 10 "LED",100 "Integration time" and 4.8 "gain". For DAPI, the values were set to 6 "LED",71 "Integration time" and 24 "gain". For RFP, the values were set to 10 "LED",120 "integration time" and 21 "gain". The relatively high gain settings were used to keep the exposure time as short as possible to minimize photobleaching during live-cell imaging.

Live-cell imaging analysis

The number of Hoechst-positive objects per image was determined using the cell count option of Gen5 analysis software, as previously described[21][21]. The analysis tool was briefly used, and the channel "DAPI 377, 4747" was selected under "Primary mask and Count". The "Threshold" was set to 2000, the background to "Dark", and the options "Split touching objects" and "Fill holes in masks" were selected. In addition, under "Advanced Detection Options", a "Background flattening" with a "Rolling Ball diameter" of 30 µm, an "Image smooth strength" of 2 "Cycles of 3x3 average filter", and "Evaluate background on 5 % of lowest pixels" were

selected. Under "Object selection", a "Min. object size" of 10 µm and a "Max. object size" of 70 µm were specified, and the option "Include primary edge objects" was selected. For the non-co-culture experiments, the option "Analyse entire image" was selected. For the co-culture experiments, the "Plug" tool was used to place a rectangular window on the left side of the image to count the TFs and a rectangular window on the right side to calculate the SFs. Both windows had a size of 750 x 1500 µm. The number of PI-positive objects was calculated using the analysis tool to count dead cells. Therefore, under "Primary mask and Count", the channel "RFP 531, 593" was selected, a threshold of the positive PI signal was set, and the parameters previously described in the DAPI channel were used for further analysis. The data was further analyzed using Microsoft Excel 2010. The number of PI-positive cells was divided by the number of Hoechst-positive nuclei to determine the percentage of dead cells per area for each time point. The mean and standard deviation of biological replicates were calculated from these values with Graph Pad Prism 9.

Statistical analysis

All experiments were performed in at least three independent replicates, and data were analyzed using Microsoft Excel 2010 and GraphPad Prism 9. ~~An unpaired two-tailed t-test evaluated differences. A *p*-value < 0.05 was considered significantly different~~A one-way ANOVA was used to determine statistical significance and compare catalase expression between Tenon fibroblasts (TFs) and Scleral fibroblasts (SFs). Post hoc analysis used Tukey's HSD test to compare the groups. Data are presented as mean ± standard deviation (SD) with significance thresholds set at ******p < 0.01, and *******p < 0.001**.**

Formatted: Font: Bold

Results

Effects of MMC on TFs *ex vivo* */in vitro*

Tenon and scleral explants were collected from six trabeculectomy patients. The explants were transferred into a cell culture medium to allow TF and SF to outgrow, according to a modified protocol of Przekora and Seko[18, 19]. Phase contrast images and immunocytochemistry (ICC) staining for vimentin (VIM), actin alpha 2 (ACTA2), and F-actin were obtained from the isolated TFs and SFs (Supplementary Fig. S1). Furthermore, it was tested whether TFs transform into myofibroblasts after one week of transforming growth factor beta 2 (TGFB2) exposure, as reported previously by other groups [22-24]. Indeed, TGFB2-treated TFs show a more widespread distribution of VIM within the cytoplasm, an increase of ACTA2, an increase of the extracellular matrix protein fibronectin 1 (FN1), and a resemble of the F-Actin, compared to the untreated TFs (Supplementary Fig. S2). Remarkably, two out of six tenon tissue samples were treated intraoperatively with 0.~~02~~04 % MMC for 3 to 5 minutes before the tissue was removed~~, whereas~~. In contrast, the other four tenon tissues were removed before MMC was applied. However, an outgrowth of proliferating TFs was observed in all tenon tissue samples after 5-7 days, regardless of whether they were treated intraoperatively with MMC (Fig. 1a+b, Supplementary Video S1). ~~All TFs could be~~We successfully expanded and ~~cultured till~~culture TFs up to and beyond passage ~~number ten. Therefore, an~~10, regardless of intraoperative MMC treatment. This observation suggests that MMC did not exert a lasting antiproliferative effect ~~on the tenon tissue by intraoperatively 0.02 % MMC treatment for 5 minutes could not be observed, at least~~in ex vivo~~.~~ conditions.

Formatted: Font: Not Italic

Figure 1. Cytotoxic effects of MMC on human TFs and SFs *in vitro*.
Representative phase-contrast images showing TF cells at days 6 and 7 after trabeculectomy and initial tenon tissue culture, respectively, treated intraoperatively without (**a**) or with MMC (**b**). The scale is 1000 µm. (**c**) Co-culture inserts were used to culture TFs and SFs separately within one well of a 48-well plate. (**d**) Representative fluorescent pictures of co-cultured TF and SF cells that were treated for 5 min with 0.04 % MMC at time point 74 h. During the experiment, cells were stained with Hoechst 33342 (upper row) and PI (middle row) to visualize dead cells. The right and left sections of the image (highlighted by the two white dashed rectangles) were analyzed to quantify the dead TF and SF cells separately. The total number of cells was determined by gating the Hoechst-stained nuclei for the analysis. This mask subsequently identifies PI-positive nuclei by setting a threshold in the red channel. In the lower row, PI-positive nuclei are highlighted in red circles, whereas PI-negative nuclei are highlighted in yellow circles. (**e**) Cytotoxicity analysis for 90 h of PI- and Hoechst-stained co-cultured TFs (left diagram) and SFs (right diagram) after treatment with 0.02 % and 0.04 % MMC in HBSS for 5 minutes. HBBS alone for 5 min was used as a control. After MMC treatment and before live-

cell imaging, cells were cultured in regular growth media with Hoechst and PI. For the five-minute 0.04 % MMC treatment, the LT50, i.e., the time 50 % of the cells have died, is also given. (**f**) Cytotoxicity analysis of PI- and Hoechst-stained co-cultured TFs (left diagram) and SFs (right diagram) that were permanently exposed without or with 0.000125 %, 0.00025 %, 0.005 % or 0.001% MMC for 90 hours. Where possible, the LT50 for different MMC concentrations was specified here.

Short-term and long-term exposure to MMC on TFs and SF *in vitro*

Since TFs are in close contact with SFs in the eye, TFs and SFs were cultivated together in a 48-well using a co-culture insert (Fig. 1c). To test for a possible cytotoxic effect of MMC in the co-cell culture model, Hoechst 33342 was added to label the cell nuclei, and propidium iodide (PI) to label dead cells. ~~The Hoechst concentration of 28 nM and the acquisition parameters used for live-cell imaging were neither cytotoxic nor phototoxic, as described in a previous study [21].~~The Hoechst concentration of 28 nM and the acquisition parameters used for live-cell imaging were neither cytotoxic nor phototoxic, as described in previous studies[21, 25]. To mimic the intraoperative MMC application *in vitro*, TFs and SFs were treated for 5 minutes without or with 0.02 % or 0.04 % MMC. After a subsequent medium change with the addition of Hoechst and PI, the number of dead cells was determined over 90 hours using a live-cell imager and subsequent image analysis. To determine the percentage of dead cells, the number of PI-positive nuclei was divided by the number of Hoechst-stained nuclei for TFs and SFs separately (Fig. 1d), and the calculated lethal time 50 (LT_{50}) revealed that with a five-minute 0.04% MMC application, half of the TFs had died after 74 h. In contrast, the LT_{50} for SFs was only ~ 35.5 h (Fig. 1e).

In a further experiment, both cell types were permanently exposed to MMC for 90 hours but at lower concentrations between 0.001 % and 0.000125 %. The LT_{50} values for each MMC concentration confirmed the tendency for SFs to die faster from MMC than TFs (Fig. 1f). Representative time-lapse videos of the co-cultures exposed to either 0.04 % MMC for 5 min or 0.001 % MMC permanently are shown in the Supplementary Video S2.

Impact of AA on TFs and SFs *in vitro*

Since Jampel demonstrated in 1990 that 1 mM AA inhibits the viability of TFs in vitro, we wanted to test the effect of AA on both cell types. For this approach, confluent TFs or SFs were exposed to different AA concentrations between 0 and 8 mM for 24 h. Notably, the pH value of the 8 mM AA treatment condition decreased from 7.7 to 6.8. To exclude effects caused by pH decrease, an additional control was included with a medium adjusted to pH 6.8 using 1N hydrochloric acid (HCl). The percentage of dead TFs (left panel) and SFs (right panel) within 24 hours was determined by dividing the number of PI-positive cells by the total number of Hoechst-positive cells (Fig. 2A). Data reveals that AA concentrations of 6 and 8 mM resulted in complete cell death of TFs within 24 h. No significant cell death was observed in the untreated cells or HCl control. Furthermore, no significant cytotoxic effect of AA on SFs was observed (Supplementary Video 3). No AA-induced cytotoxicity was observed in sclera cells after the 24 h window. Therefore, subsequent LCI analyses were performed in the 24-hour time window.

Figure 2. Low concentrations of AA selectively kill human TFs without harming SFs *in vitro*.

(**a**) The two bar diagrams show the percentage of dead TFs (left side) and SFs (right side), which were exposed to 0, 2, 4, 6, and 8 mM AA or HCl control at time point zero and after 24 h. (**b**) Cell toxicity analysis of monocultured TFs (right diagram) and SFs (left diagram) that were treated without or with 6 mM or 8 mM AA for 24 h. For data collection, live cell analysis was performed three times on TFs and SFs obtained from six trabeculectomy patients. (**c**) Representative phase-contrast and Hoechst and PI fluorescence images of monocultured TFs (left panel) and SFs (right panel) with 6 mM AA for 0 h (upper row) and 24 h (lower row). The scale bar represents 1000 µm. (**d**) Representative phase-contrast and Hoechst and PI fluorescence images of co-cultured TFs and SFs treated with 6 mM AA for 0 h (upper row) and 24 h (lower row). The scale bar represents 1000 µm. (**e**) Cell toxicity analysis of co-cultured TFs and SFs treated without or with 6 mM AA for 24 h.

To evaluate the possibility that AA-induced cell death was caused by nuclear staining with Hoechst, we treated TFs with 0 and 6 mM AA in combination with and without Hoechst in the medium. Live-cell imaging analysis showed that Hoechst alone did not cause cytotoxicity in the TFs within 24 hours, while the TFs treated with 6 mM AA died after about 12 hours without

any synergistic effect caused by Hoechst staining (Supplementary Figure S3). To further exclude whether AA-induced cell death of TFs was possibly patient-specific or coincidental, we tested AA on TFs and SFs of six trabeculectomy patient samples. Therefore, we treated TFs and SFs from six trabeculectomy patients in monoculture experiments without or with 6 mM and 8 mM AA. In all 6 patient samples, AA concentrations between 6 and 8 mM were cytotoxic for TFs but harmless for SFs (Fig. 2b+c).

Since the tenon cell layer is located directly above the sclera *in vivo*, we wanted to investigate whether signaling molecules or free radicals might be released by AA-induced death of TFs, which could impair the viability of the SFs. For this purpose, we co-cultured TFs and SFs in one well of a two-chamber cell culture set and incubated them for 24 h. The insert was removed, spatially separating both cell types, and treated with or without 6 mM AA with PI and Hoechst. In co-cultures, we observed that TFs ultimately died within 24 hours in the presence of 6 mM AA, whereas no significant cytotoxicity was observed for the SFs (Fig. 2d+e, Supplementary Video 4).

Catalase expression in TFs and SFs

Next, we wanted to investigate how AA selectively attacks the TFs without damaging the SFs. Numerous studies have shown that AA sometimes initiates the $Fe^{3+} \rightarrow Fe^{2+}$ redox cycle in the Fenton reaction. Fe^{2+} further reacts with hydrogen peroxide, ~~leading to an increase of~~increasing toxic hydroxyl radicals that can irreparably damage the cells. Consequently, the concentration of hydroxyl radicals formed depends on the amount of free Fe^{2+} and the concentration of hydrogen peroxide. The hydrogen peroxide concentration is regulated by endogenous catalase expression. Immunofluorescence staining revealed that both cell types express catalase within the cytoplasm (Fig. 3a). However, a Western blot analysis of TFs and SFs from three patients showed that the catalase protein levels are significantly reduced in TFs compared to SFs (Fig. 3b+c).

Figure 3. Unveiling a potential weak spot: TFs have lower catalase expression than SFs.

(**a**) Representative ICC-staining showing catalase expression (green), F-Actin (red), and DAPI (blue) of TFs (upper row) and SFs (lower row). (**b**) Representative fluorescence Western blot showing different catalase protein expressions in TFs and SFs obtained from three technical and three biological replicates. (**c**) Normalized, quantitative catalase protein expression. The densitometric ratio values were previously normalized to total protein from three technical replicates/ passage numbers (original data shown in Supplementary Figure S5) and further normalized to the SF samples. An unpaired two-tailed t-test evaluated differences.

Recombinant catalase abolishes AA- and hydrogen peroxide-induced cell death

A further co-culture experiment was performed to verify whether the AA-induced cell death of the TFs was due to their low catalase activity. Therefore, TFs and SFs were treated with 4 mM AA with and without 500 Units of recombinant catalase. Indeed, the addition of catalase reversed AA-induced cell death of TFs (Fig. 4a+b, Supplementary Video S5). Finally, it was tested whether the reduced catalase activity of TFs leads to TFs reacting more sensitively to hydrogen peroxide than their neighboring SFs. Therefore, a co-culture experiment was performed on TFs and SFs with and without recombinant catalase in combination with hydrogen peroxide concentrations between 400 and 600 µM (Fig. 4c+d, Supplementary Fig. S4, Supplementary Video S6). Here, the SFs were more robust to hydrogen peroxide exposure than TFs. Again, this effect could be reversed by the addition of recombinant catalase.

Figure 4. Catalase supplementation prevents AA-induced and hydrogen peroxide-induced cell death of TFs *in vitro*.

(**a**) Co-culture of TFs (left) and SFs (right) that were exposed to 4 mM AA without (upper row) or with catalase (lower row) for 24 h. The nuclei were stained with Hoechst, and PI was added to the medium to visualize dead cells. The scale represents 1000 µm. (**b**) The number of dead cells was calculated from the number of PI-positive cells against the total number of Hoechst-positive cells treated without or with 4 mM AA without or with catalase for 24 h in two-hour intervals. The data shows the mean number of dead cells from TFs and SFs obtained from three patients. (**c**) Co-culture of TFs (left) and SFs (right) that were exposed to 500 µM hydrogen peroxide without (upper row) or with catalase (lower row) for 24 h. The nuclei are stained with Hoechst, and dead cells are stained with PI. The scale represents 1000 µm. (**d**) The relative number of dead TFs and SFs was calculated in two-hour intervals from the number of PI-positive cells against the total number of Hoechst-positive cells treated without or with 500 µM hydrogen peroxide without or with catalase for 24 h.

Discussion

In this in vitro study, we partially confirmed the antiproliferative effect of MMC as described by Jampel was partially confirmed.. However, our live--cell analyses indicaterevealed that the antiproliferative this effect is due to thedriven by MMC's cytotoxicity rather than a specific inhibition of MMC (Fig.1). Jampel investigated a short time window of 24 hours after the initial MMC exposure. At this Tenon fibroblast (TF) proliferation. Jampel's original study explored a 24-hour time point, we were also unable to detect any window, but we found that cytotoxicity (Fig.1e). Cytotoxicity offrom the 5-minute 0.04 % MMC application manifestedexposure only became evident after 36 h, andhours, with the LT_{50} was reached afterat 72 h (hours (Fig. 1e, Supplementary Video 2). Of concern was This delayed cytotoxicity raises concerns, as we also observed that MMC preferentially killed the SFskills scleral fibroblasts (SFs) faster than the TFs, at least *in vitro*, as shown by the determined LT_{50} values (Fig. (Fig. 1e+f).

Several studies report that MMC induces apoptosis of potentially fibrosing tenon cells and negatively affects scleralThis non-selective action of MMC is consistent with previous reports, which have documented its effects on scleral, corneal, and conjunctival cell viability cells[4, 7]. MMC has been reported to cause , as well as complications such as cystic bleb formation, hypotony, corneal erosions, breakdown of the conjunctiva, or a leaking bleb. Severe complications such as scleritis or endophthalmitis may occurfindings[4, 8, 9].

Therefore, our study , we aimed to identify substancescompounds that, unlike MMC, can selectively damage TFs to prevent excess wound healing. During our research, we came across Jampel's 1990 publication in which he found a high AA concentration of 1.1 mM in aqueous humor and his *in vitro* study investigating AA's effects on human TFs. For the study, Jampel seeded TFs, allowed them to grow to confluence, treated them with different concentrations of AA for 25 hours, detached them with trypsin, and plated them again to determine the number of living cells. Jampel determined a lethal dosage of 50 percent (LD_{50}) concentration of 0.7 mM for 25 hours. Jampel's results prompted us to investigate the influence

Formatted: Font: Not Italic

Formatted: English (United Kingdom)

Formatted: English (United Kingdom)

Formatted: English (United Kingdom)

of AA on TFs and SFs in more detail.induce cytotoxicity in TFs without harming SFs, thus potentially reducing fibrosis-related complications post-glaucoma surgery.

Formatted: Font: Not Bold

The monoculture experiments reveal that a 6 to 8 mM AA concentration can induce apoptosis of confluent human TFs within 24 h. In contrast, SFs show no signs of cell death (Fig. 2a-c, Supplementary Video S3). Although we present live cell analysis only from a 24-hour time window, we could not observe cell death of the SFs even after three days of AA treatment. However, our data cannot confirm the LD_{50} dose of 0.7 mM AA determined by Jampel. The lowest concentration of 2 mM AA we tested, only approx. 20 % of the TFs were dead after 24 h. After 24 h, approx. 50 % of the TFs were PI-positive at an AA concentration of 4 mM (Fig. 2a).

The co-culture experiment, in which we treated both TFs and SFs in one well with 6 mM AA, illustrates even more clearly the selective cytotoxicity of AA on TFs *in vitro* (Fig. 2d+e, Supplementary Video S4). Notably, TFs in monoculture experiments started to die after about 5 h. They were utterly dead after 12 h at a concentration of 6 mM AA (Fig. 2b). Conversely, TFs only started to die after 9 h in the co-culture experiment. They were utterly dead after 20 h (Fig. 2e). This time delay reveals that the SFs present in the co-culture experiment may reduce but do not prevent AA-induced cell death of TFs.

The rationale for investigating ascorbic acid (AA) and hydrogen peroxide (H_2O_2) was driven by the need for more selective alternatives to MMC to spare adjacent tissues while targeting TFs to mitigate the excessive wound healing that complicates glaucoma surgery.

AA was initially identified during a compound screening in which it unexpectedly accelerated TF death, prompting us to explore its potential further. We hypothesized that AA could induce selective cytotoxicity via oxidative stress, primarily through the Fenton reaction, wherein AA, acting as a pro-oxidant, generates reactive oxygen species (ROS). Our results support this hypothesis, showing that 6–8 mM AA concentrations selectively killed TFs without affecting SFs (Fig. 2a-c, Supplementary Video S3). Interestingly, Jampel's 1990 study also identified

AA affecting TFs survival at 1.1 mM[17]. His method involved treating TF cultures derived from human tenon cysts with AA, detaching the cells using trypsin, and re-plating them to quantify the number of reattaching cells. However, we believe this approach, which assesses cell death based on non-attached cells, may not fully capture the subtler cytotoxic effects, as detaching cells with trypsin could interfere with viability measurement. Therefore, our study extends these findings by demonstrating that higher concentrations induce apoptosis rather than merely inhibit proliferation.

The co-culture experiments further demonstrated the selective cytotoxicity of AA on TFs. TFs in monoculture began dying after 5 hours and were entirely dead by 12 hours at 6 mM AA. In co-culture with SFs, TF death was delayed, suggesting that SFs may mitigate but cannot entirely prevent TF death under these conditions (Fig. 2d+e, Supplementary Video S4).

Typically, AA acts as an antioxidant and can help neutralize reactive oxygen. However, in certain situations, AA can support the production of hydroxyl radicals by reducing Fe^{3+} to Fe^{2+}. Fe^{2+}, in combination with hydrogen peroxide, can initiate the Fenton reaction in which hydroxyl radicals are formed, resulting in irreparable damage to the cells. The cell death caused by the Fenton reaction is also known as ferroptosis and is being investigated as a possible therapeutic approach for killing tumor cells[~~25, ~~26, 27]. According to the manufacturer, no iron ions exist in the MEM Medium we used for the cell culture experiments. However, fetal bovine serum (FBS) has been reported to contain ferritin, a protein complex that binds iron ions~~[27].~~[28].

The selective effect of AA on TFs may be related to the fact that TFs have a higher hydrogen peroxide level than SFs. The hydrogen peroxide level of the cells is regulated by the enzyme catalase, which neutralizes hydrogen peroxide by cleaving 2x H_2O_2 into 2x H_2O and O_2. Consequently, low catalase expression could increase the cell's hydrogen peroxide concentration, resulting in more hydrogen peroxide forming lethal hydroxyl radicals during the AA-induced Fenton reaction. Therefore, we compared the catalase expression of TFs and SFs from three patients using ICC staining and Western blot quantification. Western blot analysis revealed that catalase expression was reduced by approximately 50 % in TFs compared to SFs (Fig. 3b+c). The selective cytotoxicity of AA on TFs could be entirely abolished by adding

~~500 U~~ recombinant catalase (Fig. 4a+b, Supplementary Video S5). In addition, we show that TFs were more sensitive to hydrogen peroxide in the medium than SFs, presumably due to their reduced catalase expression. Here, too, recombinant catalase could ~~also abolish~~prevent hydrogen peroxide-induced cell death (Fig. 4c+d, Supplementary Video S6). Thus, the reduced catalase expression of TFs represents a weak point that potentially opens up new therapeutic options for IOP-lowering glaucoma surgery.

One limitation of the study is that, for ethical reasons, we could not obtain corneal and conjunctival tissue from the trabeculectomy patients, as these were not removed during surgery. Although we were only able to test the influence of AA on TFs and SFs, the question arises as to what extent AA influences the viability of corneal and conjunctival cells. Treatment with AA or its derivatives has been extensively studied in rabbit eyes, but to our knowledge, there is still too little data for humans. For corneal injuries of the rabbit eye, a ten percent AA solution applied as eye drops showed significantly improved wound healing after one week ~~[28].~~[29]. Additionally, Chen et al. reported that AA promotes corneal epithelial wound healing in mice by stimulating the stemness of corneal progenitor cells [~~29~~30]. Other groups showed that AA eye drop solution could inhibit induced corneal neovascularization in rats and rabbits [~~30~~31] ~~[31].~~[32]. The reports mentioned above used a much higher concentration of AA than we used in our study, indicating that the AA concentration we used (8 mM corresponds to a 0.15% solution) is unlikely to affect the viability of corneal or conjunctival cells. Another limitation of this study is that we could not collect clinical data for ethical reasons, as mitomycin C is considered the gold standard for IOP-reducing surgeries in Germany.

In Summary, our data indicate that AA and hydrogen peroxide can prevent excessive wound healing of TFs and, unlike MMC, do not damage SFs significantly *in vitro*. The extent to which both can be applied intraoperatively or postoperatively *in vivo*, for example, during bleb revision, and whether additional iron ions are required *in vivo* to initiate the Fenton reaction by AA will require further investigations.

Acknowledgments

This work was partially funded by the "Zentrales Innovationsprogramm Mittelstand" (ZIM) Grant number ZF4603201AW8 of the Federal Ministry for Economic Affairs and Energy, Germany. The funders had no role in study design, data collection and analysis, decision to publish, or preparation of the manuscript.

References

1. Chen CW, Huang HT, Bair JS, Lee CC. Trabeculectomy with simultaneous topical application of mitomycin-C in refractory glaucoma. Journal of ocular pharmacology. 1990;6(3):175-82. Epub 1990/01/01. doi: 10.1089/jop.1990.6.175. PubMed PMID: 2127056.
2. Jampel HD. Effect of brief exposure to mitomycin C on viability and proliferation of cultured human Tenon's capsule fibroblasts. Ophthalmology. 1992;99(9):1471-6. Epub 1992/09/01. doi: 10.1016/s0161-6420(92)31781-6. PubMed PMID: 1407982.
3. Kim JW, Kim SK, Song IH, Kim IT. Mitomycin C-induced apoptosis in cultured human Tenon's capsule fibroblasts. Korean J Ophthalmol. 1999;13(1):7-15. Epub 2000/04/13. doi: 10.3341/kjo.1999.13.1.7. PubMed PMID: 10761391.
4. Fourman S. Scleritis after glaucoma filtering surgery with mitomycin C. Ophthalmology. 1995;102(10):1569-71. Epub 1995/10/01. doi: 10.1016/s0161-6420(95)30829-9. PubMed PMID: 9097808.
5. Wallin O, Al-ahramy AM, Lundstrom M, Montan P. Endophthalmitis and severe blebitis following trabeculectomy. Epidemiology and risk factors; a single-centre retrospective study. Acta Ophthalmol. 2014;92(5):426-31. Epub 2013/09/12. doi: 10.1111/aos.12257. PubMed PMID: 24020653.
6. Sihota R, Sharma T, Agarwal HC. Intraoperative mitomycin C and the corneal endothelium. Acta Ophthalmol Scand. 1998;76(1):80-2. Epub 1998/04/16. doi: 10.1034/j.1600-0420.1998.760115.x. PubMed PMID: 9541440.
7. Williams TA, Sii F, Chaing M, Murray PI, Shah P. Trabeculectomy with mitomycin C in refractory glaucoma associated with nonnecrotizing anterior scleritis. Ocul Immunol Inflamm. 2009;17(6):420-2. Epub 2009/12/17. doi: 10.3109/09273940903200291. PubMed PMID: 20001263.
8. Kangas TA, Greenfield DS, Flynn HW, Jr., Parrish RK, 2nd, Palmberg P. Delayed-onset endophthalmitis associated with conjunctival filtering blebs. Ophthalmology. 1997;104(5):746-52. Epub 1997/05/01. doi: 10.1016/s0161-6420(97)30238-3. PubMed PMID: 9160018.
9. Gupta S, Wadhwani M, Sehgal V, Sharma S, Dada T. Blebitis with scleral abscess in a case of operated trabeculectomy with mitomycin C and a subcunjunctival ologen implant. Eye (Lond). 2014;28(3):354. Epub 2014/01/11. doi: 10.1038/eye.2013.299. PubMed PMID: 24406406; PubMed Central PMCID: PMCPMC3965825.
10. Khamar M, Bhojwani D, Patel P, Vasavada A. Leftover mitomycin-c sponge causing blebitis. Indian J Ophthalmol. 2019;67(10):1753-5. Epub 2019/09/25. doi: 10.4103/ijo.IJO_1946_18. PubMed PMID: 31546554; PubMed Central PMCID: PMCPMC6786170.
11. Muckley ED, Lehrer RA. Late-onset blebitis/endophthalmitis: incidence and outcomes with mitomycin C. Optom Vis Sci. 2004;81(7):499-504. Epub 2004/07/15. doi: 10.1097/00006324-200407000-00010. PubMed PMID: 15252348.
12. Ayala M. No effects of mitomycin-C in primary trabeculectomies in Sweden. SAGE Open Med. 2018;6:2050312118782262. Epub 2018/07/07. doi: 10.1177/2050312118782262. PubMed PMID: 29977549; PubMed Central PMCID: PMCPMC6024500.

13. Halili A, Kessel L, Subhi Y, Bach-Holm D. Needling after trabeculectomy - does augmentation by anti-metabolites provide better outcomes and is Mitomycin C better than 5-Fluoruracil? A systematic review with network meta-analyses. Acta Ophthalmol. 2020;98(7):643-53. Epub 2020/05/01. doi: 10.1111/aos.14452. PubMed PMID: 32352646.

14. Kitazawa Y, Kawase K, Matsushita H, Minobe M. Trabeculectomy with mitomycin. A comparative study with fluorouracil. Arch Ophthalmol. 1991;109(12):1693-8. Epub 1991/12/01. doi: 10.1001/archopht.1991.01080120077030. PubMed PMID: 1841578.

15. Palmer SS. Mitomycin as adjunct chemotherapy with trabeculectomy. Ophthalmology. 1991;98(3):317-21. Epub 1991/03/01. doi: 10.1016/s0161-6420(91)32293-0. PubMed PMID: 2023752.

16. Liebmann JM, Ritch R, Marmor M, Nunez J, Wolner B. Initial 5-fluorouracil trabeculectomy in uncomplicated glaucoma. Ophthalmology. 1991;98(7):1036-41. Epub 1991/07/01. doi: 10.1016/s0161-6420(91)32180-8. PubMed PMID: 1891210.

17. Jampel HD. Ascorbic acid is cytotoxic to dividing human Tenon's capsule fibroblasts. A possible contributing factor in glaucoma filtration surgery success. Arch Ophthalmol. 1990;108(9):1323-5. Epub 1990/09/01. doi: 10.1001/archopht.1990.01070110139038. PubMed PMID: 2095758.

18. Przekora A, Zarnowski T, Ginalska G. A simple and effective protocol for fast isolation of human Tenon's fibroblasts from a single trabeculectomy biopsy - a comparison of cell behaviour in different culture media. Cellular & molecular biology letters. 2017;22:5. Epub 2017/05/26. doi: 10.1186/s11658-017-0034-4. PubMed PMID: 28536636; PubMed Central PMCID: PMC5415760.

19. Seko Y, Azuma N, Takahashi Y, Makino H, Morito T, Muneta T, et al. Human sclera maintains common characteristics with cartilage throughout evolution. PLoS One. 2008;3(11):e3709. Epub 2008/11/13. doi: 10.1371/journal.pone.0003709. PubMed PMID: 19002264; PubMed Central PMCID: PMCPMC2579486.

20. Hu X, Binter M, Hufendiek K, Tode J, Framme C, Fuchs H. MiR-302d inhibits TGFB-induced EMT and promotes MET in primary human RPE cells. PLoS One. 2022;17(11):e0278158. Epub 2022/11/29. doi: 10.1371/journal.pone.0278158. PubMed PMID: 36441751; PubMed Central PMCID: PMCPMC9704570.

21. Fuchs H, Jahn K, Hu X, Meister R, Binter M, Framme C. Breaking a Dogma: High-Throughput Live-Cell Imaging in Real-Time with Hoechst 33342. Adv Healthc Mater. 2023;12(20):e2300230. Epub 2023/03/20. doi: 10.1002/adhm.202300230. PubMed PMID: 36934382.

22. Stahnke T, Kowtharapu BS, Stachs O, Schmitz KP, Wurm J, Wree A, et al. Suppression of TGF-beta pathway by pirfenidone decreases extracellular matrix deposition in ocular fibroblasts in vitro. PLoS One. 2017;12(2):e0172592. Epub 2017/02/24. doi: 10.1371/journal.pone.0172592. PubMed PMID: 28231275; PubMed Central PMCID: PMCPMC5322922.

23. Gajda-Derylo B, Stahnke T, Struckmann S, Warsow G, Birke K, Birke MT, et al. Comparison of cytokine/chemokine levels in aqueous humor of primary open-angle glaucoma patients with positive or negative outcome following trabeculectomy. Bioscience reports. 2019;39(5). Epub 2019/04/11. doi: 10.1042/BSR20181894. PubMed PMID: 30967499; PubMed Central PMCID: PMC6499456.

24. Meyer-ter-Vehn T, Sieprath S, Katzenberger B, Gebhardt S, Grehn F, Schlunck G. Contractility as a prerequisite for TGF-beta-induced myofibroblast transdifferentiation in human tenon fibroblasts. Invest Ophthalmol Vis Sci. 2006;47(11):4895-904. Epub 2006/10/27. doi: 10.1167/iovs.06-0118. PubMed PMID: 17065504.

25. Jasim KA, Gesquiere AJ. Ultrastable and Biofunctionalizable Conjugated Polymer Nanoparticles with Encapsulated Iron for Ferroptosis Assisted Chemodynamic Therapy. Mol Pharm. 2019;16(12):4852-66. Epub 2019/10/16. doi: 10.1021/acs.molpharmaceut.9b00737. PubMed PMID: 31613630Hu X, Meister R, Tode J, Framme C, Fuchs H. Long-term in vitro monitoring of AAV-transduction efficiencies in real-time with Hoechst 33342. PLoS One. 2024;19(3):e0298173. Epub 2024/03/01. doi: 10.1371/journal.pone.0298173. PubMed PMID: 38427668; PubMed Central PMCID: PMCPMC10906819.

2626. Jasim KA, Gesquiere AJ. Ultrastable and Biofunctionalizable Conjugated Polymer Nanoparticles with Encapsulated Iron for Ferroptosis Assisted Chemodynamic Therapy. Mol Pharm. 2019;16(12):4852-66. Epub 2019/10/16. doi: 10.1021/acs.molpharmaceut.9b00737. PubMed PMID: 31613630.

27. Shen Z, Song J, Yung BC, Zhou Z, Wu A, Chen X. Emerging Strategies of Cancer Therapy Based on Ferroptosis. Adv Mater. 2018;30(12):e1704007. Epub 2018/01/23. doi: 10.1002/adma.201704007. PubMed PMID: 29356212; PubMed Central PMCID: PMCPMC6377162.

~~27~~28. Rhyner K, Taetle R, Bering H, To D. Transferrin receptor regulation is coupled to intracellular ferritin in proliferating and differentiating HL60 leukemia cells. J Cell Physiol. 1985;125(3):608-12. Epub 1985/12/01. doi: 10.1002/jcp.1041250333. PubMed PMID: 2999167.

~~28~~29. Gujral GS, Askari SN, Ahmad S, Zakir SM, Saluja K. Topical vitamin C, vitamin E, and acetylcysteine as corneal wound healing agents: A comparative study. Indian J Ophthalmol. 2020;68(12):2935-9. Epub 2020/11/25. doi: 10.4103/ijo.IJO_1463_20. PubMed PMID: 33229673; PubMed Central PMCID: PMCPMC7856962.

~~29~~30. Chen J, Lan J, Liu D, Backman LJ, Zhang W, Zhou Q, et al. Ascorbic Acid Promotes the Stemness of Corneal Epithelial Stem/Progenitor Cells and Accelerates Epithelial Wound Healing in the Cornea. Stem Cells Transl Med. 2017;6(5):1356-65. Epub 2017/03/10. doi: 10.1002/sctm.16-0441. PubMed PMID: 28276172; PubMed Central PMCID: PMCPMC5442716.

~~30~~31. Peyman GA, Kivilcim M, Morales AM, DellaCroce JT, Conway MD. Inhibition of corneal angiogenesis by ascorbic acid in the rat model. Graefes Arch Clin Exp Ophthalmol. 2007;245(10):1461-7. Epub 2007/02/24. doi: 10.1007/s00417-007-0542-4. PubMed PMID: 17318569.

~~31~~32. Lee MY, Chung SK. Treatment of corneal neovascularization by topical application of ascorbic acid in the rabbit model. Cornea. 2012;31(10):1165-9. Epub 2012/07/27. doi: 10.1097/ICO.0b013e318241433b. PubMed PMID: 22832865.

Supplementary Figure Captions

Supplementary Figure S1. Phase-contrast images and ICC-Staining for ACTA2, VIM, FN1, and F-Actin on human TFs and SFs.

TFs (upper row) and SFs (lower row) at passage # 3 were cultured for 1 week and ICC-stained for ACTA2, VIM, or Phalloidin-Red to visualize F-Actin. Nuclei were stained with DAPI, and the scale bar represents 50 µm.

Supplementary Figure S2. ICC-staining of human TFs and their ability to induce TGFB2-mediated endothelial-to-mesenchymal transition (EndoTM).

Representative ICC results from human TFs treated without (upper row) or with recombinant TGFB2 (lower row) for one week. Cells were immunostained for ACTA2, FN1, VIM, or Phalloidin-Red to visualize F-Actin. The scale bar represents 50 µm.

Supplementary Figure S3. Hoechst 33342 does not impact the AA-induced cell death.

Cytotoxicity analysis of TFs (left) and SFs (right) ~~that were either not or~~ exposed to 6 mM AA in combination with or without 28 nM Hoechst 33342. The y-axis shows the total number of PI-positive nuclei.

Supplementary Figure S4. Catalase prevents hydrogen peroxide-induced cell death in both SFs and TFs.

The relative numbers of dead TFs and SFs were calculated in two-hour intervals by dividing the number of PI-positive cells by the number of Hoechst-positive cells treated without or with 400 µM (left) or 600 µM (right) hydrogen peroxide without or with 500 Units Catalase for 24 h.

Supplementary Figure S5

A-C) Raw WB images used for Fig. 3 b and WB quantification

Supplementary Video Captions

Supplementary Video S1. Phase contrast time-lapse image videos of TFs between days 6 and 7 after initial culture were treated intraoperatively without or with 0.02 % MMC for 3 minutes. The scale bar presents 1000 µm.

Supplementary Video S2. Time-lapse image videos showing co-cultured TFs and SFs treated *in vitro* either with 0.04 % MMC for 5 minutes (left video) or permanently exposed to 0.001 % MMC for 90 hours (right video). Hoechst 33342 and PI were added to visualize the nuclei and numbers of dead cells. PI-negative nuclei are masked with yellow circles, whereas PI-positive nuclei are highlighted in red circles. The scale bar presents 1000 µm.

Supplementary Video S3.

Time-lapse video showing mono-cultured TFs and SFs treated without or with 6 mM AA for 36 h. In addition to the phase-contrast recording, the Hoechst-stained nuclei are shown. The scale bar presents 1000 µm.

Supplementary Video S4.

Time-lapse videos showing co-cultured TFs and SFs exposed to 6 mM AA for 24 h. In addition to the phase-contrast recording, fluorescence recordings of the Hoechst-stained nuclei, the PI-stained nuclei, and the merged Hoechst and PI recordings are shown. The scale bar presents 1000 µm.

Supplementary Video S5. Time-lapse videos showing co-cultured TFs and SFs exposed to 4 mM AA (left video) or combined with 500 U recombinant catalase (right video) for 24 h. Hoechst 33342 and PI were added to visualize the nuclei and numbers of dead cells. PI-negative nuclei are masked with yellow circles, whereas PI-positive nuclei are highlighted in red circles. The scale bar presents 1000 µm.

Supplementary Video S6. Time-lapse videos showing co-cultured TFs and SFs exposed to 500 µM hydrogen peroxide (left video) or combined with 500 U recombinant catalase (right video) for 24 h. Hoechst 33342 and PI were added to visualize the nuclei and numbers of dead cells. PI-negative nuclei are masked with yellow circles, whereas PI-positive nuclei are highlighted in red circles. The scale bar presents 1000 µm.

Medizinische Hochschule Hannover

Forschungslabor Experimentelle Augenheilkunde
Dr. rer. nat Heiko Fuchs, Laborleiter

OE6120
Heiko Fuchs
Telefon: 0511 532-7246
Fax: 0511 532-7276
fuchs.heiko@mh-hannover.de

Carl-Neuberg-Straße 1
30625 Hannover
Telefon: 0511 532-0
www.mh-hannover.de

PLOS ONE

–

29. Oktober 2024

Dear Editor, dear Reviewers,

We appreciate the thorough evaluation of our manuscript and the constructive feedback provided. Your comments have highlighted key areas for improvement. We have combined responses to overlapping comments to avoid redundancy and address each point comprehensively.

–

Response to both reviewers:

Reviewer #1: Methods and Results Sections: There is a significant overlap between the methods and results, particularly in the co-culture experiment section. Separating these sections will improve readability. I suggest clearly defining the methodology first and then explaining the results obtained.

Reviewer #2: Clear Separation of Methods and Results: There is a mix of methods and results, particularly in the co-culture experiment section involving Tenon fibroblasts (TFs) and scleral fibroblasts (SFs). These sections should be kept distinct to enhance the structure and flow of the manuscript.

Response:

We thank the reviewers for this comment. We acknowledge the suggestion to improve the separation of methods and results. However, in the case of the co-culture experiment, we intentionally provided detailed descriptions of the experimental setup in the results section to ensure that readers unfamiliar with IBIDI co-culture inserts can easily understand how both Tenon and scleral cells were spatially separated within one well. This co-culture system mimics the natural environment of these cells in the eye, where Tenon and scleral fibroblasts are neighboring but not mixed. Since MMC exposure occurs for both cell types simultaneously

during surgery, we felt it was crucial to present this information alongside the results to avoid confusion and unnecessary back-and-forth between the methods and results sections.

Furthermore, we included images of the IBIDI inserts and the methodology for live-cell imaging to demonstrate how both cell types were analyzed separately. This made the data more comprehensible and underscored our commitment to transparency. We believe that this approach offers a more transparent narrative for the reader, especially those less familiar with such experimental setups, and reassures them of the validity of our findings.

Reviewer #1: Rationale for Compound Selection: The choice of using hydrogen peroxide and ascorbic acid requires more explanation. Why were these compounds chosen, and what specific advantage do they offer over Mitomycin C (MMC)? The discussion should delve into the potential roles of these compounds in oxidative stress or ferroptosis.

Reviewer #2: Explanation for the Use of AA and H_2O_2: The rationale for choosing ascorbic acid and hydrogen peroxide is insufficiently explained. The authors should elaborate on why these compounds were selected and their advantages over MMC, particularly their role in inducing oxidative stress or ferroptosis.

Response:

We appreciate the opportunity to explain further the reasoning behind our selection of Ascorbic Acid (AA) and Hydrogen Peroxide (H_2O_2). During a compound screening as part of the Glaukom project, we aimed to identify substances that selectively induce cell death in Tenon fibroblasts (TFs) without affecting neighboring Scleral fibroblasts (SFs). The goal was to find an alternative to Mitomycin C (MMC), which does not selectively target TFs and can lead to postoperative complications.

During this screening, we identified inhibitors that triggered autophagy selectively in TFs without harming SFs. Autophagy, a process of controlled cell degradation, appeared to be a promising target. We conducted an in-silico analysis, which revealed that Ascorbic Acid had been reported to inhibit autophagy in specific cell types. Consequently, we hypothesized that AA could counteract the autophagic death induced by these inhibitors. Surprisingly, our experiments showed the opposite: AA exhibited a synergistic effect, accelerating the death of TFs instead of rescuing them. This unexpected outcome led us to investigate AA further as a stand-alone agent. Since the findings from the autophagy screening are still unpublished and will soon be submitted for publication, we cannot disclose the specific inhibitors identified. We kindly ask for your understanding on this point.

As for Hydrogen Peroxide's selection, it resulted from our observation that TFs express lower catalase levels than SFs, making them more vulnerable to oxidative stress. Our Western blot analysis confirmed this differential expression, prompting us to test whether H_2O_2 could induce selective cytotoxicity by exploiting the reduced catalase expression in TFs. Indeed, we found that H_2O_2 selectively damaged TFs while sparing SFs.

As a result, the reduced catalase expression in TFs represents an Achilles' heel, opening the door for novel therapeutic approaches to glaucoma surgery. By contrast, MMC lacks this selectivity and is associated with severe complications, such as hypotony, blebitis, and scleral thinning. We are confident that AA and H_2O_2 offer significant advantages in terms of safety and selectivity and could be promising alternatives for preventing intraoperative or postoperative fibrosis.

Reviewer #1: Objective of MMC Discussion: The manuscript includes MMC in the introduction, but its connection to the study is unclear. Is the goal to find an alternative to MMC, or is it a comparative analysis? The objective should be clearly defined.

Reviewer #2: MMC Discussion Clarification: It is unclear if the goal of the study is to replace MMC or simply to compare it with AA and H_2O_2. The objective must be stated clearly in the introduction to avoid confusion.

Response:

We appreciate the reviewers' comments and the need for clarification regarding the role of MMC in this study. To address the concern, we would like to clearly state that the primary objective of our study is to explore whether Ascorbic Acid (AA) and Hydrogen Peroxide (H2O2) can offer a more selective alternative to MMC in targeting Tenon fibroblasts, without causing damage to surrounding cell types such as scleral fibroblasts. While MMC is included for comparison, the focus of our investigation is on identifying safer and more targeted treatments for glaucoma surgery.

In Germany, MMC is widely regarded as the gold standard for preventing fibrosis after glaucoma filtration surgery, despite its well-documented side effects, such as hypotony, blebitis, and endophthalmitis. MMC's non-selective mechanism damages Tenon fibroblasts and surrounding cells, including scleral fibroblasts. This lack of specificity contributes to complications that can significantly impact patient outcomes. Given these risks, there is an urgent need for safer alternatives.

One notable practical advantage of AA is its affordability, but this could also present a challenge in securing funding for clinical off-label studies. Due to the low financial return on investment, sponsors may have little incentive to fund trials, which could delay AA's adoption in clinical practice. Despite these economic challenges, we believe AA's potential benefits, including its safety profile and effectiveness, warrant further investigation.

To simulate the intraoperative application of MMC as commonly used in glaucoma surgeries, we employed a 5-minute exposure in our experiments. This exposure duration aligns with clinical practice, where MMC is typically applied at concentrations of 0.02%-0.04% for 2-5 minutes. By using this approach in our co-culture experiments with both Tenon and Scleral fibroblasts, we closely mimicked surgical conditions to examine the differential effects of MMC on these two cell types.

Our findings, including the Lethal Time 50 (LT_{50}) analysis, demonstrated a significant difference in cell death kinetics between scleral fibroblasts (SFs) and Tenon fibroblasts (TFs). Specifically, scleral cells died faster than Tenon cells when exposed to MMC, reinforcing that MMC, while effective, lacks the cell selectivity required for targeted therapy in glaucoma surgery.

By employing a co-culture setup, we were able to precisely monitor the effects of MMC on both cell types under identical conditions. This experiment provided valuable insights into MMC's non-selective cytotoxicity and underscored the need to explore alternative agents, such as AA and H_2O_2, that could selectively target Tenon fibroblasts without harming surrounding tissues. These findings will be clearly articulated in the revised manuscript.

To avoid any misunderstanding and to clarify the study's objective, we have changed the title of our manuscript from "Selective Cytotoxicity of Ascorbic Acid and Hydrogen Peroxide on Primary Human Tenon Fibroblasts without Harming Scleral Fibroblasts In Vitro" to **"Selective Toxicity of Ascorbic Acid and Hydrogen Peroxide on Human Tenon Cells without Harming Scleral Cells In Vitro: A**

Possible Alternative to Non-Selective Mitomycin C?" This title better reflects our aim to identify safer alternatives to MMC for potential clinical use.

Unfortunately, due to ethical considerations, we have not been able to conduct *in vivo* studies to date. However, we hope that after the publication of this study, we will have provided sufficient evidence to justify future animal studies or off-label clinical trials.

Reviewer #1: High Passage Number for TFs: Using passage 10 Tenon fibroblasts raises concerns since higher passages might behave differently than early passage cells. A justification for choosing this passage number is required to address potential concerns about altered cell behavior.

Reviewer #2: Passage Number for TFs and SFs: The use of passage 10 TFs is concerning, as high-passage fibroblasts may differ from early-passage cells in terms of behavior. Additionally, the passage number for scleral fibroblasts (SFs) is missing and should be included in the methods for consistency and reproducibility.

Response:

The mention of passage 10 in the results section refers **only** to the fact that Tenon cells could be successfully expanded until passage 10 and beyond, regardless of prior intraoperative MMC treatment. This finding was included to highlight that MMC did not have a lasting inhibitory effect on cell proliferation ex vivo, which raises valid concerns about its efficacy in clinical use. However, the experiments presented in the manuscript were **not** conducted with passage 10 cells.

Both reviewers may have overlooked the details in the **Methods** section regarding the passage numbers of the Tenon fibroblasts (TFs) and Scleral fibroblasts (SFs). We want to clarify that the cells used in our experiments were **between passages 2 and 8**, as clearly stated in the manuscript. This passage range was chosen to ensure optimal cell behavior and reproducibility.

Moreover, we used patient-matched Tenon and Scleral fibroblasts (SFs) within similar passage ranges to ensure consistency and reproducibility. We maintained a strict protocol to use only passage-matched cells with a variation of ±1 passage number. The results presented in the manuscript are based on three technical replicates for each concentration, and the experiments were repeated three 2 to 6 times, necessitating higher passage cells due to the large number of cells required for these experiments. Only early passage cells (e.g., passage 3 or 4) would not have provided sufficient material for replications and statistical analysis.

In addition, it is difficult to define a universal "limit" for the passage number beyond which primary cells become unsuitable for *in vitro* studies, as this can vary greatly depending on the cell type and cell culture conditions, such as seeding density. For example, fibroblasts are more robust and can be used at higher passages than more delicate cells, such as neurons. For consistency, the passage number for SFs will also be included in the revised manuscript.

Finally, the fact that we observed consistent results across multiple independent experiments, despite using higher passage numbers, strongly suggests that the effects we report, whether with MMC, AA, or H_2O_2, are not passage-number dependent.

However, to avoid confusion, we revised that section stating now:" ***Remarkably, two out of six tenon tissue samples were treated intraoperatively with 0.04 % MMC for 3 to 5 minutes before***

the tissue was removed, whereas the other four tenon tissues were removed before MMC was applied. However, an outgrowth of proliferating TFs was observed in all tenon tissue samples after 5-7 days, regardless of whether they were treated intraoperatively with MMC (Fig. 1a+b, Supplementary Video S1). We could expand successfully and culture TFs up to and beyond passage 10, regardless of intraoperative MMC treatment. This observation suggests that MMC did not exert a lasting antiproliferative effect in ex vivo conditions."

Reviewer #1: Statistical Methods: The use of a t-test in comparing more than two groups is not appropriate. A better statistical approach, such as ANOVA, should be applied when comparing three groups.

Reviewer #2: Statistical Analysis Issues: In some comparisons where more than two groups are analyzed, a t-test is used, which is inappropriate. A more suitable statistical method like ANOVA should be used.

Response:

We thank the reviewer for pointing out the issue regarding the statistical methods. We acknowledge that a t-test is not the optimal choice when comparing more than two groups. For comparisons involving multiple groups, we should indeed have applied a one-way ANOVA to ensure the correct statistical treatment of the data.

In the revised manuscript, we have replaced the t-tests with ANOVA where applicable and report the results accordingly. Additionally, for post hoc analysis, we will include appropriate tests (e.g., Tukey's HSD) to identify significant differences between specific groups, if needed.

During our re-evaluation of the statistical analysis, we noted an error in the calculation of significances for the Western blot quantification in Figure 4. Upon applying the appropriate ANOVA approach, we found discrepancies in the initial significance results. We have corrected these calculations and revised Figure 4 accordingly to accurately represent the statistical outcomes.

Response to Reviewer #1

Reviewer #1: TGF-β Inclusion: The rationale for using Transforming Growth Factor-beta (TGF-β) is unclear. The authors should explain the reason for incorporating TGF-β, especially given its role in fibrosis.

Response:

We appreciate the reviewer's request for clarification. As described in the literature, TGF-β was incorporated into our experiments to demonstrate that the Tenon fibroblasts (TFs) used in the study respond to TGF-β by undergoing epithelial-mesenchymal transition (EMT). The response to TGF-β is a crucial marker of the ability of TFs to react to fibrotic stimuli. To confirm this, we conducted immunocytochemical (ICC) staining, revealing that after one week of exposure to TGF-β, the TFs show the typical expression and distribution of EMT markers such as ACTA2, fibronectin, and vimentin. These markers clearly showed that the TFs responded as expected under pro-fibrotic conditions.

We will clarify this rationale in the revised manuscript and provide more detail.

Reviewer #1: Omission in Methods: Line 241 references treatments with MMC, but this is missing from the methods section. All treatments and procedures should be clearly outlined for better transparency.

Response:

We appreciate the reviewer pointing out this omission in the methods section.

We apologize for this oversight and correct it in the revision, Stating:

“Mitomycin C (MMC) Treatment

For the intraoperative application of Mitomycin C (MMC), a concentration of 0.04% MMC was prepared and applied using a sponge. The MMC was left in contact with the tissue for three minutes to allow for adequate absorption. Following the application, the area was washed twice with 10 ml of Hanks' Balanced Salt Solution (HBSS) to remove any residual MMC.

For the co-culture experiments,…”

Reviewer #1: pH Measurement: Only the pH of the 8 mM AA solution is reported. It is important to confirm whether other concentrations maintained a stable pH, as this could affect the results.

Response:

We appreciate the reviewer's request for clarification regarding the pH of the AA solutions. The pH of the highest concentration of AA (8 mM) was measured to be 6.8, which prompted us to include an HCl control to verify that the reduction in pH did not cause cytotoxicity. This control experiment demonstrated that the observed cell death was not due to the pH drop but to the effects of AA.

Given that the pH of the 8 mM AA solution had no cytotoxic impact when mimicked by the HCl control, it stands to reason that AA solutions with pH values between 6.8 and 7.4 (which would correspond to the lower AA concentrations) would also not cause significant cytotoxicity based solely on pH changes.

We will update the manuscript to include this clarification and ensure the reasoning for focusing on the 8 mM concentration is clear.

Reviewer #1: Summary Revision: The summary contains information that does not directly reflect the study's results. The authors should revise the summary to focus only on their experimental findings.

We have revised the summary to ensure it directly reflects the experimental findings of the study, stating:

"**Background**: Glaucoma, a leading cause of blindness, is often driven by elevated intraocular pressure (IOP), which damages the optic nerve. Transscleral filtration surgery reduces IOP but is frequently complicated by excessive wound healing from Tenon fibroblasts (TFs), impeding aqueous humor absorption. Mitomycin C (MMC), used for over 30 years in ophthalmic surgeries, inhibits TF

proliferation but carries significant side effects, including hypotony, blebitis, and endophthalmitis, due to its non-selective cytotoxicity. MMC's inability to entirely prevent fibrosis increases surgical failure risk, often necessitating further interventions like bleb needling. This study investigates whether ascorbic acid (AA) and hydrogen peroxide (H_2O_2) can selectively target TFs without damaging scleral fibroblasts (SFs) *in vitro*, using MMC as a benchmark.

Methods: Primary human TFs and SFs were cultured from patient trabeculectomy tissues. Cells were treated with various concentrations of MMC, AA, or H_2O_2. Cytotoxic effects were analyzed via live-cell imaging. Catalase expression in both cell types was assessed by immunocytochemistry and Western blot, and recombinant catalase was used to validate its protective effect against AA- and H_2O_2-induced cell death.

Results: Short-term exposure (5 min) to 0.02%-0.04% MMC or long-term exposure to 0.00025%-0.001% MMC caused cytotoxicity in TFs and SFs, with SFs dying significantly earlier. In contrast, AA (6–8 mM) selectively induced cell death in TFs without harming SFs. H_2O_2 also showed selective cytotoxicity towards TFs. Lower catalase expression in TFs compared to SFs was **determined via Western blot and immunocytochemistry**, highlighting a mechanism for this selective effect. Recombinant catalase neutralized the cytotoxic effects of AA and H_2O_2 on TFs.

Conclusions: Unlike MMC, Ascorbic acid and hydrogen peroxide exhibit selective cytotoxicity towards Tenon fibroblasts, which may provide a safer, more targeted approach for preventing fibrosis in glaucoma surgery. Further *in vivo* studies are needed to explore the clinical applicability of these findings."

<u>Response to Reviewer #2</u>

Reviewer # 2: Discrepancy in AA Results: The authors should address the discrepancy between their results and Jampel et al.'s study regarding the cytotoxicity of AA. A discussion on why the current study required higher concentrations of AA would help clarify the differences between findings.
Response:

We want to address the discrepancy between our study and the findings by Jampel et al. from 1990. Jampel's study tested ascorbic acid (AA) at concentrations approximating those found in the aqueous humor of patients, around 1.1 mM. His approach was based on the assumption that the physiological concentration of AA within the eye might inhibit Tenon fibroblast proliferation. However, the *in vitro* environment used in Jampel's study, focusing on low concentrations of AA, may not fully represent AA's potential for selective cytotoxicity on TFs.

In addition to this concentration difference, it is crucial to highlight that while Jampel observed impaired viability of Tenon fibroblasts, his study did not explicitly investigate the concentration thresholds at which AA becomes cytotoxic. His research primarily centered on the inhibitory effect of AA on cell proliferation rather than pinpointing specific cytotoxic concentrations that selectively target Tenon fibroblasts.

In contrast, our study explored a broader range of AA concentrations, identifying 6-8 mM as the most effective in achieving selective cytotoxicity of Tenon fibroblasts while sparing neighboring scleral fibroblasts. This concentration range offers a more targeted strategy for reducing fibrosis. Additionally, our use of live-cell imaging and co-culture experiments, alongside extended observation periods, allowed for a more comprehensive understanding of AA's selective impact.

By expanding beyond the concentrations tested by Jampel, we observed more pronounced selective cytotoxicity, which may not have been apparent at lower concentrations. This broader range of AA concentrations highlights its potential utility in glaucoma filtration surgeries, where higher doses could more effectively prevent excessive wound healing without harming adjacent tissues.

To address these points, we have revised the discussion section to clarify the differences between our findings and those of Jampel et al., specifically regarding the concentration-dependent cytotoxic effects of AA.

Lastly, we sincerely appreciate the reviewers' valuable insights and the opportunity to improve our manuscript. We believe that these revisions have strengthened our work, and we are hopeful that it meets the standards for publication in PLOS ONE and remain with kind regards.

Heiko Fuchs

Corresponding Author

Contact:
Dr. rer. nat Heiko Fuchs, Laboratory Manager
Research Laboratory Experimental Ophthalmology
Hannover Medical School
Feodor-Lynen Strasse 35
30625 Hannover
Tel.: + 49511 532-7246 Fax: + 49511 532-7276
Email: fuchs.heiko@mh-hannover.de

Additional Contact:
Prof. Dr. med. Carsten Framme, Director of the eye clinic
University Hospital for Ophthalmology
Hannover Medical School
Carl-Neuberg Strasse 1
30625 Hannover
Tel.: + 49511 532-3060 Fax: + 49511 532-3050
Email: framme.carsten@mh-hannover.de
