# Supplementary material for: Selective toxicity of ascorbic acid and hydrogen peroxide on human tenon cells without harming scleral cells in vitro: A possible alternative to non-selective mitomycin C?
Source: PLoS One. 2025 Apr 2;20(4):e0320558. doi: 10.1371/journal.pone.0320558 (PMC11964264; doi:10.1371/journal.pone.0320558)
Supplement: Attachment — Submitted filename: Rebuttalletterdoc.doc [file pone.0320558.s011.doc]

**Forschungslabor Experimentelle Augenheilkunde**

**Dr. rer. nat Heiko Fuchs, Laborleiter**

OE6120

Heiko Fuchs

Telefon:	0511 532-7246

Fax:	0511 532-7276

fuchs.heiko@mh-hannover.de

Carl-Neuberg-Straße 1

30625 Hannover

Telefon: 0511 532-0

www.mh-hannover.de

*PLOS ONE*

30. November 2026

Dear Editor, dear Reviewers,

We appreciate the thorough evaluation of our manuscript and the constructive feedback provided. Your comments have highlighted key areas for improvement. We have combined responses to overlapping comments to avoid redundancy and address each point comprehensively.

**Response to both reviewers:**

**Reviewer #1: Methods and Results Sections: There is a significant overlap between the methods and results, particularly in the co-culture experiment section. Separating these sections will improve readability. I suggest clearly defining the methodology first and then explaining the results obtained.**

**Reviewer #2: Clear Separation of Methods and Results: There is a mix of methods and results, particularly in the co-culture experiment section involving Tenon fibroblasts (TFs) and scleral fibroblasts (SFs). These sections should be kept distinct to enhance the structure and flow of the manuscript.**

**Response:**

**We thank the reviewers for this comment. We acknowledge the suggestion to improve the separation of methods and results. However, in the case of the co-culture experiment, we intentionally provided detailed descriptions of the experimental setup in the results section to ensure that readers unfamiliar with IBIDI co-culture inserts can easily understand how both Tenon and scleral cells were spatially separated within one well. This co-culture system mimics the natural environment of these cells in the eye, where Tenon and scleral fibroblasts are neighboring but not mixed. Since MMC exposure occurs for both cell types simultaneously during surgery, we felt it was crucial to present this information alongside the results to avoid confusion and unnecessary back-and-forth between the methods and results sections.**

**Furthermore, we included images of the IBIDI inserts and the methodology for live-cell imaging to demonstrate how both cell types were analyzed separately. This made the data more comprehensible and underscored our commitment to transparency. We believe that this approach offers a more transparent narrative for the reader, especially those less familiar with such experimental setups, and reassures them of the validity of our findings.**

Reviewer #1: Rationale for Compound Selection: The choice of using hydrogen peroxide and ascorbic acid requires more explanation. Why were these compounds chosen, and what specific advantage do they offer over Mitomycin C (MMC)? The discussion should delve into the potential roles of these compounds in oxidative stress or ferroptosis.

Reviewer #2: Explanation for the Use of AA and H_2_O_2_: The rationale for choosing ascorbic acid and hydrogen peroxide is insufficiently explained. The authors should elaborate on why these compounds were selected and their advantages over MMC, particularly their role in inducing oxidative stress or ferroptosis.

Response:

We appreciate the opportunity to explain further the reasoning behind our selection of Ascorbic Acid (AA) and Hydrogen Peroxide (H_2_O_2_). During a compound screening as part of the Glaukom project, we aimed to identify substances that selectively induce cell death in Tenon fibroblasts (TFs) without affecting neighboring Scleral fibroblasts (SFs). The goal was to find an alternative to Mitomycin C (MMC), which does not selectively target TFs and can lead to postoperative complications.

During this screening, we identified inhibitors that triggered autophagy selectively in TFs without harming SFs. Autophagy, a process of controlled cell degradation, appeared to be a promising target. We conducted an in-silico analysis, which revealed that Ascorbic Acid had been reported to inhibit autophagy in specific cell types. Consequently, we hypothesized that AA could counteract the autophagic death induced by these inhibitors. Surprisingly, our experiments showed the opposite: AA exhibited a synergistic effect, accelerating the death of TFs instead of rescuing them. This unexpected outcome led us to investigate AA further as a stand-alone agent. Since the findings from the autophagy screening are still unpublished and will soon be submitted for publication, we cannot disclose the specific inhibitors identified. We kindly ask for your understanding on this point.

As for Hydrogen Peroxide's selection, it resulted from our observation that TFs express lower catalase levels than SFs, making them more vulnerable to oxidative stress. Our Western blot analysis confirmed this differential expression, prompting us to test whether H_2_O_2_ could induce selective cytotoxicity by exploiting the reduced catalase expression in TFs. Indeed, we found that H_2_O_2_ selectively damaged TFs while sparing SFs.

As a result, the reduced catalase expression in TFs represents an Achilles' heel, opening the door for novel therapeutic approaches to glaucoma surgery. By contrast, MMC lacks this selectivity and is associated with severe complications, such as hypotony, blebitis, and scleral thinning. We are confident that AA and H_2_O_2_ offer significant advantages in terms of safety and selectivity and could be promising alternatives for preventing intraoperative or postoperative fibrosis.

Reviewer #1: Objective of MMC Discussion: The manuscript includes MMC in the introduction, but its connection to the study is unclear. Is the goal to find an alternative to MMC, or is it a comparative analysis? The objective should be clearly defined.

Reviewer #2: MMC Discussion Clarification: It is unclear if the goal of the study is to replace MMC or simply to compare it with AA and H_2_O_2_. The objective must be stated clearly in the introduction to avoid confusion.

**Response:**

We appreciate the reviewers' comments and the need for clarification regarding the role of MMC in this study. To address the concern, we would like to clearly state that the primary objective of our study is to explore whether Ascorbic Acid (AA) and Hydrogen Peroxide (H2O2) can offer a more selective alternative to MMC in targeting Tenon fibroblasts, without causing damage to surrounding cell types such as scleral fibroblasts. While MMC is included for comparison, the focus of our investigation is on identifying safer and more targeted treatments for glaucoma surgery.

In Germany, MMC is widely regarded as the gold standard for preventing fibrosis after glaucoma filtration surgery, despite its well-documented side effects, such as hypotony, blebitis, and endophthalmitis. MMC's non-selective mechanism damages Tenon fibroblasts and surrounding cells, including scleral fibroblasts. This lack of specificity contributes to complications that can significantly impact patient outcomes. Given these risks, there is an urgent need for safer alternatives.

One notable practical advantage of AA is its affordability, but this could also present a challenge in securing funding for clinical off-label studies. Due to the low financial return on investment, sponsors may have little incentive to fund trials, which could delay AA’s adoption in clinical practice. Despite these economic challenges, we believe AA’s potential benefits, including its safety profile and effectiveness, warrant further investigation.

To simulate the intraoperative application of MMC as commonly used in glaucoma surgeries, we employed a 5-minute exposure in our experiments. This exposure duration aligns with clinical practice, where MMC is typically applied at concentrations of 0.02%-0.04% for 2-5 minutes. By using this approach in our co-culture experiments with both Tenon and Scleral fibroblasts, we closely mimicked surgical conditions to examine the differential effects of MMC on these two cell types.

Our findings, including the Lethal Time 50 (LT_50_) analysis, demonstrated a significant difference in cell death kinetics between scleral fibroblasts (SFs) and Tenon fibroblasts (TFs). Specifically, scleral cells died faster than Tenon cells when exposed to MMC, reinforcing that MMC, while effective, lacks the cell selectivity required for targeted therapy in glaucoma surgery.

By employing a co-culture setup, we were able to precisely monitor the effects of MMC on both cell types under identical conditions. This experiment provided valuable insights into MMC's non-selective cytotoxicity and underscored the need to explore alternative agents, such as AA and H_2_O_2_, that could selectively target Tenon fibroblasts without harming surrounding tissues. These findings will be clearly articulated in the revised manuscript.

To avoid any misunderstanding and to clarify the study's objective, we have changed the title of our manuscript from "Selective Cytotoxicity of Ascorbic Acid and Hydrogen Peroxide on Primary Human Tenon Fibroblasts without Harming Scleral Fibroblasts In Vitro" to **"Selective Toxicity of Ascorbic Acid and Hydrogen Peroxide on Human Tenon Cells without Harming Scleral Cells In Vitro: A Possible Alternative to Non-Selective Mitomycin C?"** This title better reflects our aim to identify safer alternatives to MMC for potential clinical use.

Unfortunately, due to ethical considerations, we have not been able to conduct *in vivo* studies to date. However, we hope that after the publication of this study, we will have provided sufficient evidence to justify future animal studies or off-label clinical trials.

*Reviewer #1: High Passage Number for TFs: Using passage 10 Tenon fibroblasts raises concerns since higher passages might behave differently than early passage cells. A justification for choosing this passage number is required to address potential concerns about altered cell behavior.*

*Reviewer #2: Passage Number for TFs and SFs: The use of passage 10 TFs is concerning, as high-passage fibroblasts may differ from early-passage cells in terms of behavior. Additionally, the passage number for scleral fibroblasts (SFs) is missing and should be included in the methods for consistency and reproducibility.*

**Response:**

The mention of passage 10 in the results section refers **only** to the fact that Tenon cells could be successfully expanded until passage 10 and beyond, regardless of prior intraoperative MMC treatment. This finding was included to highlight that MMC did not have a lasting inhibitory effect on cell proliferation ex vivo, which raises valid concerns about its efficacy in clinical use. However, the experiments presented in the manuscript were **not** conducted with passage 10 cells.

Both reviewers may have overlooked the details in the **Methods** section regarding the passage numbers of the Tenon fibroblasts (TFs) and Scleral fibroblasts (SFs). We want to clarify that the cells used in our experiments were **between passages 2 and 8**, as clearly stated in the manuscript. This passage range was chosen to ensure optimal cell behavior and reproducibility.

Moreover, we used patient-matched Tenon and Scleral fibroblasts (SFs) within similar passage ranges to ensure consistency and reproducibility. We maintained a strict protocol to use only passage-matched cells with a variation of ±1 passage number. The results presented in the manuscript are based on three technical replicates for each concentration, and the experiments were repeated three 2 to 6 times, necessitating higher passage cells due to the large number of cells required for these experiments. Only early passage cells (e.g., passage 3 or 4) would not have provided sufficient material for replications and statistical analysis.

In addition, it is difficult to define a universal "limit" for the passage number beyond which primary cells become unsuitable for *in vitro* studies, as this can vary greatly depending on the cell type and cell culture conditions, such as seeding density. For example, fibroblasts are more robust and can be used at higher passages than more delicate cells, such as neurons. For consistency, the passage number for SFs will also be included in the revised manuscript.

Finally, the fact that we observed consistent results across multiple independent experiments, despite using higher passage numbers, strongly suggests that the effects we report, whether with MMC, AA, or H_2_O_2,_ are not passage-number dependent.

However, to avoid confusion, we revised that section stating now:" ***Remarkably, two out of six tenon tissue samples were treated intraoperatively with 0.04 % MMC for 3 to 5 minutes before the tissue was removed, whereas the other four tenon tissues were removed before MMC was applied. However, an outgrowth of proliferating TFs was observed in all tenon tissue samples after 5-7 days, regardless of whether they were treated intraoperatively with MMC (Fig. 1a+b, Supplementary Video S1). We could expand successfully and culture TFs up to and beyond passage 10, regardless of intraoperative MMC treatment. This observation suggests that MMC did not exert a lasting antiproliferative effect in ex vivo conditions."***

Reviewer #1: Statistical Methods: The use of a t-test in comparing more than two groups is not appropriate. A better statistical approach, such as ANOVA, should be applied when comparing three groups.

Reviewer #2: Statistical Analysis Issues: In some comparisons where more than two groups are analyzed, a t-test is used, which is inappropriate. A more suitable statistical method like ANOVA should be used.

**Response:**

We thank the reviewer for pointing out the issue regarding the statistical methods. We acknowledge that a t-test is not the optimal choice when comparing more than two groups. For comparisons involving multiple groups, we should indeed have applied a one-way ANOVA to ensure the correct statistical treatment of the data.

In the revised manuscript, we have replaced the t-tests with ANOVA where applicable and report the results accordingly. Additionally, for post hoc analysis, we will include appropriate tests (e.g., Tukey's HSD) to identify significant differences between specific groups, if needed.

During our re-evaluation of the statistical analysis, we noted an error in the calculation of significances for the Western blot quantification in Figure 4. Upon applying the appropriate ANOVA approach, we found discrepancies in the initial significance results. We have corrected these calculations and revised Figure 4 accordingly to accurately represent the statistical outcomes.

**Response to Reviewer #1**

Reviewer #1: TGF-β Inclusion: The rationale for using Transforming Growth Factor-beta (TGF-β) is unclear. The authors should explain the reason for incorporating TGF-β, especially given its role in fibrosis.

**Response:**

We appreciate the reviewer's request for clarification. As described in the literature, TGF-β was incorporated into our experiments to demonstrate that the Tenon fibroblasts (TFs) used in the study respond to TGF-β by undergoing epithelial-mesenchymal transition (EMT). The response to TGF-β is a crucial marker of the ability of TFs to react to fibrotic stimuli. To confirm this, we conducted immunocytochemical (ICC) staining, revealing that after one week of exposure to TGF-β, the TFs show the typical expression and distribution of EMT markers such as ACTA2, fibronectin, and vimentin. These markers clearly showed that the TFs responded as expected under pro-fibrotic conditions.

We will clarify this rationale in the revised manuscript and provide more detail.

Reviewer #1: Omission in Methods: Line 241 references treatments with MMC, but this is missing from the methods section. All treatments and procedures should be clearly outlined for better transparency.

**Response:**

We appreciate the reviewer pointing out this omission in the methods section.

We apologize for this oversight and correct it in the revision, Stating:

**“Mitomycin C (MMC) Treatment**

For the intraoperative application of Mitomycin C (MMC), a concentration of 0.04% MMC was prepared and applied using a sponge. The MMC was left in contact with the tissue for three minutes to allow for adequate absorption. Following the application, the area was washed twice with 10 ml of Hanks' Balanced Salt Solution (HBSS) to remove any residual MMC.

For the co-culture experiments,…”

Reviewer #1: pH Measurement: Only the pH of the 8 mM AA solution is reported. It is important to confirm whether other concentrations maintained a stable pH, as this could affect the results.

**Response:**

We appreciate the reviewer's request for clarification regarding the pH of the AA solutions. The pH of the highest concentration of AA (8 mM) was measured to be 6.8, which prompted us to include an HCl control to verify that the reduction in pH did not cause cytotoxicity. This control experiment demonstrated that the observed cell death was not due to the pH drop but to the effects of AA.

Given that the pH of the 8 mM AA solution had no cytotoxic impact when mimicked by the HCl control, it stands to reason that AA solutions with pH values between 6.8 and 7.4 (which would correspond to the lower AA concentrations) would also not cause significant cytotoxicity based solely on pH changes.

We will update the manuscript to include this clarification and ensure the reasoning for focusing on the 8 mM concentration is clear.

Reviewer #1: Summary Revision: The summary contains information that does not directly reflect the study's results. The authors should revise the summary to focus only on their experimental findings.

We have revised the summary to ensure it directly reflects the experimental findings of the study, stating:

**"Background**: Glaucoma, a leading cause of blindness, is often driven by elevated intraocular pressure (IOP), which damages the optic nerve. Transscleral filtration surgery reduces IOP but is frequently complicated by excessive wound healing from Tenon fibroblasts (TFs), impeding aqueous humor absorption. Mitomycin C (MMC), used for over 30 years in ophthalmic surgeries, inhibits TF proliferation but carries significant side effects, including hypotony, blebitis, and endophthalmitis, due to its non-selective cytotoxicity. MMC's inability to entirely prevent fibrosis increases surgical failure risk, often necessitating further interventions like bleb needling. This study investigates whether ascorbic acid (AA) and hydrogen peroxide (H_2_O_2_) can selectively target TFs without damaging scleral fibroblasts (SFs) *in vitro*, using MMC as a benchmark.

**Methods**: Primary human TFs and SFs were cultured from patient trabeculectomy tissues. Cells were treated with various concentrations of MMC, AA, or H_2_O_2_. Cytotoxic effects were analyzed via live-cell imaging. Catalase expression in both cell types was assessed by immunocytochemistry and Western blot, and recombinant catalase was used to validate its protective effect against AA- and H_2_O_2_-induced cell death.

**Results**: Short-term exposure (5 min) to 0.02%-0.04% MMC or long-term exposure to 0.00025%-0.001% MMC caused cytotoxicity in TFs and SFs, with SFs dying significantly earlier. In contrast, AA (6–8 mM) selectively induced cell death in TFs without harming SFs. H_2_O_2_ also showed selective cytotoxicity towards TFs. Lower catalase expression in TFs compared to SFs was **determined via Western blot and immunocytochemistry**, highlighting a mechanism for this selective effect. Recombinant catalase neutralized the cytotoxic effects of AA and H_2_O_2_ on TFs.

**Conclusions**: Unlike MMC, Ascorbic acid and hydrogen peroxide exhibit selective cytotoxicity towards Tenon fibroblasts, which may provide a safer, more targeted approach for preventing fibrosis in glaucoma surgery. Further *in vivo* studies are needed to explore the clinical applicability of these findings."

**Response to Reviewer #2**

Reviewer # 2: Discrepancy in AA Results: The authors should address the discrepancy between their results and Jampel et al.'s study regarding the cytotoxicity of AA. A discussion on why the current study required higher concentrations of AA would help clarify the differences between findings.

**Response:**

We want to address the discrepancy between our study and the findings by Jampel et al. from 1990. Jampel's study tested ascorbic acid (AA) at concentrations approximating those found in the aqueous humor of patients, around 1.1 mM. His approach was based on the assumption that the physiological concentration of AA within the eye might inhibit Tenon fibroblast proliferation. However, the *in vitro* environment used in Jampel's study, focusing on low concentrations of AA, may not fully represent AA's potential for selective cytotoxicity on TFs.

In addition to this concentration difference, it is crucial to highlight that while Jampel observed impaired viability of Tenon fibroblasts, his study did not explicitly investigate the concentration thresholds at which AA becomes cytotoxic. His research primarily centered on the inhibitory effect of AA on cell proliferation rather than pinpointing specific cytotoxic concentrations that selectively target Tenon fibroblasts.

In contrast, our study explored a broader range of AA concentrations, identifying 6-8 mM as the most effective in achieving selective cytotoxicity of Tenon fibroblasts while sparing neighboring scleral fibroblasts. This concentration range offers a more targeted strategy for reducing fibrosis. Additionally, our use of live-cell imaging and co-culture experiments, alongside extended observation periods, allowed for a more comprehensive understanding of AA's selective impact.

By expanding beyond the concentrations tested by Jampel, we observed more pronounced selective cytotoxicity, which may not have been apparent at lower concentrations. This broader range of AA concentrations highlights its potential utility in glaucoma filtration surgeries, where higher doses could more effectively prevent excessive wound healing without harming adjacent tissues.

To address these points, we have revised the discussion section to clarify the differences between our findings and those of Jampel et al., specifically regarding the concentration-dependent cytotoxic effects of AA.

Lastly, we sincerely appreciate the reviewers' valuable insights and the opportunity to improve our manuscript. We believe that these revisions have strengthened our work, and we are hopeful that it meets the standards for publication in PLOS ONE and remain with kind regards.

Heiko Fuchs

Corresponding Author

Contact:

Dr. rer. nat Heiko Fuchs, Laboratory Manager

Research Laboratory Experimental Ophthalmology

Hannover Medical School

Feodor-Lynen Strasse 35

30625 Hannover

Tel.: + 49511 532-7246	Fax: + 49511 532-7276

Email: fuchs.heiko@mh-hannover.de

Additional Contact:

Prof. Dr. med. Carsten Framme, Director of the eye clinic

University Hospital for Ophthalmology

Hannover Medical School

Carl-Neuberg Strasse 1

30625 Hannover

Tel.: + 49511 532-3060	Fax: + 49511 532-3050

Email: [framme.carsten@mh-hannover.de](mailto:framme.carsten@mh-hannover.de)
