# Supplementary material for: Selective toxicity of ascorbic acid and hydrogen peroxide on human tenon cells without harming scleral cells in vitro: A possible alternative to non-selective mitomycin C?
Source: PLoS One. 2025 Apr 2;20(4):e0320558. doi: 10.1371/journal.pone.0320558 (PMC11964264; doi:10.1371/journal.pone.0320558)
Supplement: Attachment — Submitted filename: Rebuttalletterdoc_auresp_2.doc [file pone.0320558.s012.doc]

**Forschungslabor Experimentelle Augenheilkunde**

**Dr. rer. nat Heiko Fuchs, Laborleiter**

OE6120

Heiko Fuchs

Telefon:	0511 532-7246

Fax:	0511 532-7276

fuchs.heiko@mh-hannover.de

Carl-Neuberg-Straße 1

30625 Hannover

Telefon: 0511 532-0

www.mh-hannover.de

*PLOS ONE*

18th February 2025

Dear Reviewers,

We sincerely appreciate the opportunity to submit a revised version of our manuscript titled "Selective Toxicity of Ascorbic Acid and Hydrogen Peroxide on Human Tenon Cells without Harming Scleral Cells In Vitro: A Possible Alternative to Non-Selective Mitomycin C?" (Manuscript ID: PONE-D-24-26851R1). We thank you and the reviewers for your constructive feedback, which has helped us improve the quality of our work.

We are pleased to note that Reviewer #3 has confirmed that all comments raised in the previous round of review have been adequately addressed. Moreover, the reviewer has affirmed that the manuscript is technically sound, the statistical analyses have been conducted appropriately, and the data fully support our conclusions. Additionally, the reviewer confirmed that all data are fully available and that the manuscript is well-written and intelligible.

We also sincerely appreciate the reviewer’s detailed corrections and suggestions within the manuscript. We have carefully reviewed and incorporated these proposed changes to further enhance clarity and accuracy.

As no further specific concerns or revision requests have been raised by the reviewers, we have carefully reviewed the manuscript to ensure clarity, correctness, and compliance with the journal’s formatting and reference requirements. We have also verified that all cited references are accurate and up to date, in line with the editorial guidelines.

Accordingly, we are resubmitting the revised manuscript along with the required files:

- A marked-up copy of the manuscript highlighting all changes made.
- A clean version of the revised manuscript without tracked changes.
- This rebuttal letter summarizing our response to the review process.

We appreciate the time and effort invested by the reviewers and the editorial team in assessing our manuscript, and we hope that the revised version meets all publication criteria. Please let us know if any further modifications are required.

The authors confirm that this manuscript has not been previously published and is not currently being considered by any other journal. We have not had any prior discussions with a PLOS ONE Board Member regarding the work described in the manuscript. All authors have approved the contents of this paper and have adhered to PLOS ONE's submission policies.

Thank you again for your consideration. We look forward to your positive response.

Best regards,

Heiko Fuchs

Corresponding Author

Contact:

Dr. rer. nat Heiko Fuchs, Laboratory Manager

Research Laboratory Experimental Ophthalmology

Hannover Medical School

Feodor-Lynen Strasse 35

30625 Hannover

Tel.: + 49511 532-7246	Fax: + 49511 532-7276

Email: fuchs.heiko@mh-hannover.de

Additional Contact:

Prof. Dr. med. Carsten Framme, Director of the eye clinic

University Hospital for Ophthalmology

Hannover Medical School

Carl-Neuberg Strasse 1

30625 Hannover

Tel.: + 49511 532-3060	Fax: + 49511 532-3050

Email: [framme.carsten@mh-hannover.de](mailto:framme.carsten@mh-hannover.de)
